# Design, Molecular Docking, Synthesis, Anticancer and Anti-Hyperglycemic Assessments of Thiazolidine-2,4-diones Bearing Sulfonylthiourea Moieties as Potent VEGFR-2 Inhibitors and PPARγ Agonists

**DOI:** 10.3390/ph15020226

**Published:** 2022-02-14

**Authors:** Mohamed A. Abdelgawad, Khaled El-Adl, Sanadelaslam S. A. El-Hddad, Mostafa M. Elhady, Nashwa M. Saleh, Mohamed M. Khalifa, Fathalla Khedr, Mohamed Alswah, AbdElAziz A. Nayl, Mohammed M. Ghoneim, Nour E. A. Abd El-Sattar

**Affiliations:** 1 Department of Pharmaceutical Chemistry, College of Pharmacy, Jouf University, Sakaka 72341, Saudi Arabia; mhmdgwd@ju.edu.sa; 2 Pharmaceutical Medicinal Chemistry & Drug Design Department, Faculty of Pharmacy (Boys), Al-Azhar University, Cairo 11884, Egypt; mohamedkhalifa2321.el@azhar.edu.eg (M.M.K.); fkhedr2020@gmail.com (F.K.); 3 Pharmaceutical Chemistry Department, Faculty of Pharmacy, Heliopolis University for Sustainable Development, Cairo 11785, Egypt; 4 Pharmaceutical Chemistry Department, Faculty of Pharmacy, Omar Almukhtar University, Al Bayda 991, Libya; elhddad@yahoo.com; 5 Department of Biochemistry, Faculty of Science, Ain Shams University, Abbassia, Cairo 11566, Egypt; m_elhady@sci.asu.edu.eg; 6 Department of Chemistry, Faculty of Science, Al-Azhar University (Girls Branch), Cairo 11754, Egypt; drnashwamostafa@azhar.edu.eg; 7 Pharmaceutical Organic Chemistry Department, Faculty of Pharmacy, Al-Azhar University, Nasr City, Cairo 11884, Egypt; mohamedalswah.el20@azhar.edu.eg; 8 Department of Chemistry, College of Science, Jouf University, Sakaka 72341, Saudi Arabia; aanayel@ju.edu.sa; 9 Department of Pharmacy Practice, College of Pharmacy, AlMaarefa University, Ad Diriyah 13713, Saudi Arabia; mghoneim@mcst.edu.sa; 10 Department of Chemistry, Faculty of Science, Ain Shams University, Abbassia, Cairo 11566, Egypt; nourel-dinahmed@sci.asu.edu.eg

**Keywords:** anti-hyperglycemic, VEGFR-2 inhibitors, docking, PPARγ, sulfonylthiourea, thiazolidine-2,4-dione

## Abstract

Newly designed thiazolidine-2,4-diones **3**–**7a**–**c** were synthesized, and their anticancer activities were screened against three cancer lines. They showed potent activities against HepG2 compared to the other HCT116 and MCF-7 tumor cell lines. Compounds **7c** and **6c** were detected as highly effective derivatives against MCF-7 (IC_50_ = 7.78 and 8.15 µM), HCT116 (IC_50_ = 5.77 and 7.11 µM) and HepG2 (IC_50_ = 8.82 and 8.99 µM). The highly effective derivatives **6a**–**c** and **7a**–**c** were tested against VERO normal cell lines. All derivatives were evaluated for their VEGFR-2 inhibitory actions and demonstrated high to low activities, with IC_50_ values varying from 0.08 to 0.93 µM. Moreover, derivatives **5a**–**c**, **6a**–**c** and **7a**–**c** were assessed to verify their in vitro binding affinities to PPARγ and insulin-secreting activities. Finally, docking studies were performed to explore their affinities and binding modes toward both VEGFR-2 and PPARγ receptors.

## 1. Introduction

Thiazolidinediones are widely found throughout nature in various forms. Thiazolidinedione nucleus is present in numerous biological compounds, e.g., antidiabetic [1], anticancer [2], anti-malarial, antimicrobial, anti-mycobacterium [3], anticonvulsant [4], antiviral, anti-HIV (human immunodeficiency virus) [5], anti-inflammatory [6] and antioxidant agents [7].

Thiazolidine-2,4-diones (TZDs) have been described to have anticancer effects in a broad range of cancers [8,9,10]. TZDs are PPARγ (PPAR-gamma) activators used for type-2 diabetes treatment. Recently, PPAR gamma ligands (TZDs) were proved to exhibit anticancer effects by disturbing cell differentiation, proliferation, and cycle and apoptosis, and were also proven to hinder tumor angiogenesis. The antiangiogenic activity of TZDs is attributed to its inhibition of endothelial cell proliferation and reduction of the production of vascular endothelial growth factors. As it is assumed that the anticancer activity of TZDs is mediated through PPARγ activation, they have been clinically tested against human cancers that express high levels of PPARγ [11,12].

The well-known PPAR-γ agonist rosiglitazone (RGZ) (I) has been widely clinically used because of its significant function lipid, glucose metabolism and energy homeostasis regulation. PPAR-γ is broadly spread in HepG2 cells. RGZ was confirmed to present a transcription factors activity regulator that is critical for apoptosis. In addition, RGZ was used in leukemia and lung cancer cells to induce apoptosis [13].

PPAR-γ is expressed in both malignant and normal mammary epithelial cells. Breast carcinoma proliferation was suppressed by TZDs in vitro and in experimental animals. In addition, the conjugated linoleic acid activation of PPAR-γ has an antiproliferative effect in MCF7 breast cancer. Moreover, the preponderance of evidence among studies investigating the effects of TZDs against breast cancer suggests that both in vivo and in vitro apoptosis induction and inhibition of angiogenesis, proliferation, and invasion are performed by PPAR-γ ligands. In colon cancer, the TZD, pioglitazone (II), inhibited the cell proliferation in a dose-dependent manner [14].

**Figure 1:**
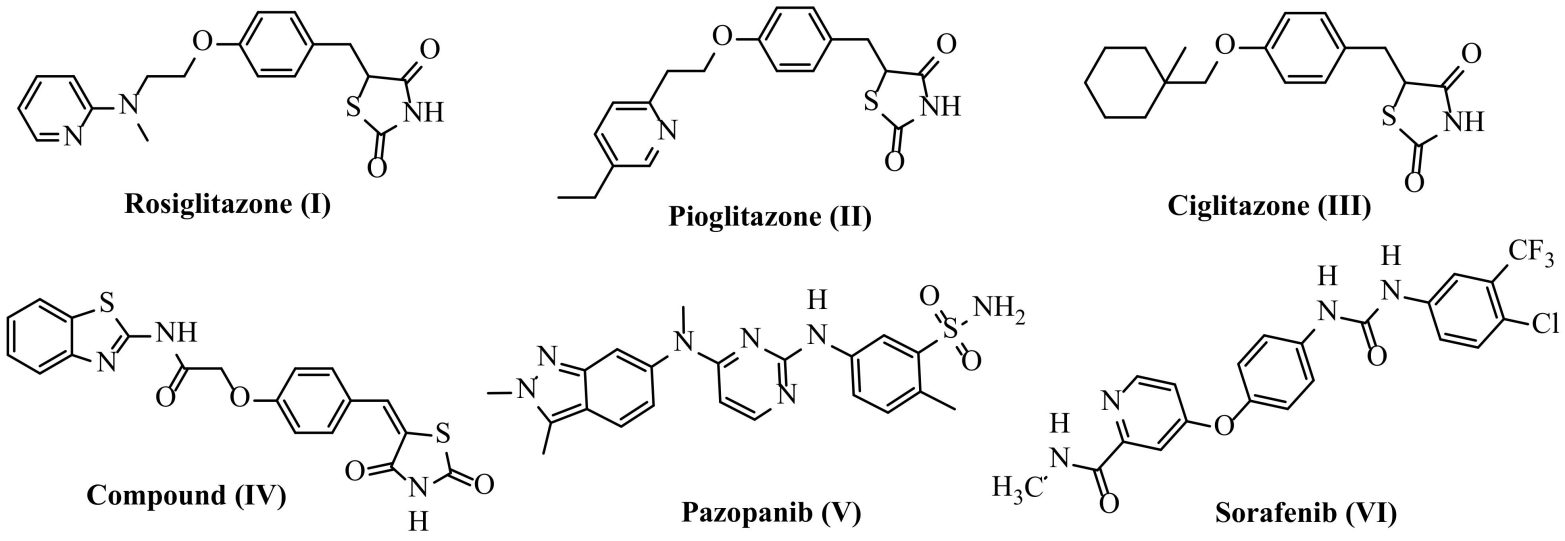
Approved inhibitors of VEGFR-2.

Rosiglitazone (I) and pioglitazone (II) suppress the expression of VEGF via a responsive element for PPAR-γ in the VEGF gene promoter [15]. However, several TZD derivatives, e.g., ciglitazone III and compound (IV), have also been reported to be effective as antiangiogenic and antineoplastic agents through VEGFR-2 targeting [16,17,18,19,20,21,22,23,24] by reducing the in vitro model of VEGF production [20]. The VEGFR-2 receptor is the most important antiangiogenic target due to its crucial role in cancer angiogenesis. Several effective inhibitors of VEGFR-2 have been developed and approved as antiangiogenic agents for different cancer medicines, for example, pazopanib (V) [25,26] and sorafenib (Nexavar)^®^ (VI) [27,28,29] (Figure 1).

**Figure 2:**
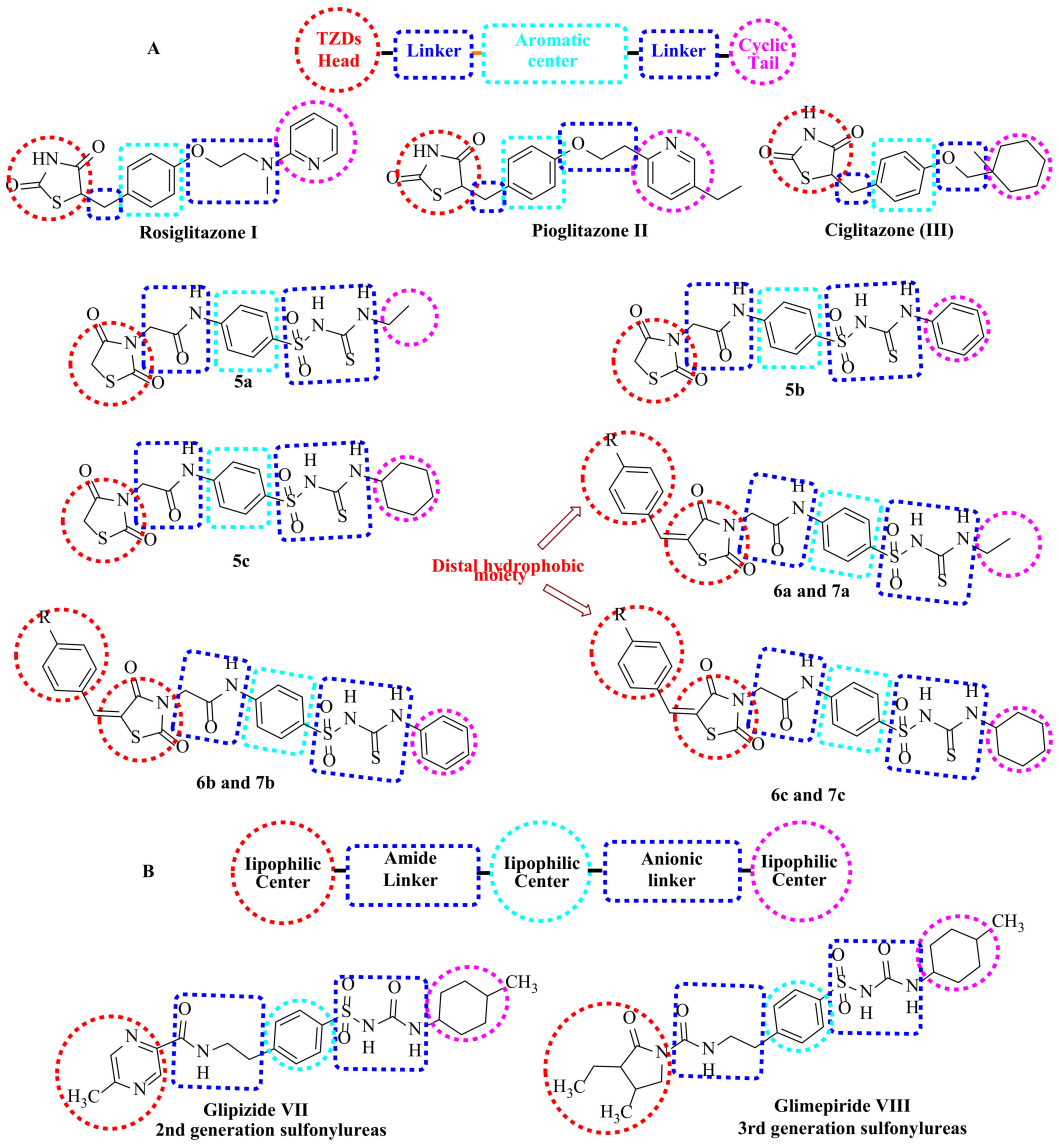
(**A**) The basic structural requirements of PPARγ agonists. (**B**) The basic structural features of sulfonylurea agonists.

Pancreatic β-cells contain sulfonylurea receptors, which are the second receptor involved in diabetes management. The binding of Sulfonylureas (SUs) and sulfonylthioureas with sulfonylurea receptors stimulates insulin secretion [30,31]. Glipizide (VII) and glimepiride (VIII) (Figure 2) are sulfonylureas containing an amidic group which interacts with SURs B site [32]. Moreover, some second- and third-generation sulfonylureas have been reported to act through SURs and PPARγ to exert their clinical efficacy [33,34,35]. 

According to the abovementioned facts, and to obtain novel multi-target anti-cancer drugs, a new series of thiazolidinediones-sulfonylthiourea hybrid was synthesized as potent PPARγ agonists and VEGFR-2 inhibitors. Moreover, the same hybrids were designed as dual SURs and PPARγa agonists for antihyperglycemic activity.

### 1.1. Structure-Based Design Rationale 

#### 1.1.1. As Anticancer Agents

**Figure 3:**
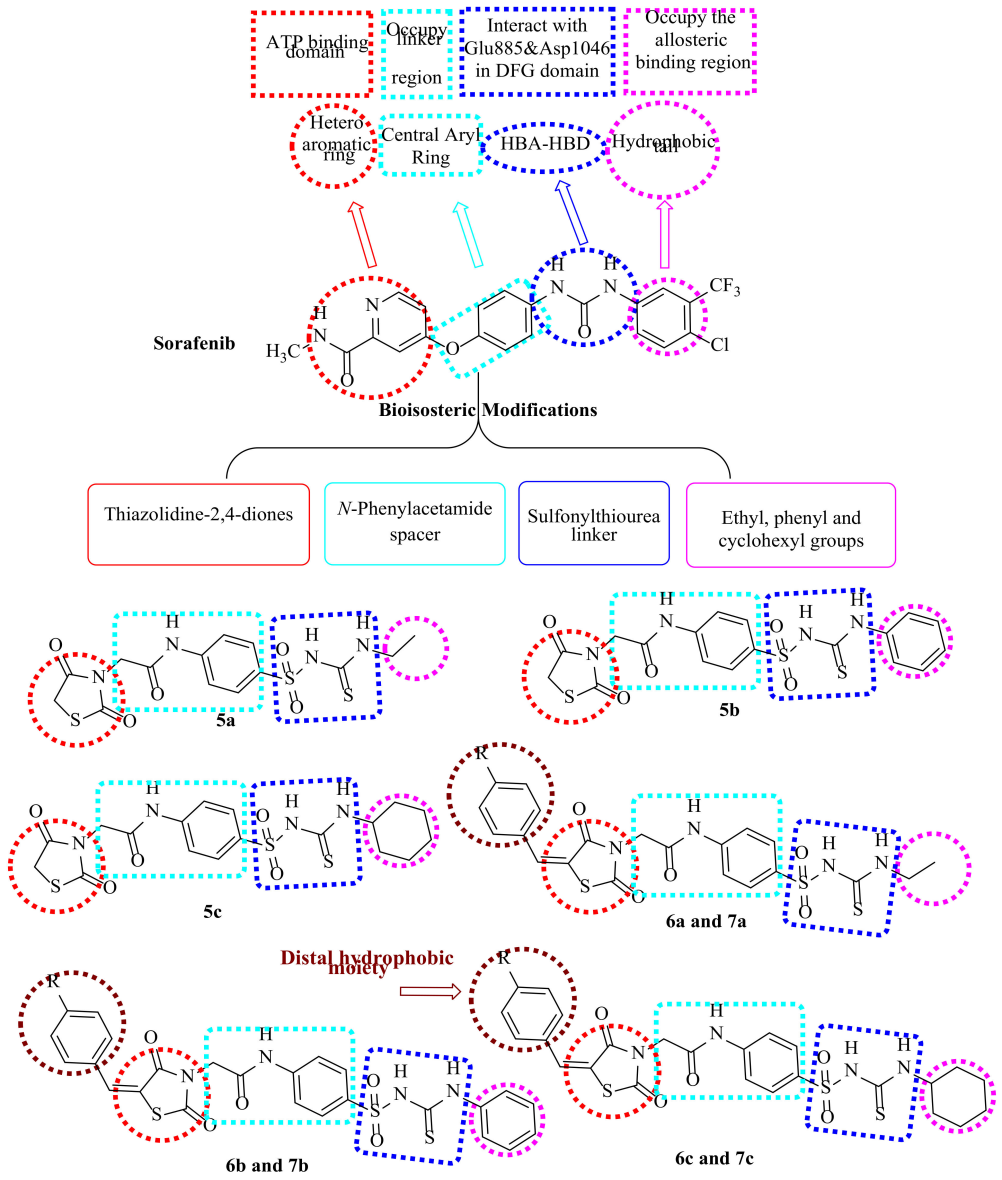
Pharmacophoric requirements and structural similarities of VEGFR-2 inhibitors and our derivatives.

In continuance of our earlier works in the scope of the design and syntheses of novel anticancer medicines [36,37,38,39,40,41,42,43,44], particularly VEGFR-2 inhibitors [45,46,47,48,49,50,51,52,53,54], thiazolidine-2,4-diones bearing sulfonylthiourea moieties were synthesized to obtain the four keys of VEGFR-2 inhibitors pharmacophoric features (Figure 3) [55,56,57]. The focus of the current study was to utilize the lead modification approach for sorafenib, a potent VEGFR-2 inhibitor, to obtain novel potent inhibitors. Modification was selected to cover the four main parts of sorafenib, with the aim of obtaining strongly active derivatives. The first site of modification was the “hinge-binding” head, in which the sorafenib pyridine ring was modified to a thiazolidine-2,4-dione one. Regarding the “Linker” part, an N-phenylacetamide moiety was the central aryl linker ring used in our design. With respect to the “hydrogen-bonding moiety,” the urea moiety of sorafenib was herein extended to be a sulfonylthiourea target to enhance VEGFR-2 binding affinities. Lastly, the hydrophobic tail of sorafenib was substituted by other different hydrophobic groups, including ethyl, phenyl and/or cyclohexyl groups.

Finally, examining several inhibitors bound to VEGFR-2, X-ray analysis proved the presence of an additional available space for various substituents around the terminal hetero aromatic ring [58,59]. This finding encourages us to design extra phenyl and/or 4-methylphenyl groups to occupy this space, aiming to increase the binding activities with the receptor as in compounds **6a**–**c** and **7a**–**c**, respectively.

#### 1.1.2. As Antidiabetic Agents

The basic structural requirements of PPARγ are similar to that of SUR agonists, which drove us to design newthiazolidine-2,4-diones bearing sulfonylthiourea comprising both requirements (Figure 2). The thiazolidine-2,4-diones head is required for PPARγ agonistic activity. Sulfonylthiourea moieties were introduced to our derivatives, providing both SUR and PPARγ agonistic actions [60]. Aliphatic and aromatic substituents on sulfonylthiourea moieties act as lipophilic centers essential for agonistic action on SUR. Furthermore, the sulfamoyl NH group is completely ionized at physiological pH due to its acidity (pKa = 4.9–6.5) [61]. This ionization provides the anionic linker that is markedly required for SUR agonists [62]. Different linkers between the aromatic moieties (spacer groups) and lipophilic tails were prepared to study their effects on SAR of the new derivatives. These linkers are also important for the agonistic action of PPARγ. On the other hand, they contain the amide(-CONH-) group necessary for interaction with the SURs B site [56]. 

Based on previous findings, new thiazolidine-2,4-diones-sulfonylthiourea hybrids were designed and synthesized to obtain new multi-target antihyperglycemic agents (Figure 2). 

## 2. Results and Discussion

### 2.1. Chemistry 

**Scheme 1:**
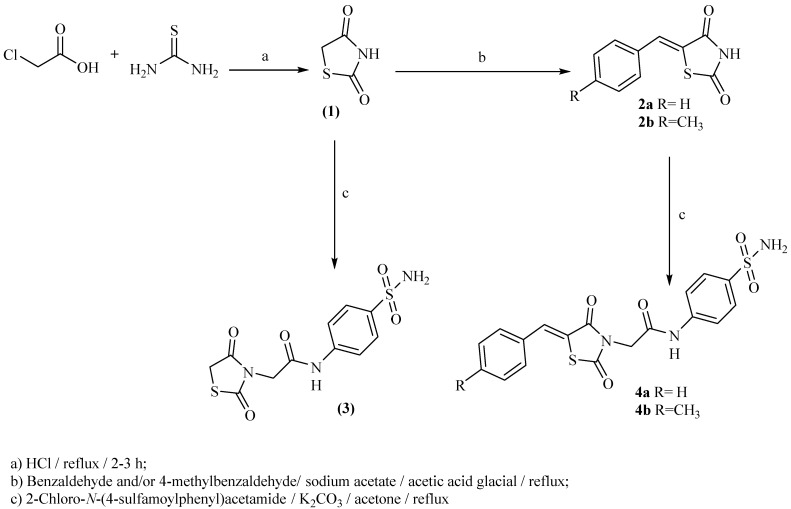
The target compounds **3**–**4a**,**b** synthetic pathway.

**Scheme 2:**
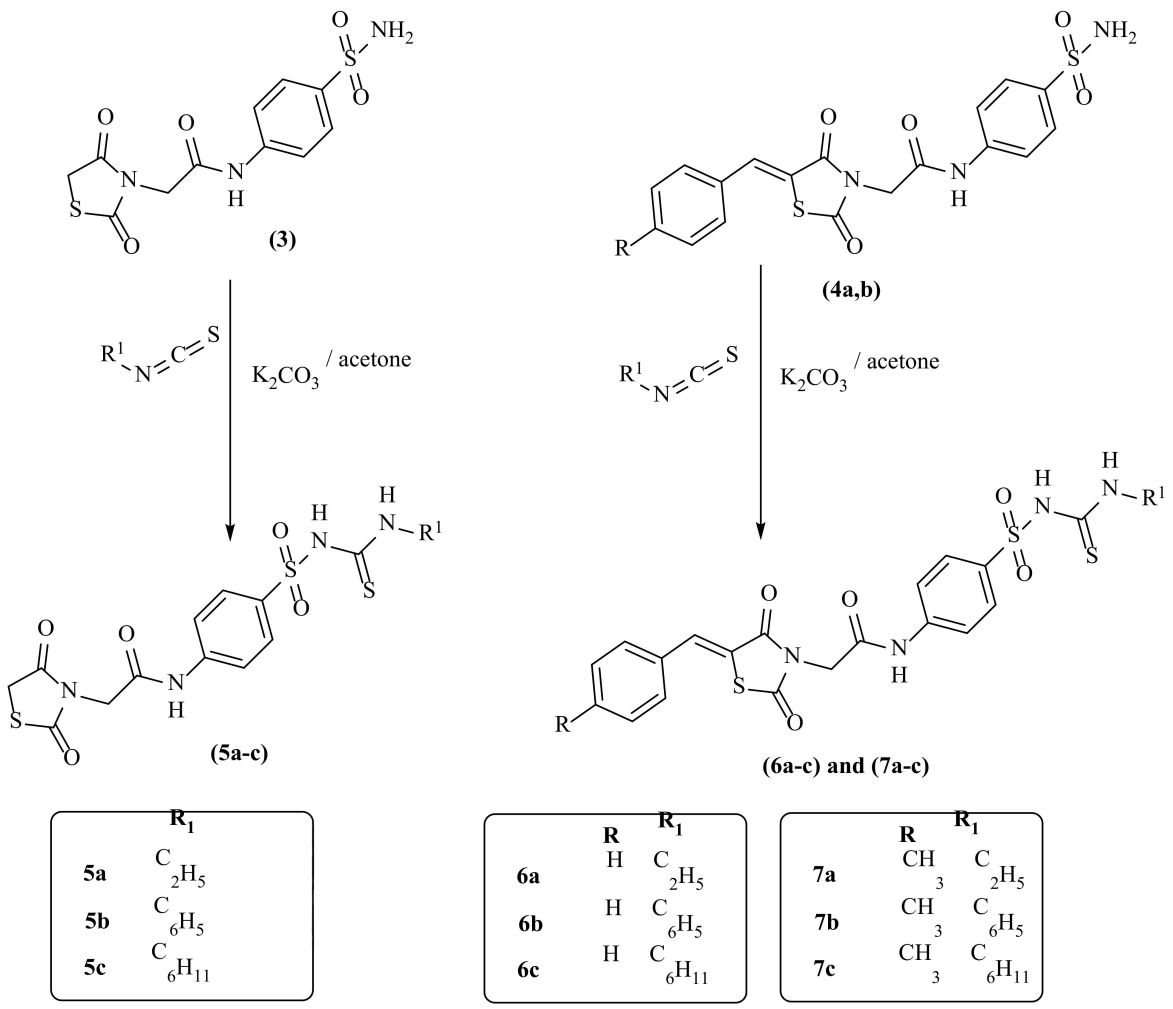
The target compounds **5**–**7a**–**c** synthetic pathway.

In Scheme 1 and Scheme 2, the reactions sequence for the preparation of our target compounds is demonstrated. The sequence starts with chloroacetic acid and thiourea cyclocondensation to obtain thiazolidine-2,4-dione (1) [16,17,18], which undergoes Knoevenagel condensation [16,17,18] to provide the corresponding derivatives (**2a**,**b**). The heating of (1) and/or **2a**,**b** with 2-chloro-N-(4-sulfamoylphenyl)acetamide under reflux provided the corresponding acetamide derivatives **3** and/or **4a**,**b**, respectively (Scheme 1). On the other hand, 2-chloro-N-(4-sulfamoylphenyl)acetamide was synthesized according to the directions of Jacobs and Heidelberger [63]. Subsequent heating of **3** and/or **4a**,**b** with the appropriate isothiocyanate under reflux provided the corresponding sulfonylthiourea derivatives **5a**–**c**, **6a**–**c** and/or **7a**–**c**, respectively (Scheme 2).

### 2.2. Docking Studies

Molsoft software was applied for the molecular docking studies. All experiments utilized VEGFR-2 and PPARγ (PDB ID 4ASD) [64], and (PDB ID 3CS8) [65], respectively.

#### 2.2.1. Docking Studies as VEGFR-2 Inhibitors

**Figure 4:**
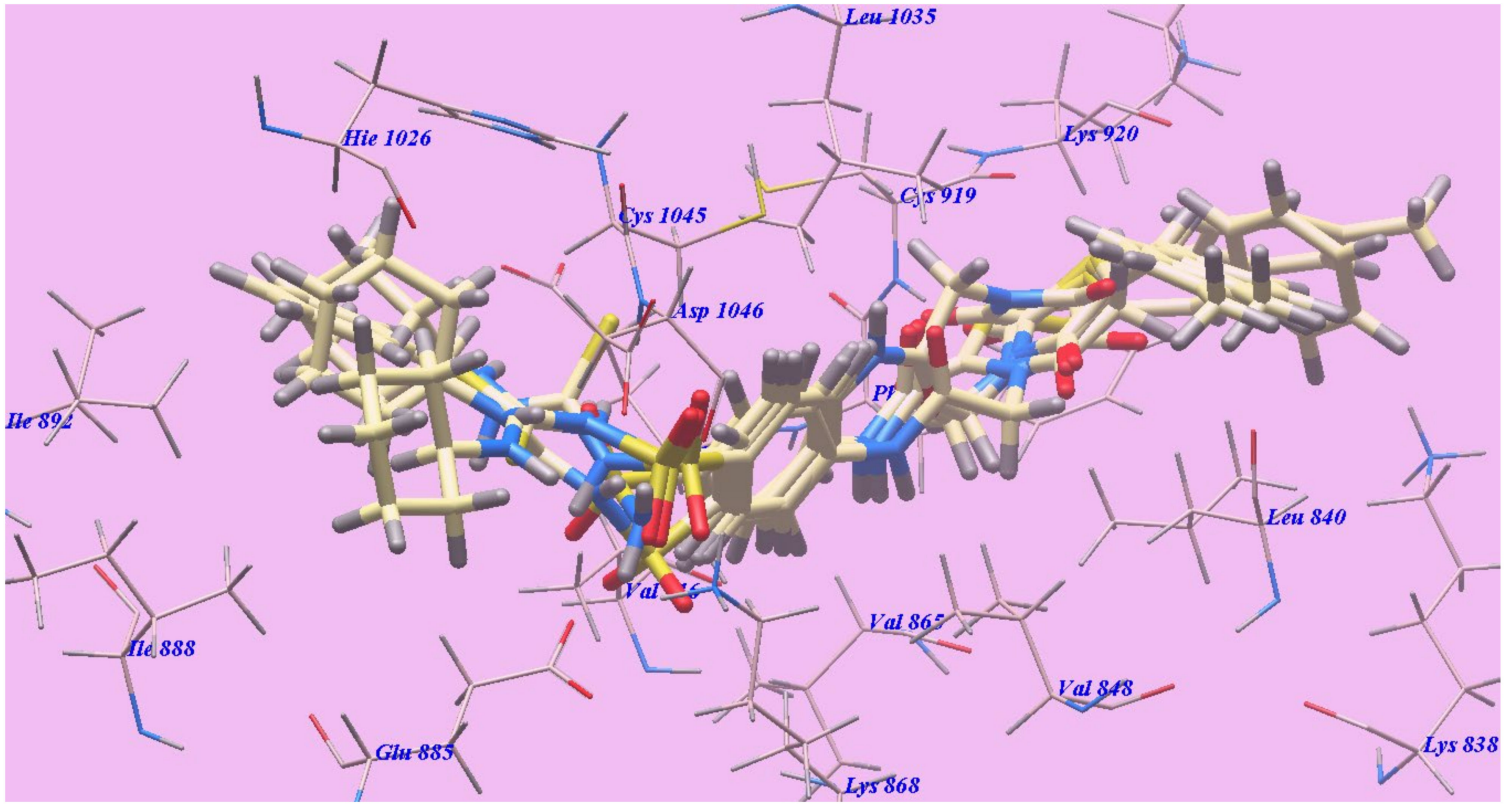
Superimposition of compounds **4b**, **6b**, **6c**, **7b** and **7c** inside the active site of 4ASD.

The achieved results showed that all studied congeners showed similar orientations and positions inside the identified VEGFR-2 active site (Figure 4). Calculating the binding free energies of (∆G) of the docked members explained their high binding affinities to the receptor, and the total trend was indicated by the calculated values (Table 1).

**Figure 5:**
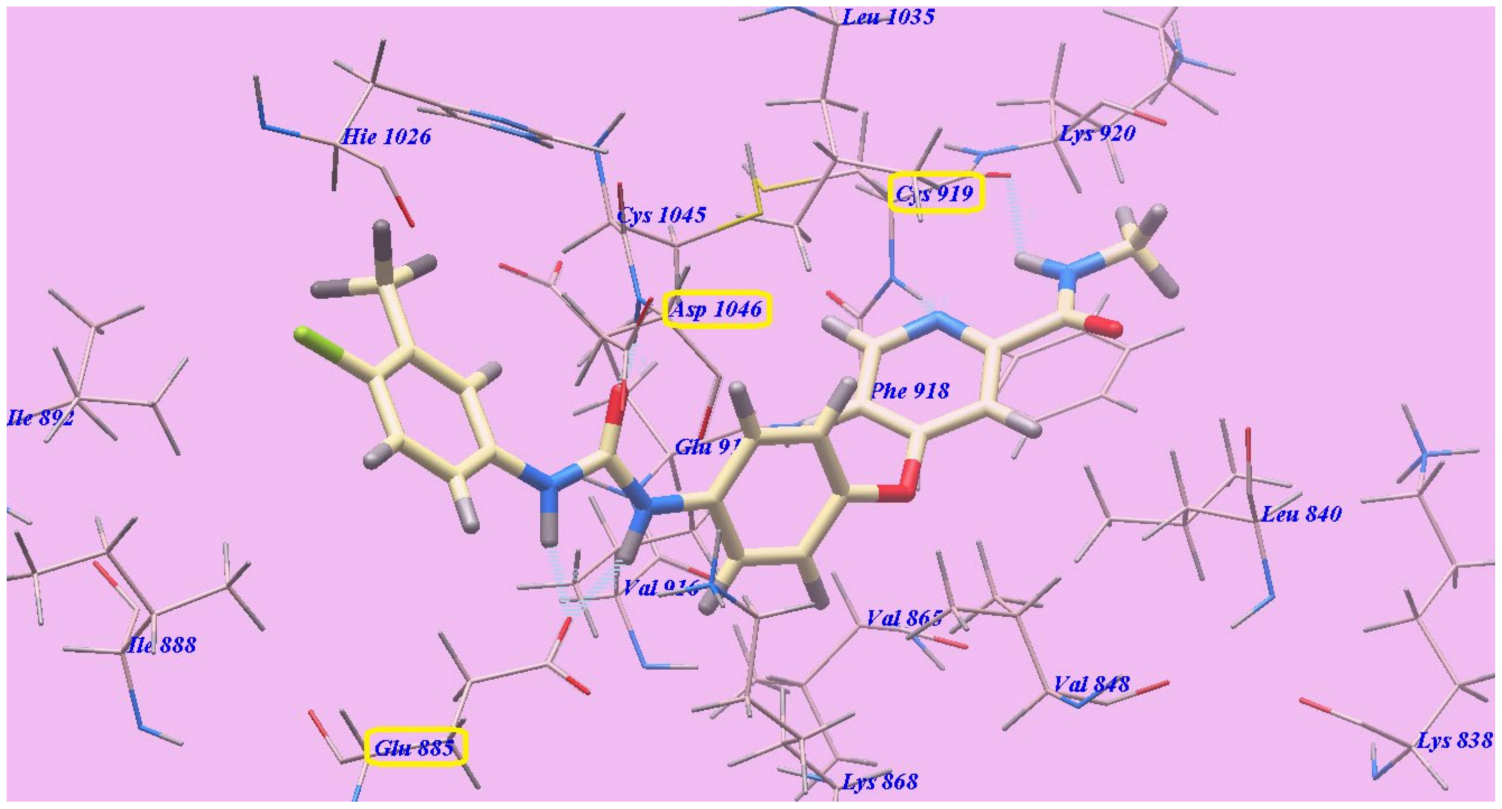
Sorafenib with the 4ASD predicted binding mode. H-bonded atoms are designated by dotted lines.

The suggested binding mode of sorafenib showed an affinity value of −110.12 kcal/mol and formed five H-bonds. It formed two H-bonding interactions with Cysteine919 (2.51 Å and 2.10 Å), two H-bonds with Glutamate885 (1.77 Å and 2.75 Å) and one H-bonding interaction with Aspartate1046 (1.50 Å). The N-methylpicolinamide group occupied the pocket produced by Leucine1035, Lysine920, Cysteine919, Phenylalanine918, Glutamate917, Valine848 and Leucine840. Furthermore, the central phenyl linker occupied the hydrophobic groove produced by Cysteine1045, Leucine1035, Threonine916, Lysine868 and Valine848. In addition, the terminal 3-trifluromethyl-4-chlorophenyl group occupied the hydrophobic channel formed by Aspartate1046, Cysteine1045, Histidine1026, Isoleucine892, Isoleucine888 and Glutamate885 (Figure 5). The urea linker had a significant function in the binding with VEGFR-2 enzyme. However, the linker was responsible for the high binding affinity of sorafenib. These conclusions led us to use the sulfonythiourea linker to obtain effective VEGFR-2 inhibitors.

**Figure 6:**
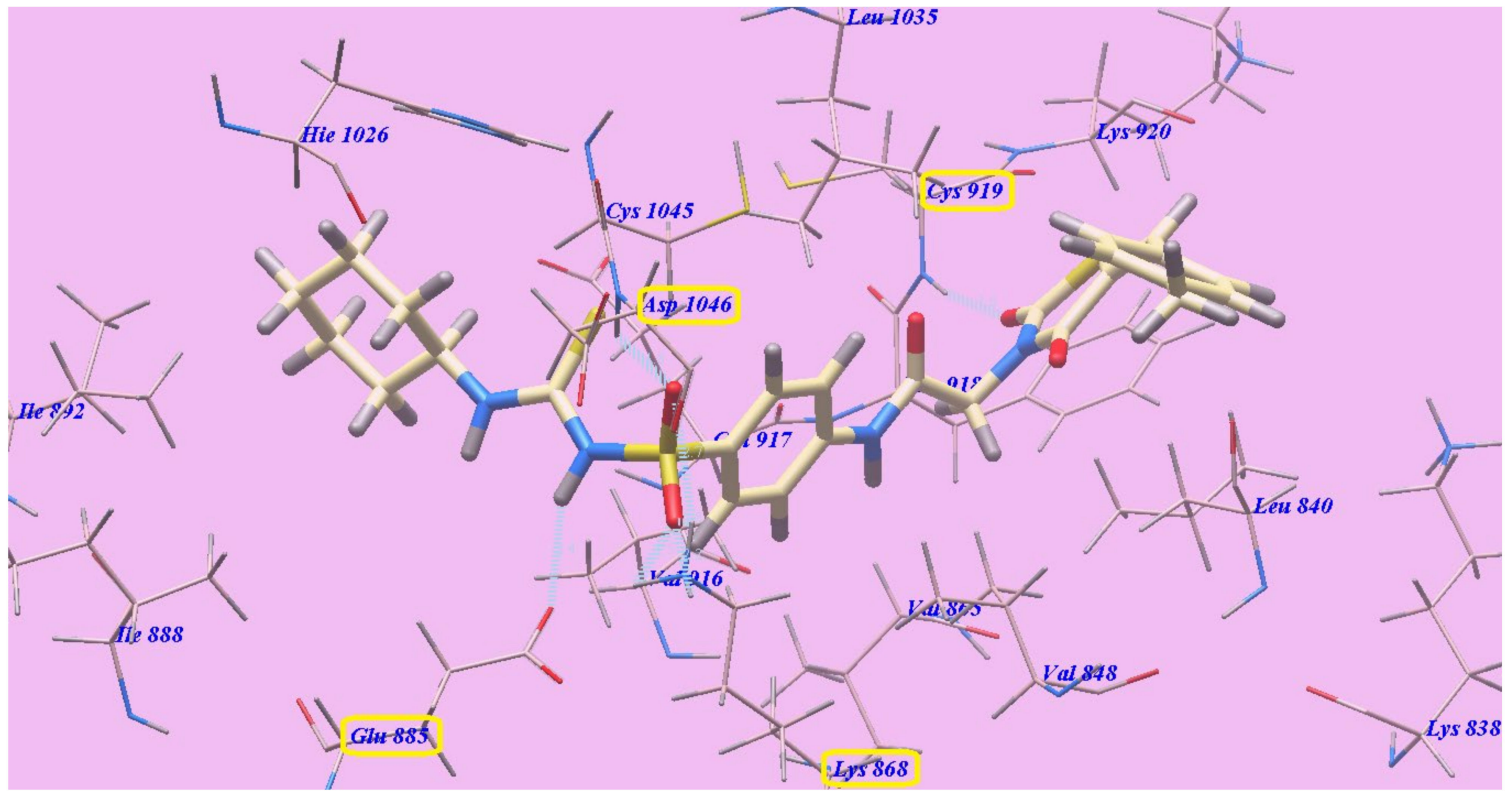
**7c** with the 4ASD predicted binding mode.

Compound **7c** and sorafenib had virtually the same binding mode, which showed seven H-bonds and an affinity value of −138.79 kcal/mol. The sulfone moiety of the sulfonylthiourea linker was stabilized by the construction of one H-bond with Aspartate1046 (1.82 Å) and four H-bonds with Lysine868 (0.83 Å, 1.50 Å, 2.21 Å and 2.66 Å), but its NH group formed another H-bond with Glutamate885 (2.38 Å). Moreover, the C=O group at position-2 of the thiazolidine-2,4-dione moiety established one H-bond with Cysteine919 (1.59 Å). The 4-methylphenylmoiety occupied the hydrophobic space around the terminal thiazolidine-2,4-dione ring formed by Lysine920, Leucine840 and Lysine838. Furthermore, the thiazolidine-2,4-dione group inhabited the hydrophobic ATP binding pocket produced by Leucine1035, Lysine920, Cysteine919, Phenylalanine918 and Leucine840. The central hydrophobic phenyl resides in the hydrophobic pocket produced by Aspartate1046, Cysteine1045, Leucine1035, Valine916, Lysine868 and Valine865. Moreover, the distal cyclohexyl cycle inhabited the hydrophobic groove produced by Cysteine1045, Histidine1026, Isoleucine892, Isoleucine888 and Glutamate885 (Figure 6). Compound **7c** interactions may clarify its greatest anticancer action.

**Figure 7:**
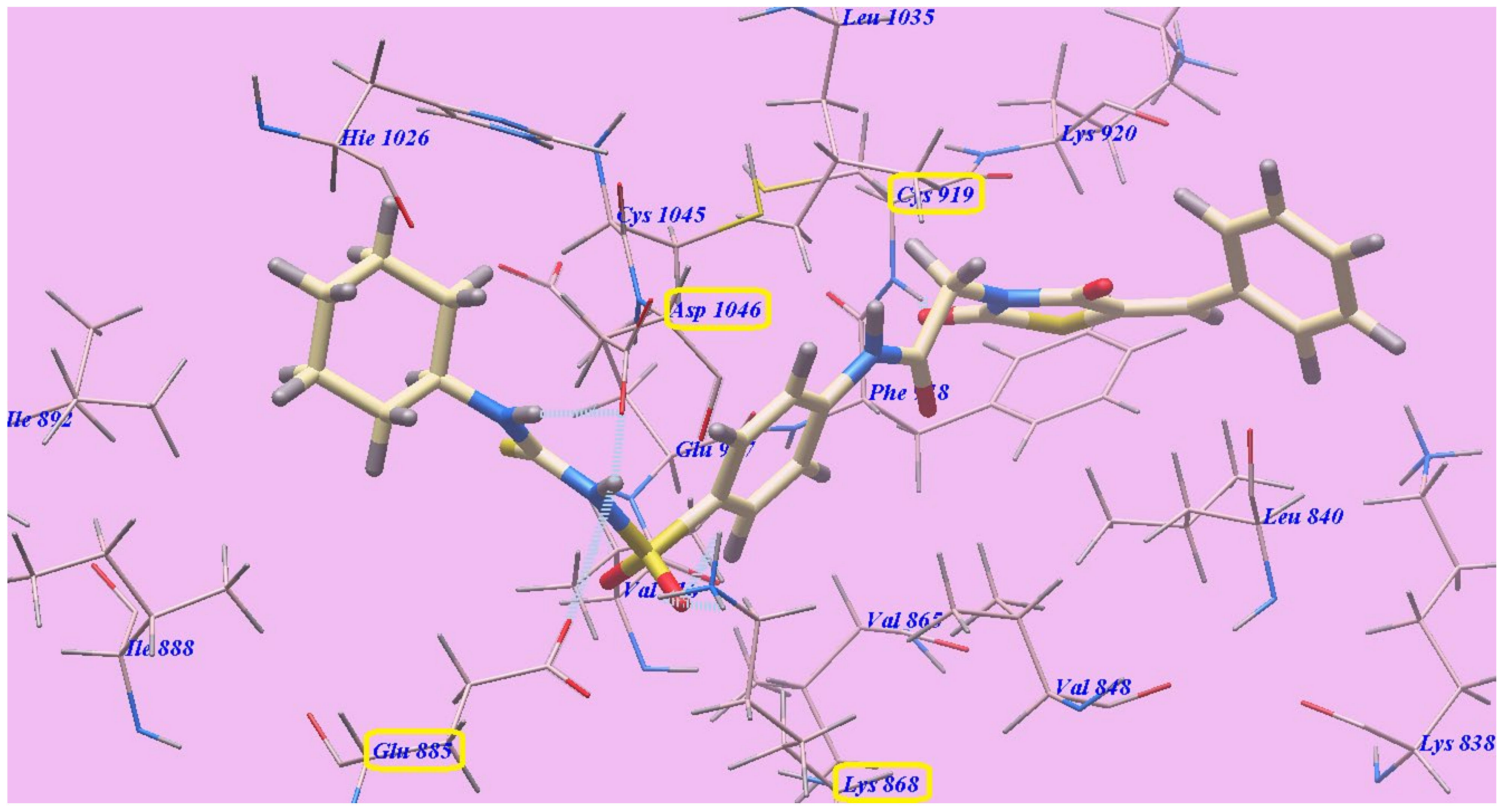
**6c** with the 4ASD predicted bind mode.

The suggested **6c** binding mode was similar to **7c**, with −130.36 kcal/mol. **6c** formed six H-bonds with Lysine868 (1.84 Å and 2.52 Å), Aspartate1046 (2.17 Å and 2.92 Å), Glutamate885 (2.73 Å) and Cysteine919 (1.81 Å) (Figure 7).

The obtained docking results (Table 1) revealed that the sulfonylthiourea linkers played an essential role in the greater affinities for the VEGFR-2 enzyme. The affinities toward the VEGFR-2 enzyme were increased as result of the benzylidene hydrophobic interactions. The thiazolidine-2,4-dione facilitated the novel targets to form H-bonds with Cysteine919. Structure extension was an essential factor in the inhibitory action to VEGFR-2.

#### 2.2.2. Docking Studies as PPARγ Agonists

**Figure 8:**
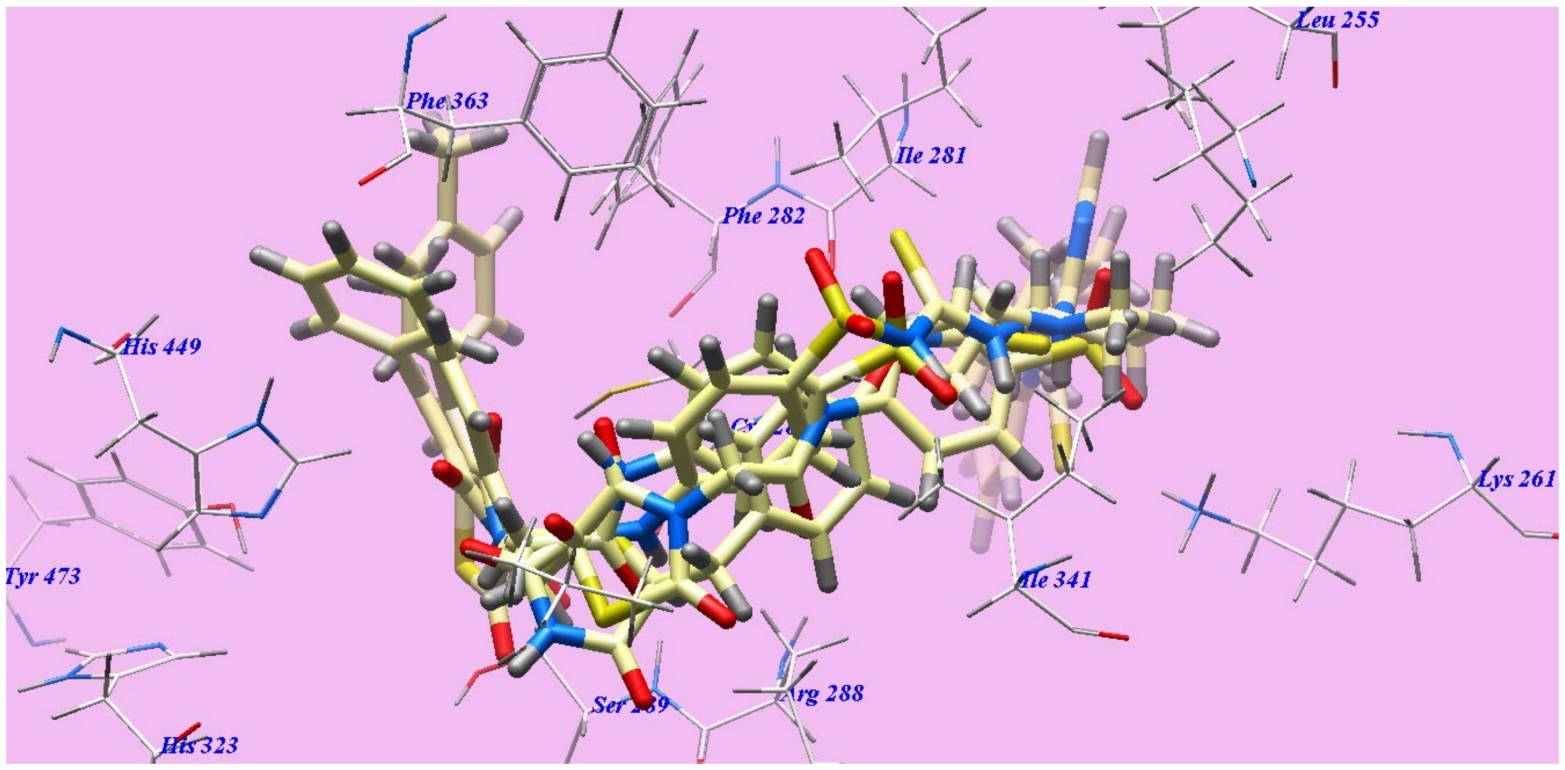
Rosiglitazone, **6c**, **7b** and **7c** docked compounds superimposition in the active site of 3CS8.

The achieved results revealed that all studied compounds exhibited similar orientations and positions within the identified binding site of PPARγ (Figure 8). The results of the binding free energy (∆G) showed that the majority of these compounds showed high receptor binding affinities, and the computed values indicated a global trend (Table 2).

**Figure 9:**
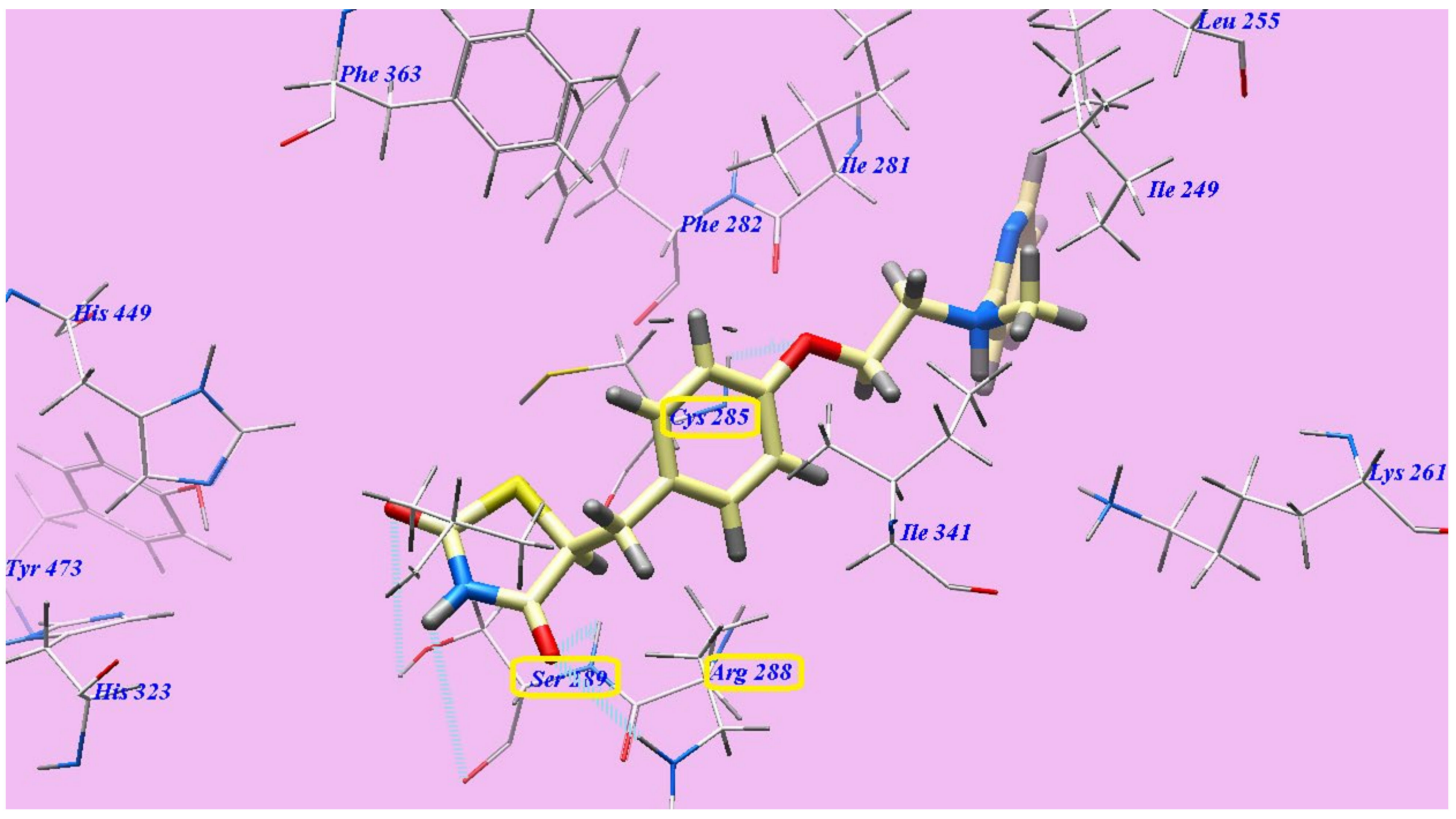
Rosiglitazone and 3CS8 expected binding mode. The dotted lines indicate H-bonded atoms.

The suggested binding manner of rosiglitazone showed an affinity value of −94.38 kcal/mol and five H-bonds. The thiazolidine-2,4-dione established three H-bonds with Serine289 (2.83 Å, 2.91 Å and 2.97 Å) and one H-bond with Arginine288 (2.44 Å). The linker oxygen atom formed one H-bond with Cysteine285 (2.96 Å). The central phenyl inhabited the hydrophobic channel produced by Arginine288, Cysteine285, Phenylalanine363, Phenylalanine282 and Isoleucine341. Moreover, the aminoethoxy linker inhabited the hydrophobic pocket produced by Phenylalanine282, Isoleucine281 and Isoleucine341. In addition, the cyclic pyridine tail inhabited the hydrophobic pocket produced by Isoleucine281, Isoleucine341, Lysine261 and Isoleucine249 (Figure 9).

**Figure 10:**
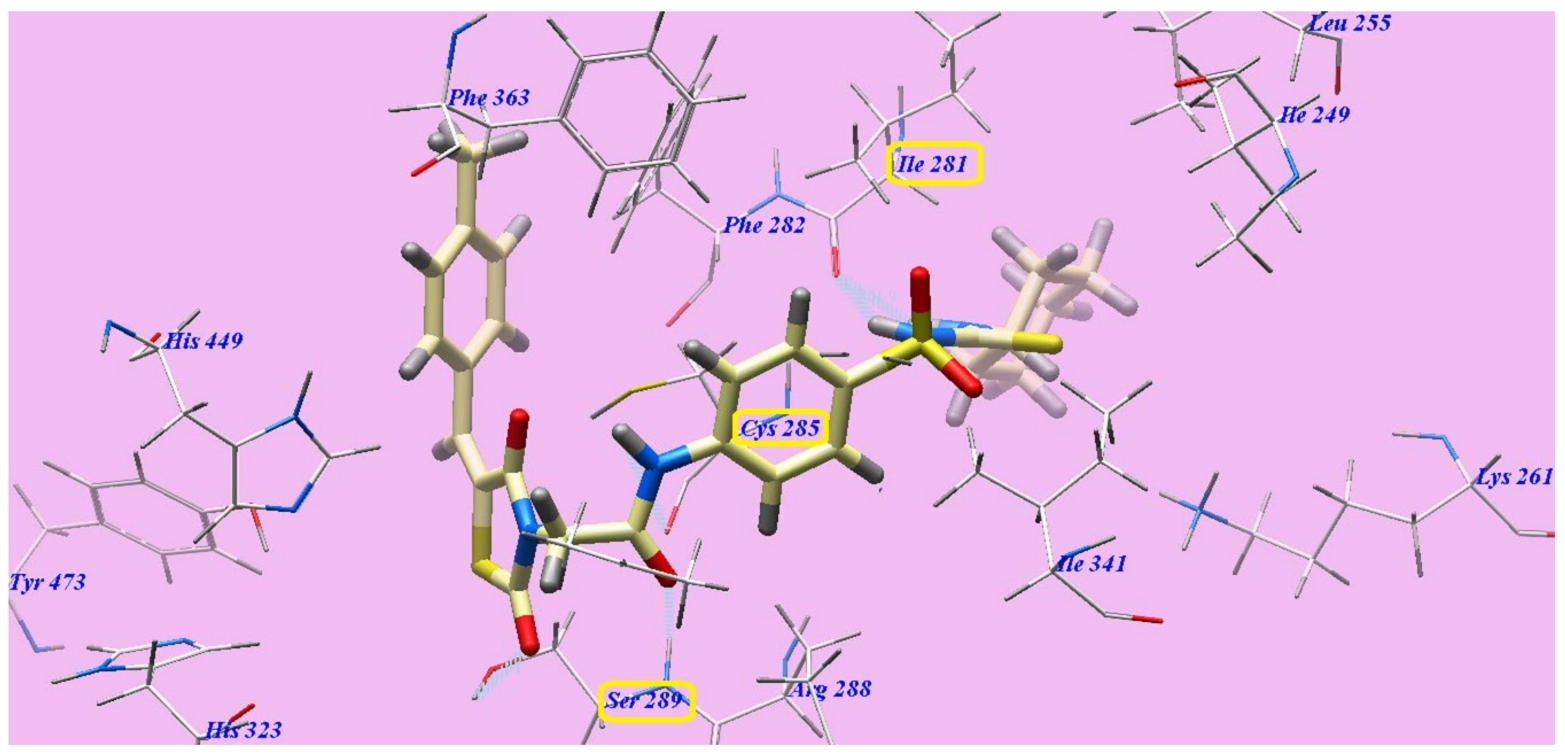
**7c** with the 3CS8 predicted binding mode.

Compound **7c** represented nearly a similar binding mode as rosiglitazone, which showed an affinity value of −132.78 kcal/mol and five H-bonds. It formed two H-bonding interactions with Serine289 (2.09 Å and 2.96 Å), one H-bond with Cysteine285 (2.47 Å) and two H-bonds with Isoleucine281 (1.92 Å and 2.49 Å). The 4-methylphenyl moiety was placed in the hydrophobic space around the terminal thiazolidine-2,4-dione ring formed by Phenylalanine363, Histidine449 and Phenylalanine282. The central hydrophobic phenyl ring was located in the formed hydrophobic pocket by Arginine288, Cysteine285, Phenylalanine363, Phenylalanine282 and Isoleucine341. Furthermore, the cyclohexyl tail was positioned in the hydrophobic furrow formed by Isoleucine281, Isoleucine341, Lysine261 and Isoleucine249 (Figure 10).

**Figure 11:**
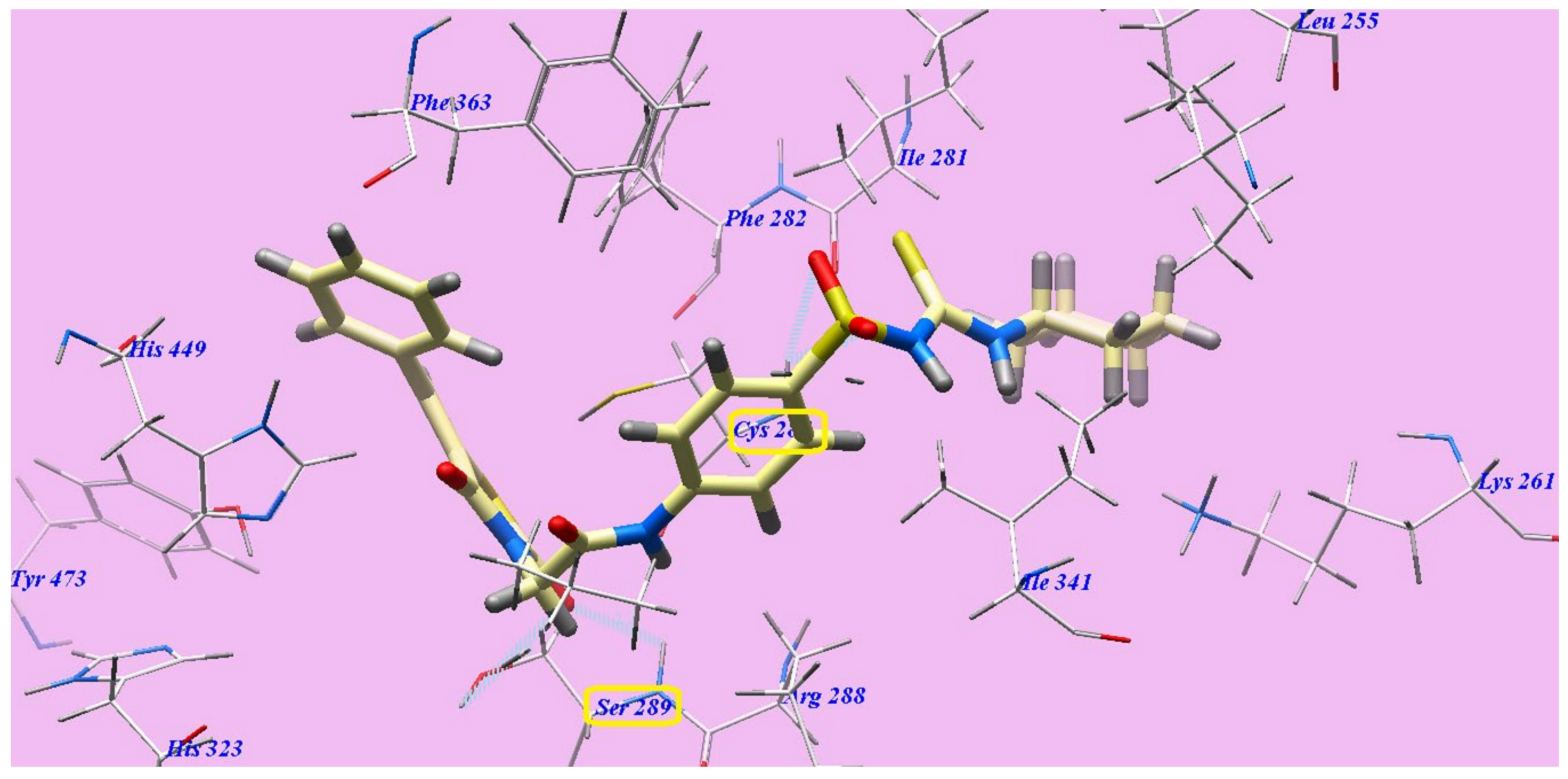
**6c** with the 3CS8 suggested binding mode.

Compound **6c** had virtually the same binding mode as **7c**, with −128.20 kcal/mol. Compounds **6c** and **7c** and formed four H-bonds with Serine289 (2.78 Å and 2.98 Å) and one H-bond with Cysteine285 (2.66 Å, 2.96 Å) (Figure 11).

From the achieved results of docking (Table 2), we assumed that the sulfonylthiourea linkers displayed an essential role for higher affinities toward the PPARγ enzyme. In addition, the benzylidene moieties enhanced hydrophobic interactions and, accordingly, affinities for the PPARγ enzyme.

### 2.3. In Vitro Cytotoxic Activity

The novel prepared thiazolidine-2,4-diones **3**–**7a**–**c**, antiproliferative activity was inspected against three human tumor cell lines—MCF-7, HepG2 and HCT-116—by means of MTT colorimetric assay, as defined by Mosmann [66]. Doxorubicin and sorafenib were included in the experiments as standards. In Table 3, the results are presented as IC_50_ values (50% inhibitory concentration). The achieved findings clarified that the majority of the synthesized congeners exhibited modest to excellent growth inhibitory activity toward the checked tumor cell lines. Generally, observing the cytotoxic activity showed that HepG2 was the most susceptible cell line to the impact of the novel compounds. Compounds **7c** and **6c** were the most effective compounds against the MCF-7 (IC_50_ = 7.78 and 8.15 µM), HCT116 (IC_50_ = 5.77 and 7.11 µM) and HepG2 (IC_50_ = 8.82 and 8.99 µM) tumor cell lines. Compounds **7c** and **6c** exhibited lower activities than sorafenib (IC_50_ = 7.26, 5.47 and 9.18 µM) against MCF-7 and HCT116 but higher activities against HepG2, respectively. However, these compounds demonstrated lower actions than doxorubicin (IC_50_ = 6.75, 8.07 and 7.94 µM) against the three cell lines, respectively. Regarding HepG2, compounds **7b**, **6b**, **7a** and **6a** exhibited the greatest anticancer effects, with IC_50_ = 9.65, 10.67, 12.05 and 14.16 µM, respectively. Derivatives **5c**, **5b** and **5a**, with IC_50_ = 20.75, 21.99 and 24.49 µM, respectively, showed potent cytotoxicity. Derivatives **4b**, **4a** and **3**, with IC_50_ = 48.56, 52.87 and 58.55 µM, respectively, exhibited moderate cytotoxicity.

Derivatives **7b**, **6b**, **7a** and **6a** exhibited the greatest anticancer effects, with IC_50_ = 13.48, 13.78, 16.79 and 17.65 µM, respectively, against HCT-116. Moreover, derivatives **5c** and **5b**, with IC_50_ = 23.56 and 25.68 µM, respectively, showed potent cytotoxic effects. Derivatives **5a**, **4a** and **4b**, with IC_50_ = 40.11, 55.12 and 57.87 µM, respectively, demonstrated moderate cytotoxic action. Derivative 3, with IC_50_ = 61.48 µM, showed the lowest cytotoxic activity.

Derivatives **7b**, **6b**, **6a** and **7a** exhibited the greatest anticancer effects, with IC_50_ = 12.89, 12.95, 16.47 and 16.66 µM, respectively, upon assessment against MCF-7. Derivatives **5b**, **5c** and **5a**, with IC_50_ = 23.24, 24.59 and 28.79 µM respectively, showed great cytotoxic effects. Derivative **4a**, with IC_50_ = 54.99 µM, showed mild cytotoxicity. Derivatives **3** and **4b**, with IC_50_ = 60.18 and 62.43 µM, demonstrated mild cytotoxic action.

In the end, the highly effective candidates, **6a**–**c** and **7a**–**c**, were assessed against VERO normal cell lines to evaluate their cytotoxic effects. The outcomes showed that the assessed candidates possessed weak toxicity against normal VERO cells, with IC_50_ values ranging from 40.88 to 68.25 μM. The cytotoxic effects of the prepared derivatives against the malignant cell lines ranged from 5.77 to 17.65 μM. Derivatives **6a**–**c** and **7a**–**c** were, respectively, 3.41-, 3.83-, 6.93-, 4.99-, 5.45- and 7.74-fold times more toxic against HepG2 than normal VERO cells. Similarly, derivatives **6a**-**c** and **7a**-**c** were, respectively, 2.74-, 2.97-, 6.04-, 3.58-, 3.90- and 11.83-fold more toxic in HCT-116 than in normal VERO cells. Moreover, compounds **6a**–**c** and **7a**–**c** were, respectively, 2.93-, 3.16-, 4.85-, 3.61-, 4.08- and 8.77-fold times higher toxicity in MCF-7 than in normal VERO cells.

### 2.4. In Vitro Assay of VEGFR-2 Kinase 

In addition, our compounds were assessed for their VEGFR-2 inhibitory effects by applying an anti-phosphotyrosine antibody with the Alpha Screen system (PerkinElmer, Waltham, MA, USA) [67,68]. The results are described as IC_50_ (50% inhibition concentration value) in Table 3. In this assessment, sorafenib was applied as a positive standard. The assessed derivatives demonstrated high to low inhibitory effects, with IC_50_ values varying from 0.08 to 0.93 µM. Derivatives **7c** and **6c** were observed to be the highest effective derivatives that inhibited VEGFR-2 at the same IC_50_ = 0.08 µM. Compound **7b** displayed great activity with IC_50_ = 0.11 µM. Moreover, compounds **7a**, **6b** and **6a** possessed high VEGFR-2 inhibition, with IC_50_ = 0.14, 0.15 and 0.17 µM, respectively. Derivatives **5a**–**c** displayed moderate VEGFR-2 inhibitory effects, with IC_50_ = 0.46, 0.44 and 0.44 µM, respectively. Candidates **3**, **4a** and **4b** showed lower VEGFR-2 inhibitory effects, with IC_50_ = 0.93, 0.92 and 0.89 µM, respectively. 

### 2.5. In Vitro Binding Assay of PPARγ Ligand

Derivatives with effective cytotoxic activities (**5a**–**c**, **6a**–**c** and **7a**–**c**) were additionally assessed to evaluate their PPARγ in vitro binding affinities. The binding capabilities of our new target compounds with PPARγ were evaluated using the Fluorescence Polarization Assessment technique [69]. Rosiglitazone was applied as standard with IC_50_ = 0.292. Table 4 shows a comparison of the IC_50_ values of the tested derivatives. Candidates **7c** and **6c** substantially bound to PPARγ, with IC_50_ = 0.296 and 0.300 µM, respectively. Moreover, compounds **6a**, **6b**, **7a** and **7b** exhibited strong binding affinities toward PPARγ, with IC_50_ = 0.323, 0.308, 0.320 and 0.305 µM, respectively. Alternatively, compounds **5a**, **5b** and **5c** moderately demonstrated PPARγ binding affinities, with IC_50_ = 0.393, 0.377 and 0.360 µM, respectively. 

### 2.6. In Vitro Insulin Assay

Compounds **5a**–**c**, **6a**–**c** and **7a**–**c** were also assessed to establish their insulin-secretion activities in vitro against isolated pancreatic islets of rats through the quantitative sandwich technique of enzyme immunoassay [70]. Glimiperide was applied as the standard. The findings are described as the EC_50_ values (Table 4).

The assessed derivatives showed high to moderate insulin-secreting activities within a range of EC_50_ values from 0.70 to 1.20 µM. Glimiperide demonstrated EC_50_ = 0.73 µM. Derivatives **7c** and **6c** displayed effective insulin-secretion activities with the same EC_50_ = 0.70 µM. In addition, compounds **6a**, **6b**, **7a** and **7b** showed good activities, with EC_50_ = 0.87, 0.78, 0.81 and 0.75 µM, respectively. Conversely, derivatives **5a**, **5b** and **5c** showed moderate activities, with EC_50_ = 1.20, 1.13 and 1.00 µM, respectively.

### 2.7. SAR (Structure Activity Relationship)

**Figure 12:**
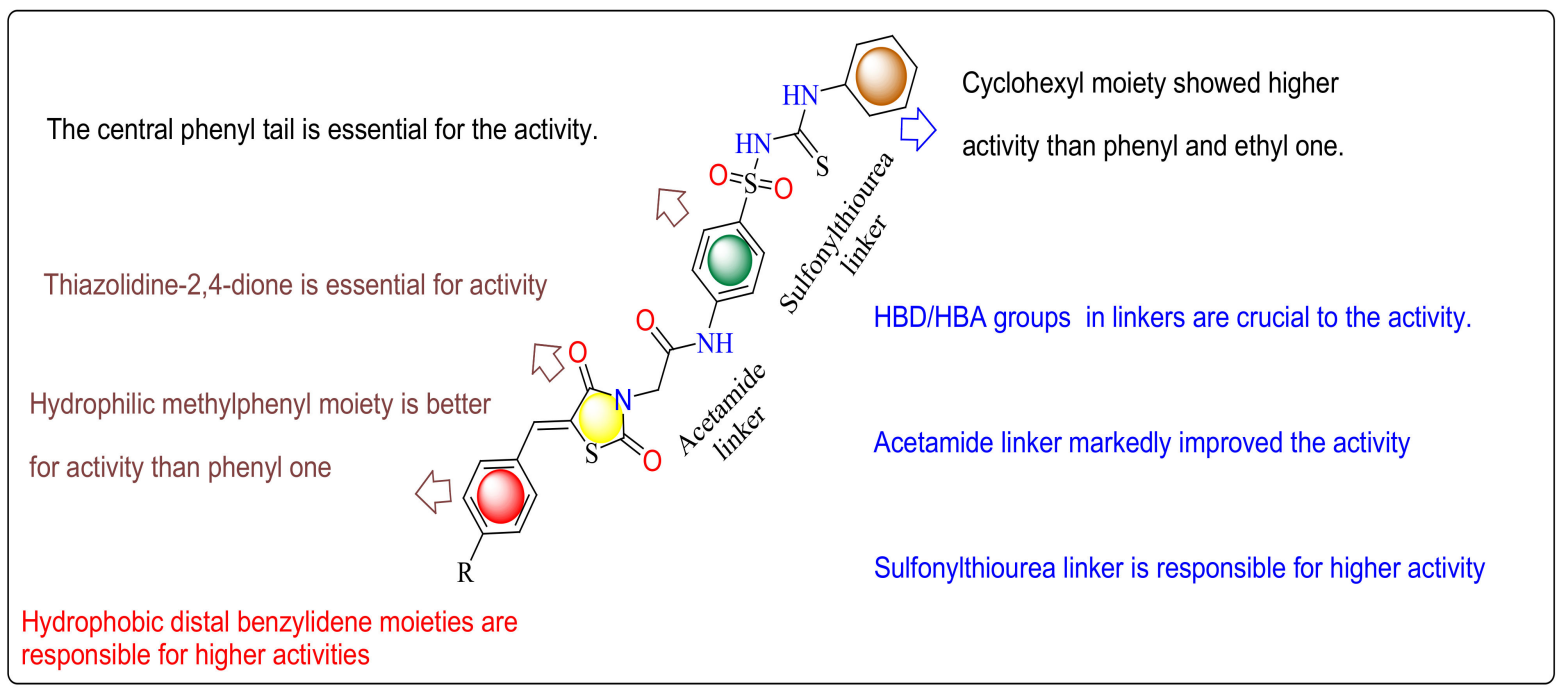
Graphical summary of the SAR study.

The primary SAR analysis concerned the influence of the substitution of sorafenib urea linker by sulfonylthiourea linkers, which acted as H-bond acceptors and H-bond donors. These linkers interacted with Aspartate1044 and Glutamate883. Similarly, hydrophobic interactions occurred via the hydrophobic distal moieties. The impact of the substitution of sorafenib azine moiety by the thiazolidine-2,4-dione scaffold of the prepared derivatives on the anticancer actions was also observed. These thiazolidine-2,4-dione moieties inhabited the hydrophobic ATP binding pocket, which was inhabited by the azine moiety of sorafenib and formed H bonding interactions with Cysteine919. Alternatively, various phenyl and aliphatic moieties were established to replace the reference phenyl tail with various electronic and lipophilic natures to examine their action on the antitumor activity. The existence of cyclohexyl tails connected to the sulfonylthiourea linkers and distal benzylidene moieties attached to thiazolidine-2,4-diones, as in derivatives **7c** and **6c**, increased affinities toward the active position, respectively (Figure 12). In addition, the 4-methylbenzylidene derivatives **7a**–**c** displayed greater actions than the unsubstituted derivatives **6a**-**c** and thiazolidine2,4-diones without benzylidene distal moieties **5a**–**c**, respectively. The obtain results showed that the examined derivatives demonstrated various levels of antitumor effects and had a distinguished selectivity model against the HepG2 cell lines. Commonly, the linkers (HBD-HBA), of an electronic and lipophilic nature, displayed a vital role in antitumor activity. From the structure of our compounds and the results presented in Table 3, we can allocate these checked derivatives into three groups. In the first group, containing **5a**–**c**, the thiazolidine 2,4-dione nucleus did not bind to any hydrophobic distal benzylidene moieties. Derivative **5c** and **5b** with a cyclohexyl and phenyl tail, respectively, showed greater actions than derivative **5a** with ethyl ones against the three cell lines (HepG2, HCT116 and MCF-7). In the second group, containing **6a**–**c**, derivative **6c** with a cyclohexyl tail showed greater effects than derivatives **6b** and 6a with phenyl and ethyl tails against both HCT116 and MCF-7. Moreover, in the third group, containing **7a**–**c**, candidate **7c** with a cyclohexyl tail demonstrated higher activities than candidates **7b** and **7a** with phenyl and ethyl tails against both HCT116 and MCF-7.

### 2.8. ADMET, in Silico Studies Profile

An in silico report of the highly active derivatives **6c**, **7b** and **7c** was conducted to evaluate their physicochemical characters and calculate their proposed ADMET profiles. The report was predicted using SwissADME and pkCSM descriptors algorithm procedures [71] and matched to rule of five described by Lipinski [72]. Good absorption properties were expected for the molecules that accomplished at least three rules: (i) hydrogen bond donors are not more than five; (ii) partition coefficient (logP) is not more than 5, (iii) molecular weight less than 500, (iv) hydrogen bond acceptors are not more than 10. In the current work, the standard anticancer agent sorafenib and our new compounds **6c**, **7b** and **7c** violate only one rule. In addition, the ADMET profiles of the three new compounds were initially calculated for their potential evaluation as good drugs.

Considering the obtained data (Table 5), we can assume that these three compounds have good GIT absorption in humans (74.229–76.168), which indicates that they can easily cross different biological membranes [73]. Therefore, they may show a significant high bioavailability through GIT. Concerning CNS penetrability, our prepared compounds have the capability to reach CNS (CNS permeability values −2.254 to −2.529). The CNS permeability values were lower than sorafenib (CNS permeability −2.007) but higher than rosiglitazone (CNS permeability −2.72).

It well known that CYP3A4, the major drug-metabolizing enzyme, can be inhibited by sorafenib and **7b** but **6c**, **7c** and rosiglitazone cannot be. This can be attributed to the superior lipophilicity of sorafenib and **7b**. Elimination was expected depending on the total clearance, which is a considerable factor in deciding dose intervals. The data showed that rosiglitazone confirmed higher clearance rates compared to sorafenib and our new compounds, which demonstrated very low clearance values. Thus, rosiglitazone could be eliminated more quickly. As a result, rosiglitazone is supposed to have shorter dosing intervals. Unlike rosiglitazone, the prepared compounds exhibited a slow clearance rate, which signifies a longer duration of action and extended dosing intervals. Toxicity is the final studied ADMET profile factor. As presented in Table 5, sorafenib, rosiglitazone and the novel compounds shared the drawback of unwanted hepatotoxic actions. Rosiglitazone, **6c** and **7b** demonstrated the lowest maximum tolerated doses. In contrast, sorafenib and **7b** demonstrated the highest maximum tolerated doses, which involve the advantage of the broad therapeutic index of sorafenib and **7b**, respectively. The oral acute toxic dose (LD50) of the novel compound is the least one (2.306), while **6c** and **7c** showed higher oral acute toxic doses than sorafenib but lower than rosiglitazone. Lastly, compared to rosiglitazone, the low-down Minnow toxicity results of the new compounds **6c**, **7c** and sorafenib indicate their excellent selectivity toward tumor cells over normal cells.

## 3. Materials and Methods

### 3.1. Chemistry

#### 3.1.1. General

The starting and intermediate derivatives, thiazolidine-2,4-dione (**1**), 5-benzylidenethiazolidine-2,4-dione (**2a,b**) [22,24] and 2-chloro-*N*-(4-sulfamoylphenyl)acetamide [63], were synthesized according to the reported procedures.

All compounds were crystallized from ethanol, and their NMR spectra were obtained in DMSO-d_6_ solvent at 400 MHz for ^1^HNMR and 100 MHz for ^13^CNMR. NMR and mass data are provided in the Supplementary Materials.

#### 3.1.2. General Procedure for Synthesis of 2-(2,4-Dioxothiazolidin-3-yl)-N-(4-Sulfamoylphenyl)-Acetamide (**3**)

Equimolar quantities of thiazolidine-2,4-dione **1** (1.17 g, 0.01 mol), 2-chloro-*N*-(4-sulfamoylphenyl)acetamide (2.34 g, 0.01 mol) and potassium carbonate (1.5 g, 0.011 mol) in acetone (50 mL) were heated under reflux for 15 h. The reaction mixture was filtered while hot. The filtrate was allowed to cool to room temperature to obtain compound **3**. 

Yield, 82%; m.p. 217–9 °C; IR_νmax_ (cm^−1^): 3402, 3294 (NH & NH_2_), 3062 (CH aromatic), 2958 (CH aliphatic), 1701, 1666 (3C = O amide), 1311, 1161 (SO_2_); ^1^HNMR δ (ppm): 4.22 (s, 2H, NCH_2_), 4.72 (s, 2H, SCH_2_), 7.29 (s, 2H, NH_2_) (D_2_O exchangeable), 7.41–7.93 (m, 4H, aromatic ring) and 10.94 (s, 1H, NH) (D_2_O exchangeable); ^13^CNMR: 23.12, 46.27, 120.98 (2), 130.35 (2), 130.72, 153.44, 154.80, 156.38, 168.06; MS (*m*/*z*): 330 (M^+^ + 1, 15.18%), 329 (M^+^, 9.41%), 271 (26.92%), 131 (100%, base beak), 129 (55.67); Anal. Calcd. for C_11_H_11_N_3_O_5_S_2_ (329.0): C, 40.12; H, 3.37; N, 12.76. Found: C, 40.11; H, 3.25; N, 12.90.

#### 3.1.3. General Procedure for Synthesis of Compounds (**4a,b**)

A mixture of 5-benzylidenethiazolidine-2,4-dione **2a**,**b** (0.01 mol), 2-chloro-*N*-(4-sulfamoylphenyl)acetamide (2.34 g, 0.01 mol) and potassium carbonate (1.5 g, 0.011 mol) in acetone (50 mL) was heated under reflux for 15 h. Then, it was filtered while hot. The filtrate was allowed to cool to room temperature to obtain the target compound **4a**,**b**, respectively. 

##### 2-(5-Benzylidene-2,4-dioxothiazolidin-3-yl)-*N*-(4-sulfamoylphenyl)acetamide (**4a**)

Yield, 85%; m.p. 226–8 °C; IR_νmax_ (cm^−1^): 3418, 3332 (NH & NH_2_), 3036 (CH aromatic), 2958 (CH aliphatic), 1705, 1662 (3C=O amide), 1338, 1180 (SO_2_); ^1^H NMR δ (ppm): 3.98 (s, 2H, CH_2_), 7.25 (s, 2H, NH_2_) (D_2_O exchangeable), 7.62–7.65 (m, 5H, aromatic ring), 7.71–7.74 (m, 4H, aromatic ring), 7.93 (s, 1H, CH=C) and 10.40 (s, 1H, NH) (D_2_O exchangeable); Anal. Calcd. for C_18_H_15_N_3_O_5_S_2_ (417.0): C, 51.79; H, 3.62; N, 10.07. Found: C, 51.85; H, 3.74; N, 10.26.

##### 2-(5-(4-Methylbenzylidene)-2,4-dioxothiazolidin-3-yl)-*N*-(4-sulfamoylphenyl)acetamide (**4b**)

Yield, 85%; m.p. 240–2 °C; IR_νmax_ (cm^−1^): 3425, 3232 (NH & NH_2_), 3055 (CH aromatic), 2962 (CH aliphatic), 1685, 1670, 1651 (3 C=O amide), 1307, 1149 (SO_2_); ^1^H NMR (400 MHz, DMSO-d6) δ (ppm): 2.36 (s, 3H, CH_3_), 4.58 (s, 2H, CH_2_), 7.10–7.66 (m, 10H, 8 aromatic ring & NH_2_ (D_2_O exchangeable)), 7.78 (s, 1H, CH=C) and 10.40 (s, 1H, NH) (D_2_O exchangeable); Anal. Calcd. for C_19_H_17_N_3_O_5_S_2_(431.1): C, 52.89; H, 3.97; N, 9.74. Found: C, 53.12; H, 4.15; N, 9.94.

#### 3.1.4. General Procedure for Syntheses of Compounds (**5a–c**)

Equimolar quantities of the benzenesulfonamide **3** (3.29 g, 0.01 mol), the appropriate isothiocyanate, namely ethylisothiocyanate, phenylisothiocyanate and/or cyclohexylisothiocyanate (0.01 mol) and potassium carbonate (2.5 g, 0.018 mol) in acetone (50 mL), were heated for 24 h under reflux. The reaction mixture was filtered to obtain the target sulfonylthioureas **5a**–**c**, respectively.

##### 2-(2,4-Dioxothiazolidin-3-yl)-*N*-(4-(*N*-(ethylcarbamothioyl)sulfamoyl)phenyl)acetamide (**5a**)

Yield, 78%; m.p. 251–3 °C; IR_νmax_ (cm^−1^): 3348, 3313, 3232 (3NH), 3059 (CH aromatic), 2962 (CH aliphatic), 1701, 1660, 1643 (3C=O), 1315, 1149 (SO_2_); ^1^H NMR δ (ppm): 1.20-1.24 (t, 3H, **CH_3_**CH_2_, *J* = 7.6 Hz), 3.35 (s, 2H, N-CH_2_), 4.15-4.20 (q, 2H, CH_3_**CH_2_**, *J* = 7.6 Hz), 4.49 (s, 2H, S-CH_2_), 6.65 (s, 2H, 2NH) (D_2_O exchangeable), 7.33-7.35 (d, 2H, aromatic ring, *J* = 8 Hz), 7.50–7.52 (d, 2H, aromatic ring, *J* = 8 Hz), and 10.43 (s, 1H, NH)(D_2_O exchangeable); ^13^CNMR: 14.33, 21.51, 42.56, 62.21, 119.61, 130.30, 130.48 (2), 130.75 (2), 134.65, 141.92, 165.44, 167.19, 167.41;MS (*m*/*z*): 417 (M^+^, 19.25%), 338 (100 %, base beak), 294 (86.01%), 130 (57.67%); Anal. Calcd. for C_14_H_16_N_4_O_5_S_3_ (416.5): C, 40.37; H, 3.87; N, 13.45. Found: C, 40.25; H, 4.04; N, 13.62.

##### 2-(2,4-Dioxothiazolidin-3-yl)-*N*-(4-(*N*-(phenylcarbamothioyl)sulfamoyl)phenyl)acetamide (**5b**)

Yield, 70%; m.p. 262–4 °C; IR_νmax_ (cm^−1^): 3317, 3244 (3NH), 3059 (CH aromatic), 2951 (CH aliphatic), 1708, 1662 (3C=O), 1307, 1149 (SO_2_); ^1^H NMR δ(ppm): 4.02 (s, 4H, 2CH_2_), 6.47–7.90 (m, 11H, 9 aromatic ring & 2NH (D_2_O exchangeable)), and 10.81 (s, 1H, NH) (D_2_O exchangeable); Anal. Calcd. for C_18_H_16_N_4_O_5_S_3_ (464.0): C, 46.54; H, 3.47; N, 12.06. Found: C, 46.73; H, 3.55; N, 12.12.

##### *N*-(4-(*N*-(Cyclohexylcarbamothioyl)sulfamoyl)phenyl)-2-(2,4-dioxothiazolidin-3-yl)acetamide (**5c**)

Yield, 84%; m.p. 267–9 °C; IR_νmax_ (cm^−1^): 3290, 3194 (3NH), 3043 (CH aromatic), 2958 (CH aliphatic), 1705, 1658 (3C=O), 1308, 1149 (SO_2_); ^1^H NMR (400 MHz, DMSO-d6) δ (ppm): 1.02–1.85 (m, 10H, cyclohexyl), 3.40–3.45 (m, 1H, cyclohexyl), 4.05 (s, 2H, N-CH_2_), 4.47 (s, 2H, S-CH_2_), 6.41 (s, 2H, 2NH) (D_2_O exchangeable), 7.03–7.12 (m, 4H, aromatic ring), and 10.08 (s, 1H, NH) (D_2_O exchangeable); MS (*m/z*): 470 (M^+^, 30.11 %), 455 (32.58%), 335 (76.18%), 292 (100%, base beak), 259 (82.11%), 160 (48.17%); Anal. Calcd. for C_18_H_22_N_4_O_5_S_3_ (470.1): C, 45.94; H, 4.71; N, 11.91. Found: C, 46.13; H, 4.80; N, 11.75.

##### 3.1.5. General Procedure for Syntheses of Compounds (**6a–c**)

Equimolar quantities of the benzylidenesulfonamide **4a** (4.17 g, 0.01 mol), the appropriate isothiocyanate, namely ethylisothiocyanate, phenylisothiocyanate and/or cyclohexylisothiocyanate (0.01 mol) and potassium carbonate (2.5 g, 0.018 mol) in acetone (50 mL), were heated for 24 h under reflux. The reaction mixture was filtered to obtain the target sulfonylthioureas **6a**–**c**, respectively.

###### 2-(5-Benzylidene-2,4-dioxothiazolidin-3-yl)-*N*-(4-(*N*-(ethylcarbamothioyl)sulfamoyl)phenyl)acetamide (**6a**)

Yield, 85%; m.p. 282–4 °C; IR_νmax_ (cm^−1^): 3329, 3259 (3NH), 3047 (CH aromatic), 2951 (CH aliphatic), 1708, 1666 (3C=O), 1346, 1145 (SO_2_); ^1^H NMR δ (ppm): 0.87-0.91 (t, 3H, CH_3_, *J* = 8.8 Hz), 3.18–3.23 (q, 2H, CH_2_, *J* = 8.8 Hz), 4.56 (s, 2H, N-CH_2_), 7.36-8.39 (m, 11H, 9 aromatic ring & 2NH (D_2_O exchangeable)), 8.56 (s, 1H, CH = C) and 10.68 (s, 1H, NH) (D_2_O exchangeable); ^13^CNMR: 13.39, 21.75 (2), 56.59, 114.56 (2), 116.61, 118.78, 121.76, 123.99, 124.33, 125.58, 128.90, 129.26, 129.32 (2), 129.50 (2), 148.60, 158.33, 171.26; MS (*m/z*): 505 (M^+^, 38.08%), 502 (43.68%), 462 (66.48%), 338 (96.56%), 246 (100%, base beak), 102 (70.03 %); Anal. Calcd. for C_21_H_20_N_4_O_5_S_3_ (504.6): C, 49.99; H, 4.00; N, 11.10. Found: C, 49.85; H, 4.03; N, 11.05.

###### 2-(5-Benzylidene-2,4-dioxothiazolidin-3-yl)-*N*-(4-(*N*-(phenylcarbamothioyl)sulfamoyl)phenyl)-acetamide (**6b**)

Yield, 75%; m.p. 286–8 °C; IR_νmax_ (cm^−1^): 3398, 3298 (3NH), 3059 (CH aromatic), 2954 (CH aliphatic), 1708, 1680, 1666 (3C = O), 1320, 1157 (SO_2_); ^1^H NMR δ (ppm): 4.95 (s, 2H, CH_2_), 6.46–7.90 (m, 15H, 14 aromatic ring & NH (D_2_O exchangeable)), 9.99 (s, H, NH) (D_2_O exchangeable) and 10.75 (s, H, NH) (D_2_O exchangeable); MS (*m*/*z*): 554 (M^+^, 20.05%), 552 (27.72%), 496 (73.46%), 381 (80.88%), 300 (100%, base beak), 208 (67.35%); Anal. Calcd. for C_25_H_20_N_4_O_5_S_3_ (552.1): C, 54.33; H, 3.65; N, 10.14. Found: C, 54.71; H, 3.70; N, 10.34.

###### 2-(5-Benzylidene-2,4-dioxothiazolidin-3-yl)-*N*-(4-(*N*-(cyclohexylcarbamothioyl)sulfamoyl)phenyl)-acetamide (**6c**)

Yield, 75%; m.p. 294–6 °C; IR_νmax_ (cm^−1^): 3398, 3332, 3275 (3NH), 3059 (CH aromatic), 2924 (CH aliphatic), 1693 (3 C=O), 1315, 1149 (SO_2_); ^1^H NMR δ (ppm): 1.24–1.93 (m, 10H, cyclohexyl), 3.57–3.63 (m, 1H, cyclohexyl), 4.09 (s, 2H, CH_2_), 6.02 (s, 2H, 2NH) (D_2_O exchangeable), 7.24–7.99 (m, 9H, aromatic ring), 8.42 (s, 1H, CH=C) and 10.43 (s, 1H, NH) (D_2_O exchangeable); ^13^CNMR: 22.87, 24.86, 31.35, 32.78 (2), 46.55 (2), 55.43, 126.4 (4), 127.42 (5), 135.45, 143.09, 156.60 (2), 171.60 (2), 195.20; Anal. Calcd. for C_25_H_26_N_4_O_5_S_3_ (558.1): C, 53.75; H, 4.69; N, 10.03. Found: C, 53.75; H, 4.69; N, 10.03.

##### 3.1.6. General Procedure for Synthesis of Compounds (**7a–c**)

Equimolar quantities of the 4-methylbenzylidenesulfonamide **4b** (4.31 g, 0.01 mol), the appropriate isothiocyanate, namely ethylisothiocyanate, phenylisothiocyanate and/or cyclohexylisothiocyanate (0.01 mol) and potassium carbonate (2.5 g, 0.018 mol) in acetone (50 mL), were heated for 24 h under reflux. The reaction mixture was filtered to obtain sulfonylthiourea derivatives **7a**–**c**, respectively.

###### *N*-(4-(*N*-(Ethylcarbamothioyl)sulfamoyl)phenyl)-2-(5-(4-methylbenzylidene)-2,4-dioxothiazolidin-3-yl)acetamide (**7a**)

Yield, 85%; m.p. 285–7 °C; IR_νmax_ (cm^−1^): 3336, 3201 (3NH), 3066 (CH aromatic), 2931 (CH aliphatic), 1697 (3C=O), 1315, 1176 (SO_2_); ^1^H NMR δ (ppm): 0.87–0.93 (t, 3H, **CH_3_**-CH_2_), *J* = 5.6 Hz), 1.71 (s, 3H, CH_3_-phenyl), 3.09 (q, 2H, CH_3_-**CH_2_**, *J* = 5.6 Hz), 3.87 (s, 2H, N-CH_2_), 6.26, 6.62 (s, 2H, 2NH) (D_2_O exchangeable), 7.41-7.71 (m, 8H, aromatic ring), 7.79 (s, 1H, CH=C) and 10.26 (s, 1H, NH) (D_2_O exchangeable); MS (*m/z*): 519 (M^+^, 36.00%), 517 (2.25%), 445 (100%, base beak), 420 (76.07%), 250 (72.49%), 191 (45.90%); Anal. Calcd. for C_22_H_22_N_4_O_5_S_3_ (518.6): C, 50.95; H, 4.28; N, 10.80. Found: C, 50.84; H, 4.36; N, 10.87.

###### 2-(5-(4-Methylbenzylidene)-2,4-dioxothiazolidin-3-yl)-*N*-(4-(*N*-(phenylcarbamothioyl)sulfamoyl)-phenyl)acetamide (**7b**)

Yield, 73%; m.p. 290–2 °C; IR_νmax_ (cm^−1^): 3298, 3190 (3NH), 3070 (CH aromatic), 2920 (CH aliphatic), 1690, 1681, 1647 (3C=O), 1323, 1153 (SO_2_); ^1^H NMR δ (ppm): 2.34 (s, 3H, CH_3_), 3.97 (s, 2H, CH_2_), 6.47-7.93 (m, 15H, 13 aromatic ring & CH=C & NH (D_2_O exchangeable)), 10.00 (s, 1H, 1NH) (D_2_O exchangeable) and 10.76 (s, 1H, NH) (D_2_O exchangeable); ^13^CNMR: 13.38, 56.59, 114.69 (4), 116.80 (2), 121.78 (2), 122.41, 123.56, 124.03, 124.47, 125.59, 128.93 (4), 129.35 (4), 129.50, 148.48, 169.41; MS (*m*/*z*): 567 (M^+^, 11.57%), 493 (92.95%), 332 (100%, base beak), 282 (46.61%), 176 (45.13%), 79 (54.01%); Anal. Calcd. for C_26_H_22_N_4_O_5_S_3_ (566.7): C, 55.11; H, 3.91; N, 9.89. Found: C, 55.17; H, 3.98; N, 9.78.

###### *N*-(4-(*N*-(Cyclohexylcarbamothioyl)sulfamoyl)phenyl)-2-(5-(4-methylbenzylidene)-2,4-dioxothiazolidin-3-yl)acetamide (**7c**)

Yield, 83%; m.p. 300–2 °C; IR_νmax_ (cm^−1^): 3414, 3232,3116 (3NH), 3078 (CH aromatic), 2927 (CH aliphatic), 1710, 1697, 1640 (3C=O), 1315, 1149 (SO_2_); ^1^H NMR δ (ppm): 1.06–1.84 (m, 10H, cyclohexyl), 2.52 (s, 3H, CH_3_), 3.69 (m, 1H, cyclohexyl), 4.30 (s, 2H, CH_2_), 5.95 (s, 2H, 2NH) (D_2_O exchangeable). 7.28–8.04 (m, 8H, aromatic ring), 8.39 (s, 1H, CH=C) and 10.68 (s, 1H, NH) (D_2_O exchangeable); ^13^CNMR: 22.70, 32.75 (2), 43.66 (2), 43.70, 55.42, 61.90, 113.69, 119.53, 119.81, 119.97, 121.14, 126.77, 127.31, 127.89, 129.92, 130.57, 132.98, 134.52, 138.97, 141.50, 141.62, 165.87, 166.18, 168.00; MS (*m/z*): 573 (M^+^, 24.94%), 555 (29.22%), 532 (59.00%), 483 (100%, base beak), 419 (93.22%), 304 (43.73%), 134 (40.81%); Anal. Calcd. for C_26_H_28_N_4_O_5_S_3_ (572.7): C, 54.53; H, 4.93; N, 9.78. Found: C, 54.60; H, 4.90; N, 9.75.

## 4. Conclusions

In summary, 12 novel thiazolidine-2,4-dione-based compounds were designed and synthesized, and their anticancer activities were evaluated against HepG2-, HCT-116- and MCF-7-inhibiting VEGFR-2 enzymes. They showed potent activity against the HepG2 cell line compared to the other HCT116 and MCF-7 cell lines. Compounds **7c** and **6c** were highly effective derivatives against the MCF-7 (IC_50_ = 7.78 and 8.15 µM), HCT116 (IC_50_ = 5.77 and 7.11 µM) and HepG2 (IC_50_ = 8.82 and 8.99 µM) tumor cell lines. Their activities were lower than sorafenib (IC_50_ = 7.26, 5.47 and 9.18 µM) against MCF-7 and HCT116 but higher against HepG2, respectively. In addition, the anticancer activities of these compounds were lower than doxorubicin (IC_50_ = 6.75, 8.07 and 7.94 µM) against the three cell lines, respectively. The highly effective derivatives **6a**–**c** and **7a**–**c** were tested against VERO normal cell lines. The results showed that the assessed derivatives showed low toxicity against normal VERO cells, with IC_50_ values ranging from 40.88 to 68.25 µM. All derivatives were evaluated for their VEGFR-2 inhibitory actions and demonstrated high to low activities, with IC_50_ values varying from 0.08 to 0.93 µM. Derivatives **7c** and **6c** were observed to be the most effective compounds, with IC_50_ = 0.08 µM against VEGFR-2. Moreover, derivatives **5a**–**c**, **6a**–**c** and **7a**–**c** were assessed to verify their in vitro binding affinities to PPARγ and insulin-secreting activities. Compounds **7c** and **6c** notably bound to PPARγ, with IC_50_ = 0.296 and 0.300 µM, respectively. In addition, derivatives **7c** and **6c** showed powerful insulin-secreting activities with the same EC_50_ value of 0.70 µM. Moreover, docking studies were performed for binding mode and affinities investigation toward both VEGFR-2 and PPARγ receptors. The docking data were highly associated with that of the biological screening. Furthermore, our compounds represented good in silico ADMET calculations.

## Figures and Tables

**Table 1:** The ligands binding free energy (calculated) (∆G in Kcal/mole).

Compound	∆G [kcal mol^−1^]	Compound	∆G [kcal mol^−1^]
**3**	−90.00	**6b**	−126.72
**4a**	−92.62	**6c**	−130.36
**4b**	−98.37	**7a**	−125.48
**5** **a**	−115.75	**7b**	−129.68
**5b**	−121.80	**7c**	−138.79
**5c**	−125.96	**Sorafenib**	−110.12
**6a**	−118.64		

**Table 2:** The ligands binding free energy (calculated) (∆G in Kcal/mole).

Compound	∆G [kcal mol^−1^]	Compound	∆G [kcal mol^−1^]
**3**	−84.12	**6b**	−118.03
**4a**	−92.97	**6c**	−128.20
**4b**	−91.92	**7a**	−125.77
**5** **a**	−108.58	**7b**	−125.42
**5b**	−111.97	**7c**	−132.78
**5c**	−119.97	**Rosiglitazone**	−94.38
**6a**	−115.47		

**Table 3:** Novel prepared derivatives in vitro cytotoxicity against HepG2, HCT-116, MCF-7 and VERO cell lines, and VEGFR-2 kinase assay.

Compound	IC_50_ (µM) ^a^				
	HepG2	HCT116	MCF-7	VERO	VEGFR-2
**3**	58.55 ± 5.1	61.48 ± 5.1	60.18 ± 5.1	^b^ NT	0.93± 0.06
**4a**	52.87 ± 5.1	55.12 ± 5.1	54.99 ± 5.1	^b^ NT	0.92 ± 0.06
**4b**	48.56 ± 5.1	57.87 ± 5.1	62.43 ± 5.1	^b^ NT	0.89 ± 0.06
**5** **a**	24.49 ± 2.2	40.11 ± 2.2	28.79 ± 2.2	^b^ NT	0.46 ± 0.05
**5b**	21.99 ± 2.0	25.68 ± 2.0	23.24 ± 2.0	^b^ NT	0.44 ± 0.05
**5c**	20.75 ± 2.6	23.56 ± 2.6	24.59 ± 2.6	^b^ NT	0.44 ± 0.05
**6a**	14.16 ± 2.3	17.65 ± 2.3	16.47 ± 2.3	48.31 ± 0.22	0.17 ± 0.02
**6b**	10.67 ± 1.6	13.78 ± 1.2	12.95 ± 1.2	40.88 ± 0.22	0.15 ± 0.02
**6c**	8.99 ± 1.2	7.11 ± 1.7	8.15 ± 1.6	49.26 ± 0.22	0.08 ± 0.02
**7a**	12.05 ± 1.5	16.79 ± 1.5	16.66 ± 1.5	60.12 ± 0.18	0.14 ± 0.02
**7b**	9.65 ± 1.7	13.48 ± 1.6	12.89 ± 1.7	52.61 ± 0.22	0.11 ± 0.02
**7c**	8.82 ± 1.9	5.77 ± 1.9	7.78 ± 1.9	68.25 ± 0.21	0.08 ± 0.02
**Sorafenib**	9.18 ± 0.6	5.47 ± 0.3	7.26 ± 0.3	^b^ NT	0.10 ± 0.02
**Doxorubicin**	7.94 ± 0.6	8.07 ± 0.8	6.75 ± 0.4	^b^ NT	^b^ NT

^a^ IC_50_ = mean ± S.D. of triplet separate experiments. ^b^ NT: Derivatives not assessed as VEGFR-2 inhibitors.

**Table 4:** In vitro PPARγ binding affinities and insulin-secreting activities of the new derivatives and standard drugs.

Comp.	In Vitro ^a^	
	IC_50_ (µM) ^b^ PPARγ Binding Affinity	EC_50_ (µM) ^c^ Insulin Secreting Activity
**5** **a**	0.393	1.20
**5b**	0.377	1.13
**5c**	0.360	1.00
**6a**	0.323	0.87
**6b**	0.308	0.78
**6c**	0.300	0.70
**7a**	0.320	0.81
**7b**	0.305	0.75
**7c**	0.296	0.70
**Rosiglitazone**	0.292	NT ^d^
**Glimiperide**	NT ^d^	0.73

^a^ All results are within 10% (*n* = 3). ^b^ Concentration of the tested derivatives required to displace 50% of the titrated ligand. ^c^ Concentration needed to make 50% of the maximum action.

**Table 5:** The highest effective compounds, rosiglitazone and sorafenib. ADMET profile.

Parameter	6c	7b	7c	Rosiglit.	Sorafenib
**Physicochemical properties**					
Molecular Weight	558.69	566.67	572.72	357.43	464.82
LogP	3.8471	4.34532	4.15552	2.4909	5.5497
Rotatable Bonds	10	10	10	7	9
Acceptors	5	5	5	4	7
Donors	3	3	3	1	3
TPSA	190.45 Å^2^	190.45 Å^2^	190.45 Å^2^	96.83 Å^2^	92.35 Å^2^
**Absorption**					
Water solubility	−4.149	−3.637	−4.204	−3.841 −5.284	−4.822
Caco2 permeability	0.714	0.699	0.623	0.934	0.689
Human Intest. absorption	74.229	76.168	74.687	95.437	89.043
Skin Permeability	−2.738	−2.735	−2.738	95.437	−2.767
Substrate for P-glycoprotein	+	+	+	-	+
Inhibitor of P-glycoprotein I	+	+	+	-	+
Inhibitor of P-glycoprotein II	+	+	+	-	+
**Distribution**					
VDss (human)	−0.192	−0.008	−0.168	−0.264	−0.29
Human unbound fraction	0.07	0.064	0.06	0.188	0.065
Permeability throughout BBB	−1.618	−1.594	−1.648	−0.618	−1.684
Permeability to CNS	−2.529	−2.254	−2.454	−2.72	−2.007
**Metabolism**					
CYP2D6 substrate	-	-	-	-	-
CYP3A4 substrate	+	+	+	+	+
Inhibition of CYP3A4	-	+	-	-	+
Inhibition of CYP2D6	-	-	-	-	-
Inhibition of CYP2C9	-	+	-	-	+
Inhibition of CYP2C19	-	+	-	-	+
Inhibition of CYP1A2	-	+	-	-	+
**Excretion**					
Clearance	−0.538	−0.670	−0.594	0.104	−0.219
Renal OCT2 substrate	-	-	-	-	-
**Toxicity**					
AMES toxicity	-	-	-	-	-
Human Max. tolerated dose	−0.512	0.558	−0.546	−0.460	0.549
Inhibitor of hERG I	-	-	-	-	-
hERG II inhibitor	+	+	+	-	+
Acute Toxic activity (LD_50_)	2.708	2.306	2.773	2.877	2.538
Chronic Toxic activity (LOAEL)	0.826	1.465	0.739	1.313	1.198
Hepatotoxic effect	+	+	+	+	+
Skin Sensitization	-	-	-	-	-
T. Pyriformis toxicity	0.337	0.29	0.337	1.194	0.383
Minnow toxic activity	0.214	1.337	0.543	0.609	0.189

## Data Availability

Data is contained within the article.

## Supplementary Materials

The following supporting information can be downloaded at: https://www.mdpi.com/article/10.3390/ph15020226/s1. Supplementary Material is the NMR and mass data of the compounds.

## References

[1] Yang Y., Hu X., Zhang Q., Zou R. (2016). Diabetes mellitus and risk of fall in older adult: A systematic review and meta-analysis. Age Ageing.

[2] Yadav U., Vanjari Y., Laxmikeshav K., Tokala R., Niggula P.K., Kumar M., Talla V., Kamal A., Shankaraiah N. (2021). Synthesis and in Vitro Cytotoxicity Evaluation of Phenanthrene Linked 2,4-Thiazolidinediones as Potential Anticancer Agents. Anti-Cancer Agents Med. Chem..

[3] Tahlan S.S., Verma P.K. (2017). Biological potential of thiazolidinedione derivatives of synthetic origin. Chem. Cent. J..

[4] Hussein A.H., Moghimi A., Roohbakhsh A. (2019). Anticonvulsant and ameliorative effects of pioglitazone on cognitive deficits, inflammation and apoptosis in the hippocampus of rat pups exposed to febrile seizure. Iran. J. Basic Med. Sci..

[5] Sutinen J. (2009). The Effects of Thiazolidinediones on Metabolic Complications and Lipodystrophy in HIV-Infected Patients. PPAR Res..

[6] Rekha S., Shantharam U., Vineet C. (2011). Synthesis and evaluation of novel thiazolidinedione for anti-inflammatory activity. Int. Res. J. Pharm..

[7] Sameeh M.Y., Khowdiary M.M., Nassar H.S., Abdelall M.M., Amer H.H., Hamed A., Elhenawy A.A. (2022). Thiazolidinedione Derivatives: In Silico, In Vitro, In Vivo, Antioxidant and Anti-Diabetic Evaluation. Molecules.

[8] Panigrahy D., Singer S., Shen L.Q., Butterfield C.E., Freedman D.A., Chen E.J., Moses M.A., Kilroy S., Duensing S., Fletcher C. (2002). PPARγ ligands inhibit primary tumor growth and metastasis by inhibiting angiogenesis. J. Clin. Investig..

[9] Joshi H., Pal T., Ramaa C. (2014). A new dawn for the use of thiazolidinediones in cancer therapy. Expert Opin. Investig. Drugs.

[10] Bhanushali U., Rajendran S., Sarma K., Kulkarni P., Chatti K., Chatterjee S., Ramaa C. (2016). 5-Benzylidene-2, 4-thiazolidenedione derivatives: Design, synthesis and evaluation as inhibitors of angiogenesis targeting VEGR-2. Bioorg. Chem..

[11] Rashid M., Shrivastava N., Husain A. (2020). Synthesis and sar strategy of thiazolidinedione: A novel approach for cancer treatment. J. Chil. Chem. Soc..

[12] Han S.W., Roman J. (2010). Anticancer actions of PPARγ ligands: Current state and future perspectives in human lung cancer. World J. Biol. Chem..

[13] Bo Q.F., Sun X.M., Liu J., Sui X.M., Li G.X. (2015). Antitumor action of the peroxisome proliferator-activated receptor-γ agonist rosiglitazone in hepatocellular carcinoma. Oncol. Lett..

[14] Blanquicett C., Roman J., Hart C.M. (2008). Thiazolidinediones as anti-cancer agents. Cancer Ther..

[15] Yoshizaki T., Motomura W., Tanno S., Kumei S., Yoshizaki Y., Tanno S., Okumura T. (2010). Thiazolidinediones enhance vascular endothelial growth factor expression and induce cell growth inhibition in non-small-cell lung cancer cells. J. Exp. Clin. Cancer Res..

[16] Corigliano D.M., Syed R., Messineo S., Lupia A., Patel R., Reddy C.V.R., Dubey P.K., Colica C., Amato R., De Sarro G. (2018). Indole and 2,4-Thiazolidinedione conjugates as potential anticancer modulators. PeerJ.

[17] Ha Y.M., Park Y.J., Kim J.A., Park D., Park J.Y., Lee H.J., Lee J.Y., Moon H.R., Chung H.Y. (2012). Design and synthesis of 5-(substituted benzylidene) thiazolidine-2, 4-dione derivatives as novel tyrosinase inhibitors. Eur. J. Med. Chem..

[18] Li Q., Al-Ayoubi A., Guo T., Zheng H., Sarkar A., Nguyen T., Eblen S.T., Grant S., Kellogg G.E., Zhang S. (2009). Structure–activity relationship (SAR) studies of 3-(2-amino-ethyl)-5-(4-ethoxy-benzylidene)-thiazolidine-2, 4-dione: Development of potential substrate-specific ERK1/2 inhibitors. Bioorg. Med. Chem. Lett..

[19] Nastasă C., Tamaian R., Oniga O., Tiperciuc B. (2019). 5-Arylidene (chromenyl-methylene)-thiazolidinediones: Potential New Agents against Mutant Oncoproteins K-Ras, N-Ras and B-Raf in Colorectal Cancer and Melanoma. Medicina.

[20] Shah D.K., Menon K.M.J., Cabrera L.M., Vahratian A., Kavoussi S.K., Lebovic D.I. (2010). Thiazolidinediones decrease vascular endothelial growth factor (VEGF) production by human luteinized granulosa cells in vitro. Fertil. Steril..

[21] El-Adl K., El-Helby A.-G.A., Sakr H., Eissa I.H., El-Hddad S.S., Shoman F.M. (2020). Design, synthesis, molecular docking and anticancer evaluations of 5-benzylidenethiazolidine-2,4-dione derivatives targeting VEGFR-2 enzyme. Bioorg. Chem..

[22] El-Adl K., Sakr H., El-Hddad S.S.A., El-Helby A.A., Nasser M., Abulkhair H.S. (2021). Design, synthesis, docking, ADMET profile, and anticancer evaluations of novel thiazolidine-2,4-dione derivatives as VEGFR-2 inhibitors. Arch. Pharm..

[23] El-Adl K., Sakr H., Nasser M., Alswah M., Shoman F.M.A. (2020). 5-(4-Methoxybenzylidene)thiazolidine-2, 4-dione-derived VEGFR-2 inhibitors: Design, synthesis, molecular docking, and anticancer evaluations. Arch. Pharm..

[24] El-Adl K., El-Helby A.A., Sakr H., Ayyad R.R., Mahdy H.A., Nasser M., Abulkhair H.S., El-Hddad S.S.A. (2020). Design, synthesis, molecular docking, anticancer evaluations, and in silico pharmacokinetic studies of novel 5-[(4-chloro/2,4-dichloro)benzylidene]thiazolidine-2,4-dione derivatives as VEGFR-2 inhibitors. Arch. Pharm..

[25] Takahashi S. (2011). Vascular endothelial growth factor (VEGF), VEGF receptors and their inhibitors for antiangiogenic tumor therapy. Biol. Pharm. Bull..

[26] Kankanala J., Latham A., Johnson A., Homer-Vanniasinkam S., Fishwick C., Ponnambalam S. (2012). A combinatorial in silico and cellular approach to identify a new class of compounds that target VEGFR2 receptor tyrosine kinase activity and angiogenesis. Br. J. Pharmacol..

[27] Wu P., Nielsen T.E., Clausen M.H. (2015). FDA-approved small-molecule kinase inhibitors. Trends Pharmacol. Sci..

[28] Wilhelm S., Carter C., Lynch M., Lowinger T., Dumas J., Smith R.A., Schwartz B., Simantov R., Kelley S. (2006). Discovery and development of sorafenib: A multikinase inhibitor for treating cancer. Nat. Rev. Drug Discov..

[29] Pircher A., Hilbe W., Heidegger I., Drevs J., Tichelli A., Medinger M. (2011). Biomarkers in tumor angiogenesis and anti-angiogenic therapy. Int. J. Mol. Sci..

[30] Herbst K.J., Coltharp C., Amzel L.M., Zhang J. (2011). Direct activation of Epac by sulfonylurea is isoform selective. Chem. Biol..

[31] Faidallah H.M., Al-Mohammadi M.M., Alamry K.A., Khan K.A. (2016). Synthesis and biological evaluation of fluoropyrazolesulfonylurea and thiourea derivatives as possible antidiabetic agents. J. Enzym. Inhib. Med. Chem..

[32] Vila-Carriles W.H., Zhao G., Bryan J. (2007). Defining a binding pocket for sulfonylureas in ATP-sensitive potassium channels. FASEB J..

[33] Fukuen S., Iwaki M., Yasui A., Makishima M., Matsuda M., Shimomura I. (2015). Sulfonylurea agents exhibit peroxisome proliferator-activated receptor γ agonistic activity. J. Biol. Chem..

[34] Inukai K., Watanabe M., Nakashima Y., Takata N., Isoyama A., Sawa T., Kurihara S., Awata T., Katayama S. (2005). Glimepiride enhances intrinsic peroxisome proliferator-activated receptor-γ activity in 3T3-L1 adipocytes. Biochem. Biophys. Res. Commun..

[35] Arrault A., Rocchi S., Picard F., Maurois P., Pirotte B., Vamecq J. (2009). A short series of antidiabetic sulfonylureas exhibit multiple ligand PPARγ-binding patterns. Biomed. Pharmacother..

[36] El-Adl K., El-Helby A.-G.A., Sakr H., Elwan A. (2021). Design, synthesis, molecular docking and anti-proliferative evaluations of [1,2,4]Triazolo[4,3-a]quinoxaline and [1,2,4]triazolo[4,3-a]quinoxaline-1-thiol derived DNA intercalators: Design, synthesis, molecular docking, in silico ADMET profile and antiproliferative evaluations. New J. Chem..

[37] Eissa I.H., Metwaly A.M., Belal A., Mehany A.B., Ayyad R.R., El-Adl K., Mahdy H.A., Taghour M.S., El-Gamal K.M., El-Sawah M.E. (2019). Discovery and anti-proliferative evaluation of new quinoxalines as potential DNA intercalators and topoisomerase II inhibitors. Arch. Pharm..

[38] El-Adl K., Ibrahim M.K., Alesawy M.S.I., Eissa I.H. (2021). [1,2,4]Triazolo[4,3-c]quinazoline and bis([1,2,4]triazolo)[4,3-a:4’,3’-c]quinazoline derived DNA intercalators: Design, synthesis, in silico ADMET profile, molecular docking and anti-proliferative evaluation studies. Bioorg. Med. Chem..

[39] El-Adl K., El-Helby A.-G.A., Sakr H., Elwan A. (2020). Design, synthesis, molecular docking and anti-proliferative evaluations of [1,2,4]triazolo[4,3-a]quinoxaline derivatives as DNA intercalators and Topoisomerase II inhibitors. Bioorg. Chem..

[40] Alesawy M.S., Ibrahim M.-K., Eissa I.H., El-Adl K. (2022). Design, synthesis, in silico ADMET, docking, and antiproliferative evaluations of [1,2,4]triazolo[4,3-c]quinazolines as classical DNA intercalators. Arch. Pharm..

[41] El-Zahabi M.A., Sakr H., El-Adl K., Zayed M., Abdelraheem A.S., Eissa S.I., Elkady H., Eissa I.H. (2020). Design, synthesis, and biological evaluation of new challenging thalidomide analogs as potential anticancer immunomodulatory agents. Bioorg. Chem..

[42] Ran F., Li W., Qin Y., Yu T., Liu Z., Zhou M., Liu C., Qiao T., Li X., Yousef R.G. (2021). Inhibition of Vascular Smooth Muscle and Cancer Cell Proliferation by New VEGFR Inhibitors and Their Immunomodulator Effect: Design, Synthesis, and Biological Evaluation. Oxidative Med. Cell. Longev..

[43] El-Helby A.A., Sakr H., Ayyad R.R., Mahdy H.A., Khalifa M.M., Belal A., Rashed M., El-Sharkawy A., Metwaly A.M., Elhendawy M.A. (2020). Design, synthesis, molecular modeling, in vivo studies and anticancer activity evaluation of new phthalazine derivatives as potential DNA intercalators and topoisomerase II inhibitors. Bioorg. Chem..

[44] Sakr H., Ayyad R.R., El-Helby A.A., Khalifa M.M., Mahdy H.A. (2021). Discovery of novel triazolophthalazine derivatives as DNA intercalators and topoisomerase II inhibitors. Arch. Pharm..

[45] Eissa I.H., El-Helby A.A., Mahdy H.A., Khalifaa M.M., Elnagar H.A., Mehany A.B.M., Metwaly A.M., Elhendawy M.A., Radwan M.M., ElSohly M.A. (2020). Discovery of new quinazolin-4(3*H*)-ones as VEGFR-2 inhibitors: Design, synthesis, and anti-proliferative evaluation. Bioorg. Chem..

[46] Saleh N.M., El-Gaby M.S.A., El-Adl K., El-Sattar N.E.A.A. (2020). Design, green synthesis, molecular docking and anticancer evaluations of diazepam bearing sulfonamide moieties as VEGFR-2 inhibitors. Bioorg. Chem..

[47] El-Adl K., El-Helby A.A., Ayyad R.R., Mahdy H.A., Khalifa M.M., Elnagar H.A., Mehany A.B.M., Metwaly A.M., Elhendawy M.A., Radwan M.M. (2020). Design, synthesis, and anti-proliferative evaluation of new quinazolin-4(3*H*)-ones as potential VEGFR-2 inhibitors. Bioorg. Med. Chem..

[48] El-Helby A.A., Sakr H., Eissa I.H., Al-Karmalawy A.A., El-Adl K. (2019). Benzoxazole/benzothiazole-derived VEGFR-2 inhibitors: Design, synthesis, molecular docking, and anticancer evaluations. Arch. Pharm..

[49] El-Helby A.-G.A., Ayyad R.R.A., Sakr H., El-Adl K., Ali M.M., Khedr F. (2017). Design, Synthesis, Molecular Docking, and Anticancer Activity of Phthalazine Derivatives as VEGFR-2 Inhibitors. Arch. Pharm..

[50] El-Helby A.-G.A., Ayyad R.R.A., Sakr H., El-Adl K., Ali M.M., Khedr F. (2018). Design, Synthesis, In Vitro Anti-cancer Activity, ADMET Profile and Molecular Docking of Novel Triazolo[3,4-a]phthalazine Derivatives Targeting VEGFR-2 Enzyme. Anti-cancer Agents Med. Chem..

[51] El-Helby A.A., Sakr H., Eissa I.H., Abulkhair H., Al-Karmalawy A.A., El-Adl K. (2019). Design, synthesis, molecular docking, and anticancer activity of benzoxazole derivatives as VEGFR-2 inhibitors. Arch. Pharm..

[52] Turky A., Bayoumi A.H., Sherbiny F.F., El-Adl K., Abulkhair H.S. (2021). Unravelling the anticancer potency of 1,2,4-triazole-N-arylamide hybrids through inhibition of STAT3: Synthesis and in silico mechanistic studies. Mol. Divers..

[53] El-Helby A.G.A., Ayyad R.R., Sakr H.M., Abdelrahim A.S., El-Adl K., Sherbiny F.S., Eissa I.H., Khalifa M.M. (2017). Design, synthesis, molecular modeling and biological evaluation of novel 2,3-dihydrophthalazine-1,4-dione derivatives as potential anticonvulsant agents. J. Mol. Struct..

[54] El-Shershaby M.H., El-Gamal K.M., Bayoumi A.H., El-Adl K., Ahmed H.E.A., Abulkhair H.S. (2021). Synthesis, antimicrobial evaluation, DNA gyrase inhibition, and in silico pharmacokinetic studies of novel quinoline derivatives. Arch. Pharm..

[55] Lee K., Jeong K.-W., Lee Y., Song J.Y., Kim M.S., Lee G.S., Kim Y. (2010). Pharmacophore modeling and virtual screening studies for new VEGFR-2 kinase inhibitors. Eur. J. Med. Chem..

[56] Machado V.A., Peixoto D., Costa R., Froufe H.J., Calhelha R.C., Abreu R.M., Ferreira I.C., Soares R., Queiroz M.-J.R. (2015). Synthesis, antiangiogenesis evaluation and molecular docking studies of 1-aryl-3-[(thieno[3,2-b] pyridin-7-yl- thio)phenyl]ureas: Discovery of a new substitution pattern for type II VEGFR-2 Tyr kinase inhibitors. Bioorg. Med. Chem..

[57] Dietrich J., Hulme C., Hurley L.H. (2010). The design, synthesis, and evaluation of 8 hybrid DFG-out allosteric kinase inhibitors: A structural analysis of the binding interactions of Gleevec^®^, Nexavar^®^, and BIRB-796. Bioorg. Med. Chem..

[58] Garofalo A., Goossens L., Six P., Lemoine A., Ravez S., Farce A., Depreux P. (2011). Impact of aryloxy-linked quinazolines: A novel series of selective VEGFR-2 receptor tyrosine kinase inhibitors. Bioorg. Med. Chem. Lett..

[59] Aziz M.A., Serya R.A., Lasheen D.S., Abdel-Aziz A.K., Esmat A., Mansour A.M., Singab A.N.B., Abouzid K.A. (2016). Discovery of Potent VEGFR-2 Inhibitors based on Furopyrimidine and Thienopyrimidne Scaffolds as Cancer Targeting Agents. Sci. Rep..

[60] Zoete V., Grosdidier A., Michielin O. (2007). Peroxisome proliferator-activated receptor structures: Ligand specificity, molecular switch and interactions with regulators. Biochim. Biophys. Acta (BBA)-Mol. Cell Biol. Lipids.

[61] Groop L., Neugebauer G., Kuhlmann J., Puls W. (1996). Clinical Pharmacology of Sulfonylureas. Oral Antidiabetics.

[62] Ibrahim M.-K., Eissa I.H., Alesawy M.S., Metwaly A.M., Radwan M.M., ElSohly M.A. (2017). Design, synthesis, molecular modeling and anti-hyperglycemic evaluation of quinazolin-4(3H)-one derivatives as potential PPARc and SUR agonists. Bioorg. Med. Chem..

[63] Jacobs W.A., Heidelberger M. (1917). Methods for the acylation of aromatic amino compounds and ureas, with especial reference to chloroacetylation. J. Am. Chem. Soc..

[64] McTigue M., Murray B.W., Chen J.H., Deng Y., Solowiej J., Kania R.S. (2012). Molecular conformations, interactions, and properties associated with drug efficiency and clinical performance among VEGFR TK inhibitors. Proc. Natl. Acad. Sci. USA.

[65] Nazreen S. (2021). Design, synthesis, and molecular docking studies of thiazolidinediones as PPAR-γ agonists and thymidylate synthase inhibitors. Arch. Pharm..

[66] Mosmann T. (1983). Rapid colorimetric assay for cellular growth and survival: Application to proliferation and cytotoxicity assays. J. Immunol. Methods.

[67] Abou-Seri S.M., Eldehna W.M., Ali M.M., Abou El Ella D.A. (2016). 1-Piperazinylphthalazines as potential VEGFR-2 inhibitors and anticancer agents: Synthesis and in vitro biological evaluation. Eur. J. Med. Chem..

[68] Saleh N.M., Abdel-Rahman A.A.-H., Omar A.M., Khalifa M.M., El-Adl K. (2021). Pyridine-derived VEGFR-2 inhibitors: Rational design, synthesis, anticancer evaluations, in silico ADMET profile, and molecular docking. Arch. Pharm..

[69] Jameson D.M., Mocz G. (2005). Fluorescence polarization/anisotropy approaches to study protein-ligand interactions. Protein-Ligand Interactions: Methods in Molecular Biology.

[70] Schmidt S.D., Mazzella M.J., Nixon R.A., Mathews P.M. (2012). Aβ measurement by enzyme-linked immunosorbent assay. Amyloid Proteins: Methods and Protocols.

[71] Lipinski C.A., Lombardo F., Dominy B.W., Feeney P.J. (1997). Experimental and computational approaches to estimate solubility and permeability in drug discovery and development settings. Adv. Drug Deliv. Rev..

[72] Pires D.E.V., Blundell T.L., Ascher D.B. (2015). Predicting Small-Molecule Pharmacokinetic and Toxicity Properties Using Graph-Based Signatures. J. Med. Chem..

[73] Beig A., Agbaria R., Dahan A. (2013). Oral Delivery of Lipophilic Drugs: The Tradeoff between Solubility Increase and Permeability Decrease When Using Cyclodextrin-Based Formulations. PLoS ONE.

