# Supplementary material for: Design, Molecular Docking, Synthesis, Anticancer and Anti-Hyperglycemic Assessments of Thiazolidine-2,4-diones Bearing Sulfonylthiourea Moieties as Potent VEGFR-2 Inhibitors and PPARγ Agonists
Source: Pharmaceuticals (Basel). 2022 Feb 14;15(2):226. doi: 10.3390/ph15020226 (PMC8880361; doi:10.3390/ph15020226)

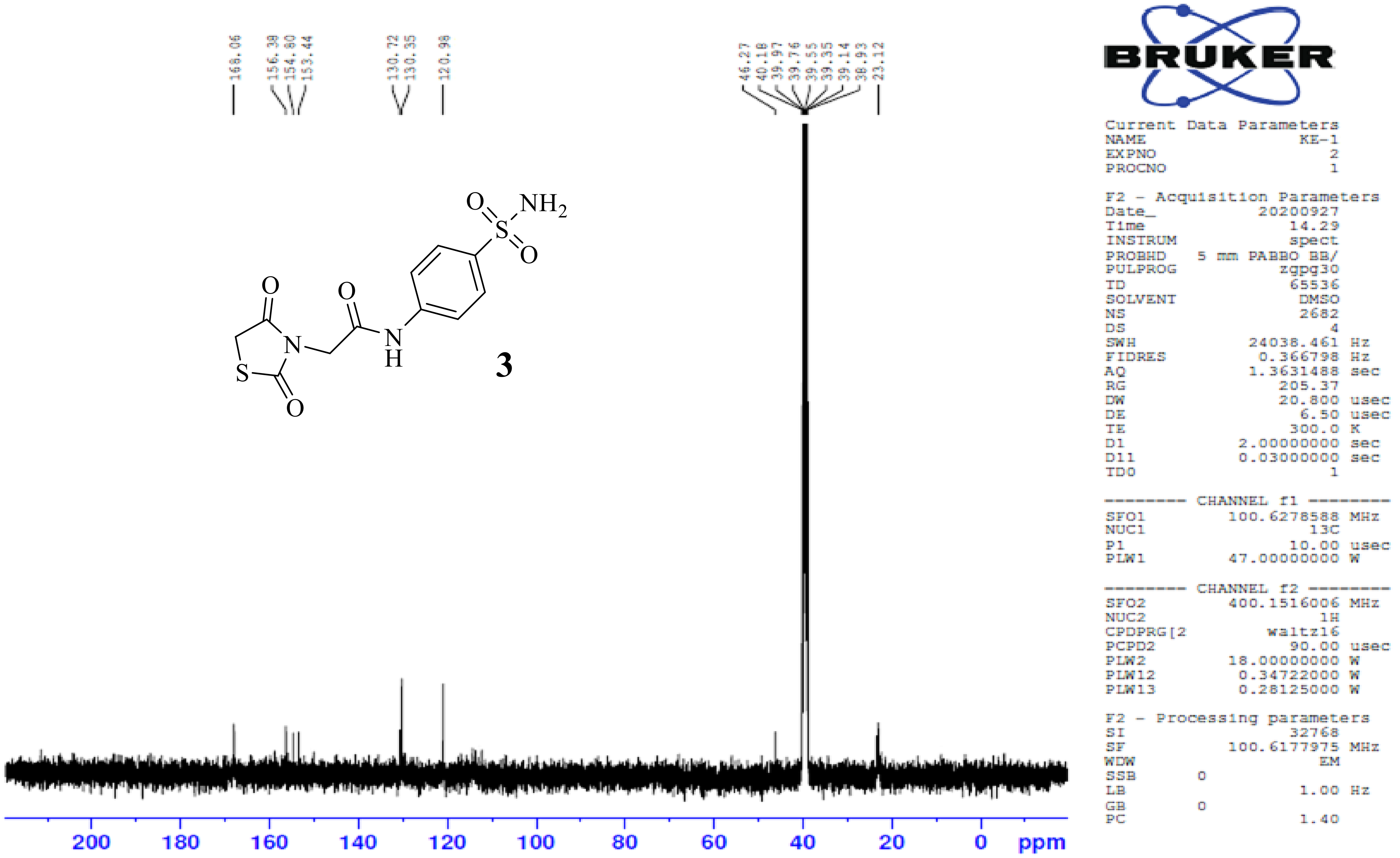
168.06
156.39
154.80
153.44
130.72
130.35
120.98
46.27
40.18
39.97
39.76
39.55
39.35
39.14
38.93
23.12
O
NH2
S
O
O
O
N
N
H
S
O
3
200
180
160
140
120
100
80
60
40
20
0
ppm
BRUKER
Current Data Parameters
NAME KE-1
EXPNO 2
PROCNO 1
F2 - Acquisition Parameters
Date_ 20200927
Time 14.29
INSTRUM spect
PROBHD 5 mm PABBO BB/
PULPROG zgpg30
TD 65536
SOLVENT DMSO
NS 2682
DS 4
SWH 24038.461 Hz
FIDRES 0.366798 Hz
AQ 1.3631488 sec
RG 205.37
DW 20.800 usec
DE 6.50 usec
TE 300.0 K
D1 2.00000000 sec
D11 0.03000000 sec
TD0 1
======== CHANNEL f1 ========
SFO1 100.6278588 MHz
NUC1 13C
P1 10.00 usec
PLW1 47.00000000 W
======== CHANNEL f2 ========
SFO2 400.1516006 MHz
NUC2 1H
CPDPRG[2 waltz16
PCPD2 90.00 usec
PLW2 18.00000000 W
PLW12 0.34722000 W
PLW13 0.28125000 W
F2 - Processing parameters
SI 32768
SF 100.6177975 MHz
WDW EM
SSB 0
LB 1.00 Hz
GB 0
PC 1.40

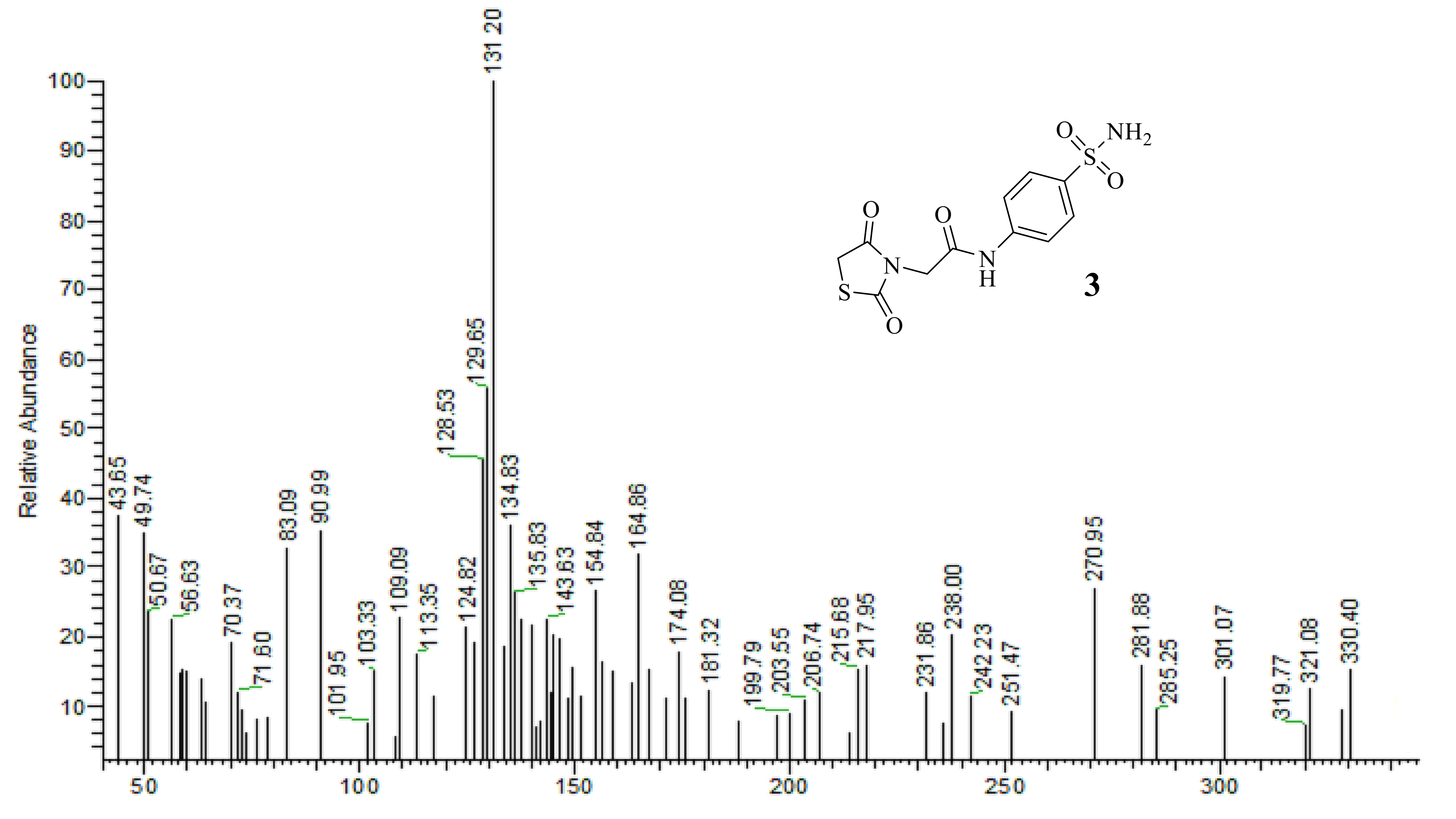
Relative Abundance
100
90
80
70
60
50
40
30
20
10
50
100
150
200
250
300
131.20
129.65
128.53
43.65
49.74
50.67
56.63
70.37
71.60
83.09
90.99
101.95
103.33
109.09
113.35
124.82
134.83
135.83
143.63
154.84
164.86
174.08
181.32
199.79
203.55
206.74
215.68
217.95
231.86
238.00
242.23
251.47
270.95
281.88
285.25
301.07
319.77
321.08
330.40
O
NH2
S
O
O
O
N
H
S
O
3

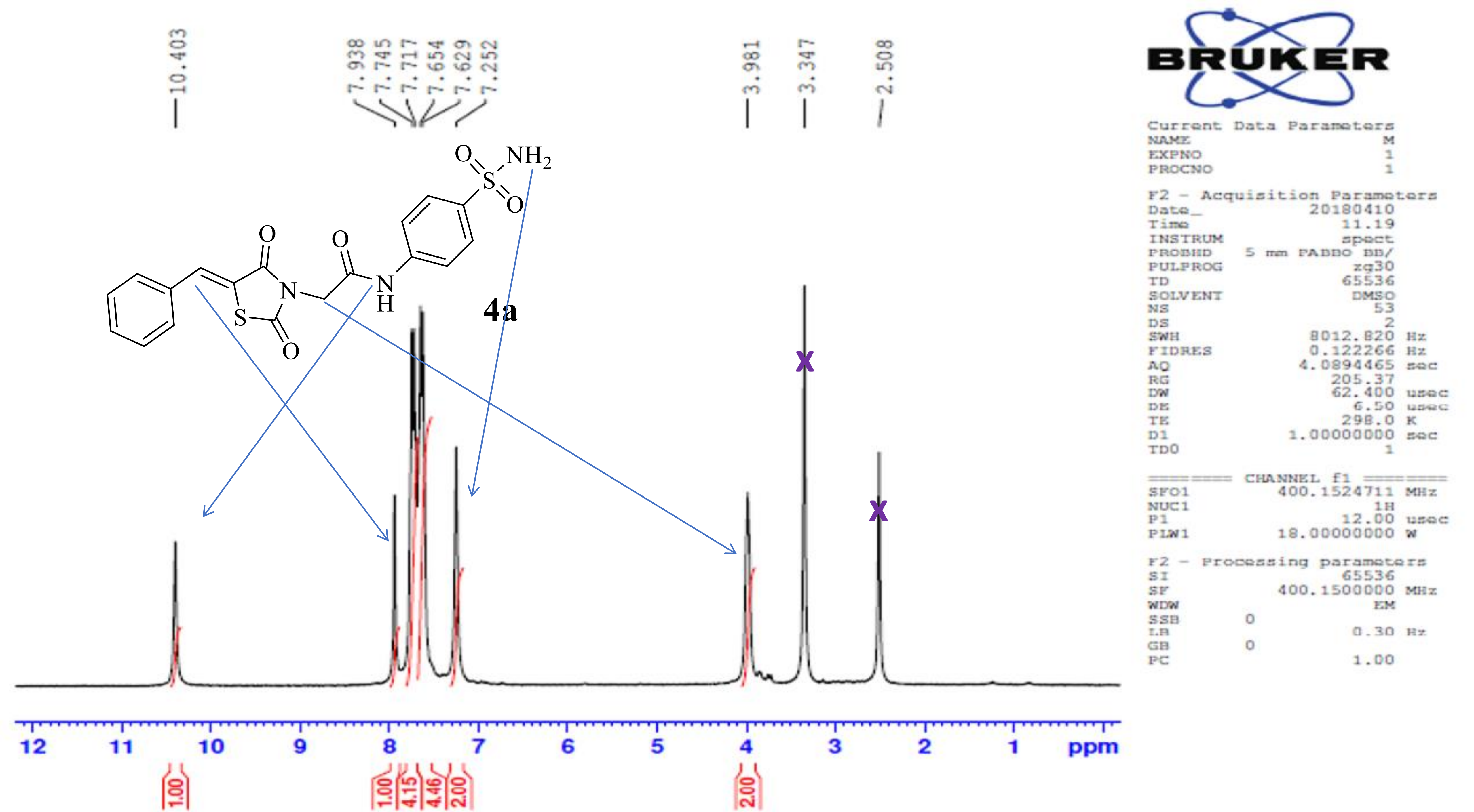
10.403
7.938
7.745
7.717
7.654
7.629
7.252
3.981
3.347
2.508
4a
BRUKER
Current Data Parameters
NAME M
EXPNO 1
PROCNO 1
F2 - Acquisition Parameters
Date_ 20180410
Time 11.19
INSTRUM spect
PROBHD 5 mm PABBO BB/
PULPROG zg30
TD 65536
SOLVENT DMSO
NS 53
DS 2
SWH 8012.820 Hz
FIDRES 0.122266 Hz
AQ 4.0894465 sec
RG 205.37
DW 62.400 usec
DE 6.50 usec
TE 298.0 K
D1 1.00000000 sec
TD0 1
======= CHANNEL f1 =======
SFO1 400.1524711 MHz
NUC1 1H
P1 12.00 usec
PLW1 18.00000000 W
F2 - Processing parameters
SI 65536
SF 400.1500000 MHz
WDW EM
SSB 0
LB 0.30 Hz
GB 0
PC 1.00
12 11 10 9 8 7 6 5 4 3 2 1 ppm
1.00
1.00
4.15
4.46
2.00
2.00

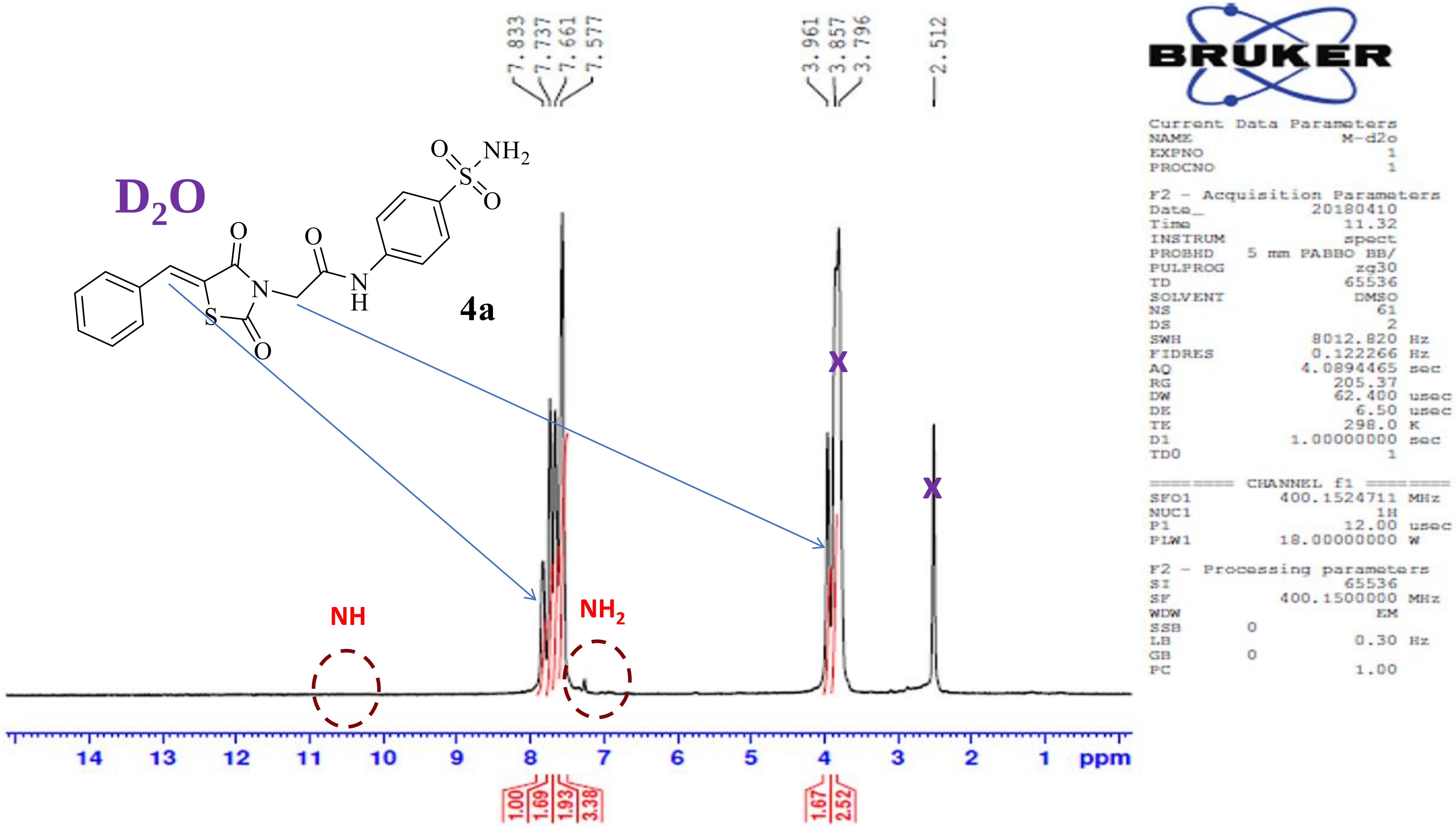
D2O
4a
NH
NH2
X
X
7.833
7.737
7.661
7.577
3.961
3.857
3.796
2.512
14 13 12 11 10 9 8 7 6 5 4 3 2 1 ppm
1.00
1.69
1.93
3.38
1.67
2.52
BRUKER
Current Data Parameters
NAME M-d2o
EXPNO 1
PROCNO 1
F2 - Acquisition Parameters
Date_ 20180410
Time 11.32
INSTRUM spect
PROBHD 5 mm PABBO BB/
PULPROG zg30
TD 65536
SOLVENT DMSO
NS 61
DS 2
SWH 8012.820 Hz
FIDRES 0.122266 Hz
AQ 4.0894465 sec
RG 205.37
DW 62.400 usec
DE 6.50 usec
TE 298.0 K
D1 1.00000000 sec
TD0 1
======== CHANNEL f1 ========
SFO1 400.1524711 MHz
NUC1 1H
P1 12.00 usec
PLW1 18.00000000 W
F2 - Processing parameters
SI 65536
SF 400.1500000 MHz
WDW EM
SSB 0
LB 0.30 Hz
GB 0
PC 1.00

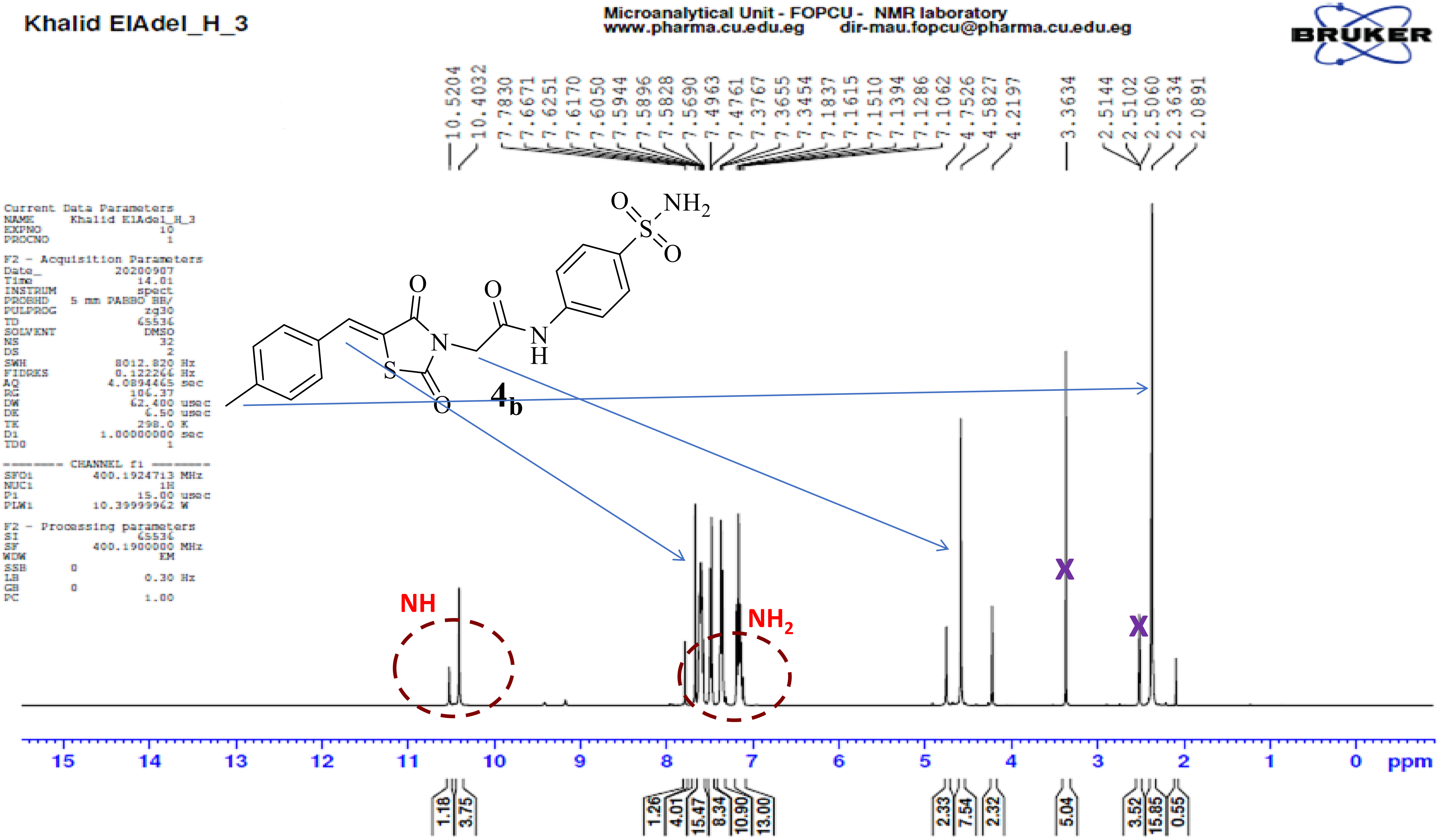
Khalid ElAdel_H_3
Microanalytical Unit - FOPCU - NMR laboratory
www.pharma.cu.edu.eg dir-mau.fopcu@pharma.cu.edu.eg
BRUKER
10.5204
10.4032
7.7830
7.6671
7.6251
7.6170
7.6050
7.5944
7.5896
7.5828
7.5690
7.4963
7.4761
7.3767
7.3655
7.3454
7.1837
7.1615
7.1510
7.1394
7.1286
7.1062
4.7526
4.5827
4.2197
3.3634
2.5144
2.5102
2.5060
2.3634
2.0891
Current Data Parameters
NAME Khalid ElAdel_H_3
EXPNO 10
PROCNO 1
F2 - Acquisition Parameters
Date_ 20200907
Time 14.01
INSTRUM spect
PROBHD 5 mm PABBO BB/
PULPROG zg30
TD 65536
SOLVENT DMSO
NS 32
DS 2
SWH 8012.820 Hz
FIDRES 0.122266 Hz
AQ 4.0894465 sec
RG 106.37
DW 62.400 usec
DE 6.50 usec
TE 298.0 K
D1 1.00000000 sec
TD0 1
======== CHANNEL f1 ========
SFO1 400.1924713 MHz
NUC1 1H
P1 15.00 usec
PLW1 10.39999962 W
F2 - Processing parameters
SI 65536
SF 400.1900000 MHz
WDW EM
SSB 0
LB 0.30 Hz
GB 0
PC 1.00
O
NH2
S
O
O
O
N
H
N
S
O
O
4b
NH
NH2
X
X
15
14
13
12
11
10
9
8
7
6
5
4
3
2
1
0
ppm
1.18
3.75
1.26
4.01
15.47
8.34
10.90
13.00
2.33
7.54
2.32
5.04
3.52
15.85
0.55

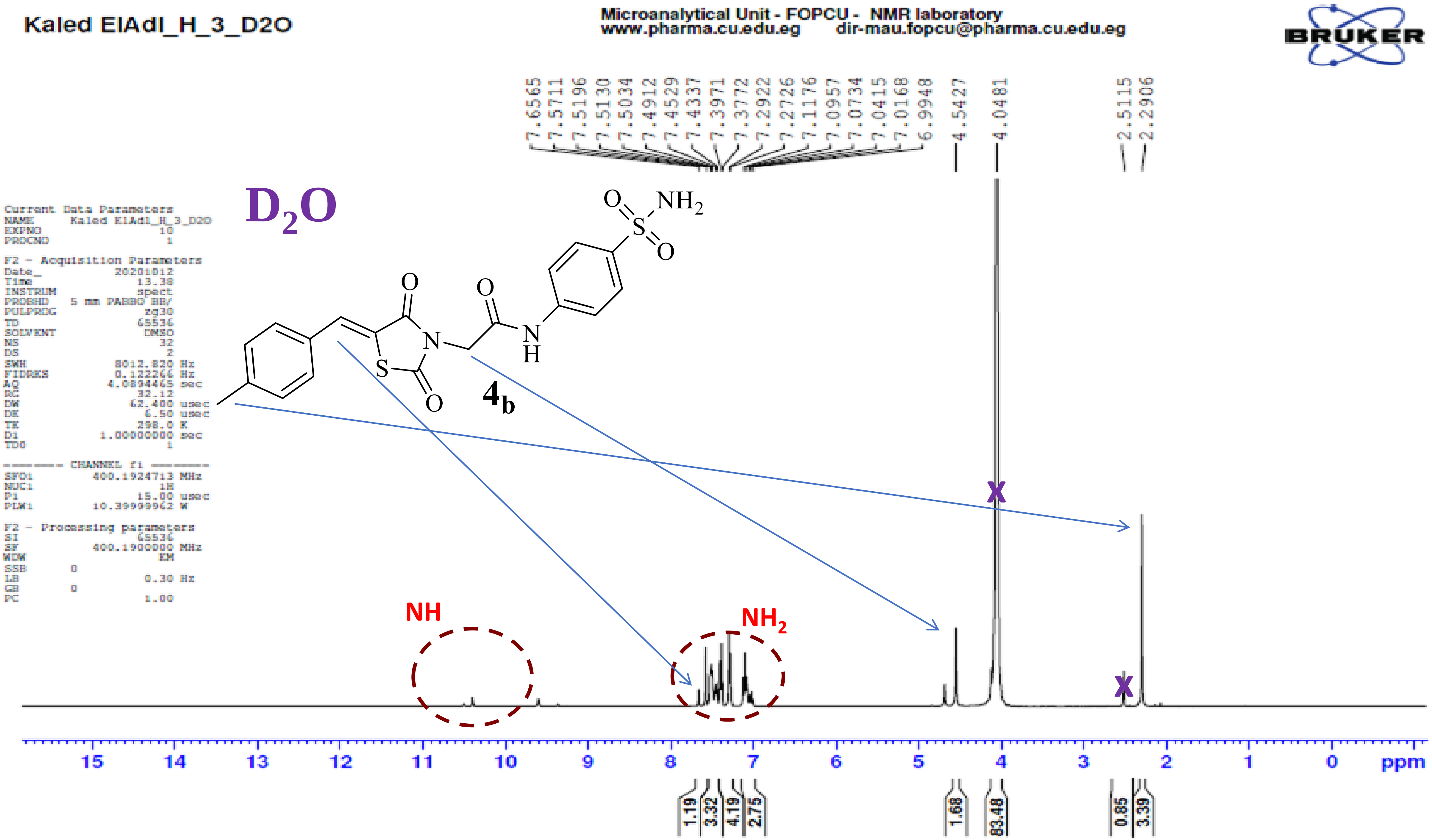
Kaled ElAdl_H_3_D2O
Microanalytical Unit - FOPCU - NMR laboratory
www.pharma.cu.edu.eg dir-mau.fopcu@pharma.cu.edu.eg
BRUKER
D2O
Current Data Parameters
NAME Kaled ElAdl_H_3_D2O
EXPNO 10
PROCNO 1
F2 - Acquisition Parameters
Date_ 20201012
Time 13.38
INSTRUM spect
PROBHD 5 mm PABBO BB/
PULPROG zg30
TD 65536
SOLVENT DMSO
NS 32
DS 2
SWH 8012.820 Hz
FIDRES 0.122266 Hz
AQ 4.0894465 sec
RG 32.12
DW 62.400 usec
DE 6.50 usec
TE 298.0 K
D1 1.00000000 sec
TD0 1
======== CHANNEL f1 ========
SFO1 400.1924713 MHz
NUC1 1H
P1 15.00 usec
PLW1 10.39999962 W
F2 - Processing parameters
SI 65536
SF 400.1900000 MHz
WDW EM
SSB 0
LB 0.30 Hz
GB 0
PC 1.00
4b
NH
NH2
7.6565 7.5711 7.5196 7.5130 7.5034 7.4912 7.4529 7.4337 7.3971 7.3772 7.2922 7.2726 7.1176 7.0957 7.0734 7.0415 7.0168 6.9948
4.5427 4.0481
2.5115 2.2906
15 14 13 12 11 10 9 8 7 6 5 4 3 2 1 0 ppm
1.19 3.32 4.19 2.75 1.68 83.48 0.85 3.39

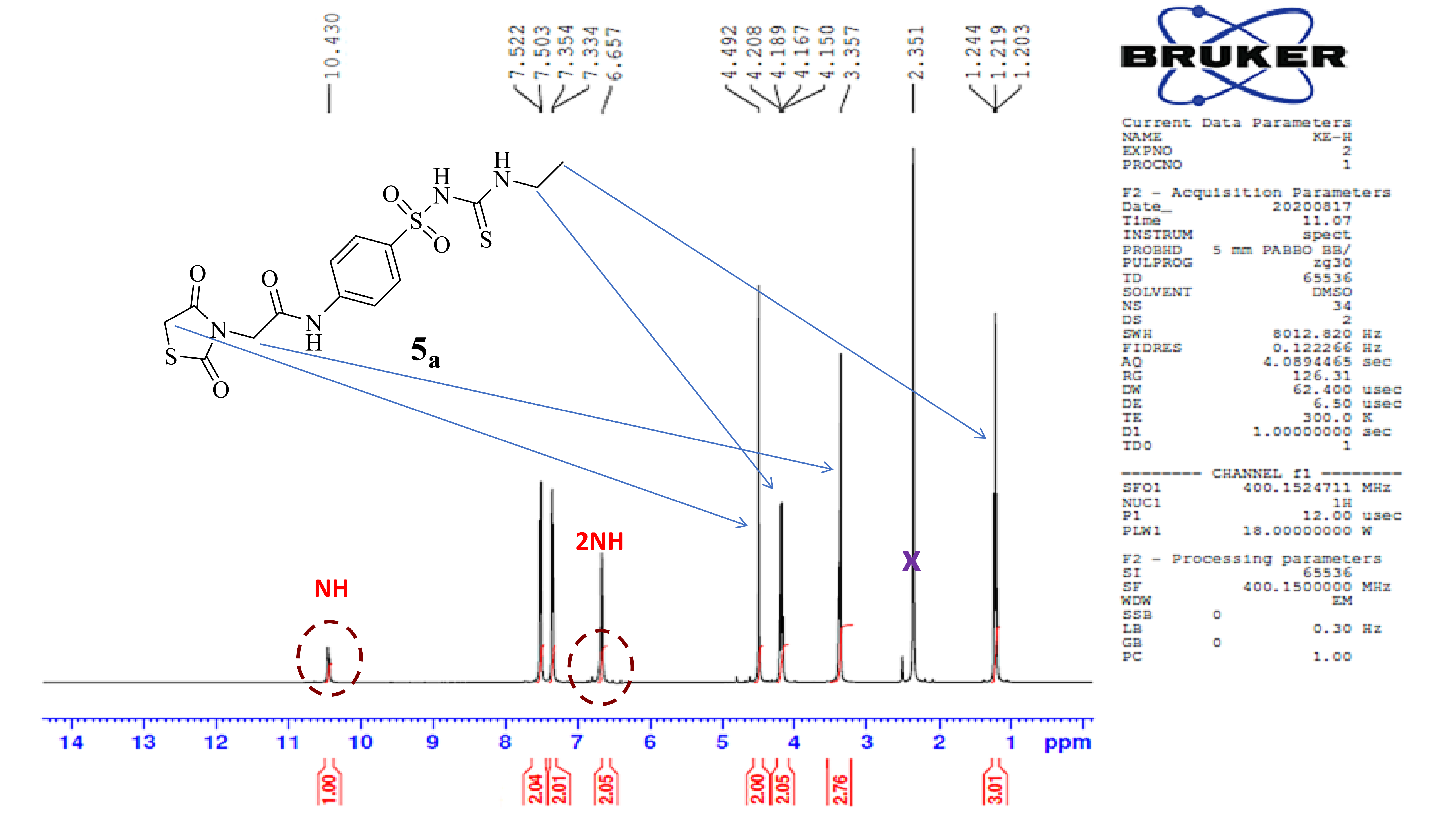
10.430
7.522
7.503
7.354
7.334
6.657
4.492
4.208
4.189
4.167
4.150
3.357
2.351
1.244
1.219
1.203
5a
NH
2NH
X
1.00
2.04
2.01
2.05
2.00
2.05
2.76
3.01
14 13 12 11 10 9 8 7 6 5 4 3 2 1 ppm
BRUKER
Current Data Parameters
NAME KE-H
EXPNO 2
PROCNO 1
F2 - Acquisition Parameters
Date_ 20200817
Time 11.07
INSTRUM spect
PROBHD 5 mm PABBO BB/
PULPROG zg30
TD 65536
SOLVENT DMSO
NS 34
DS 2
SWH 8012.820 Hz
FIDRES 0.122266 Hz
AQ 4.0894465 sec
RG 126.31
DW 62.400 usec
DE 6.50 usec
TE 300.0 K
D1 1.00000000 sec
TD0 1
======== CHANNEL f1 ========
SFO1 400.1524711 MHz
NUC1 1H
P1 12.00 usec
PLW1 18.00000000 W
F2 - Processing parameters
SI 65536
SF 400.1500000 MHz
WDW EM
SSB 0
LB 0.30 Hz
GB 0
PC 1.00

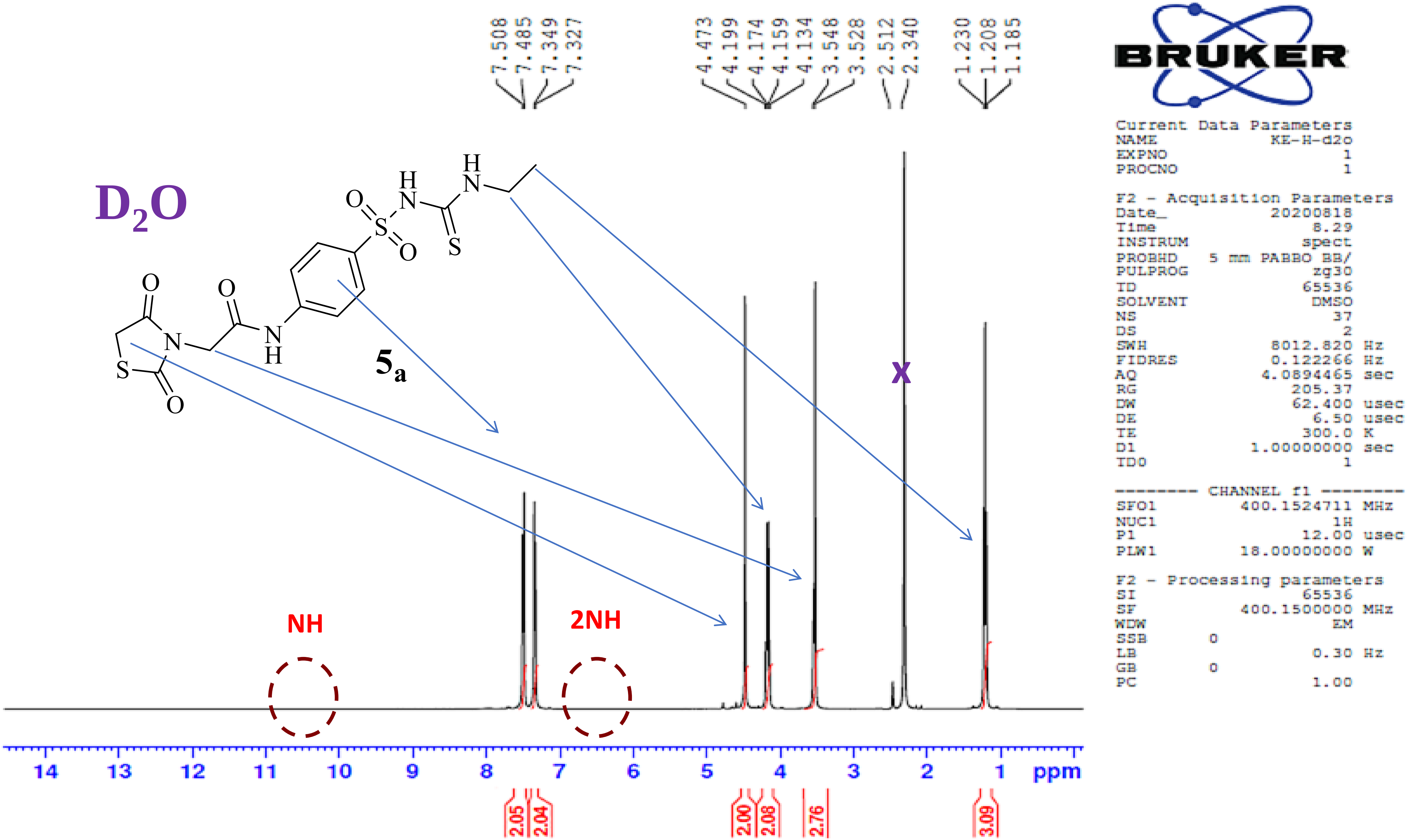

D2O
5a
7.508
7.485
7.349
7.327
4.473
4.199
4.174
4.159
4.134
3.548
3.528
2.512
2.340
1.230
1.208
1.185
NH
2NH
X
14 13 12 11 10 9 8 7 6 5 4 3 2 1 ppm
2.05 2.04 2.00 2.08 2.76 3.09
BRUKER
Current Data Parameters
NAME KE-H-d2o
EXPNO 1
PROCNO 1
F2 - Acquisition Parameters
Date_ 20200818
Time 8.29
INSTRUM spect
PROBHD 5 mm PABBO BB/
PULPROG zg30
TD 65536
SOLVENT DMSO
NS 37
DS 2
SWH 8012.820 Hz
FIDRES 0.122266 Hz
AQ 4.0894465 sec
RG 205.37
DW 62.400 usec
DE 6.50 usec
TE 300.0 K
D1 1.00000000 sec
TD0 1
======== CHANNEL f1 ========
SFO1 400.1524711 MHz
NUC1 1H
P1 12.00 usec
PLW1 18.00000000 W
F2 - Processing parameters
SI 65536
SF 400.1500000 MHz
WDW EM
SSB 0
LB 0.30 Hz
GB 0
PC 1.00

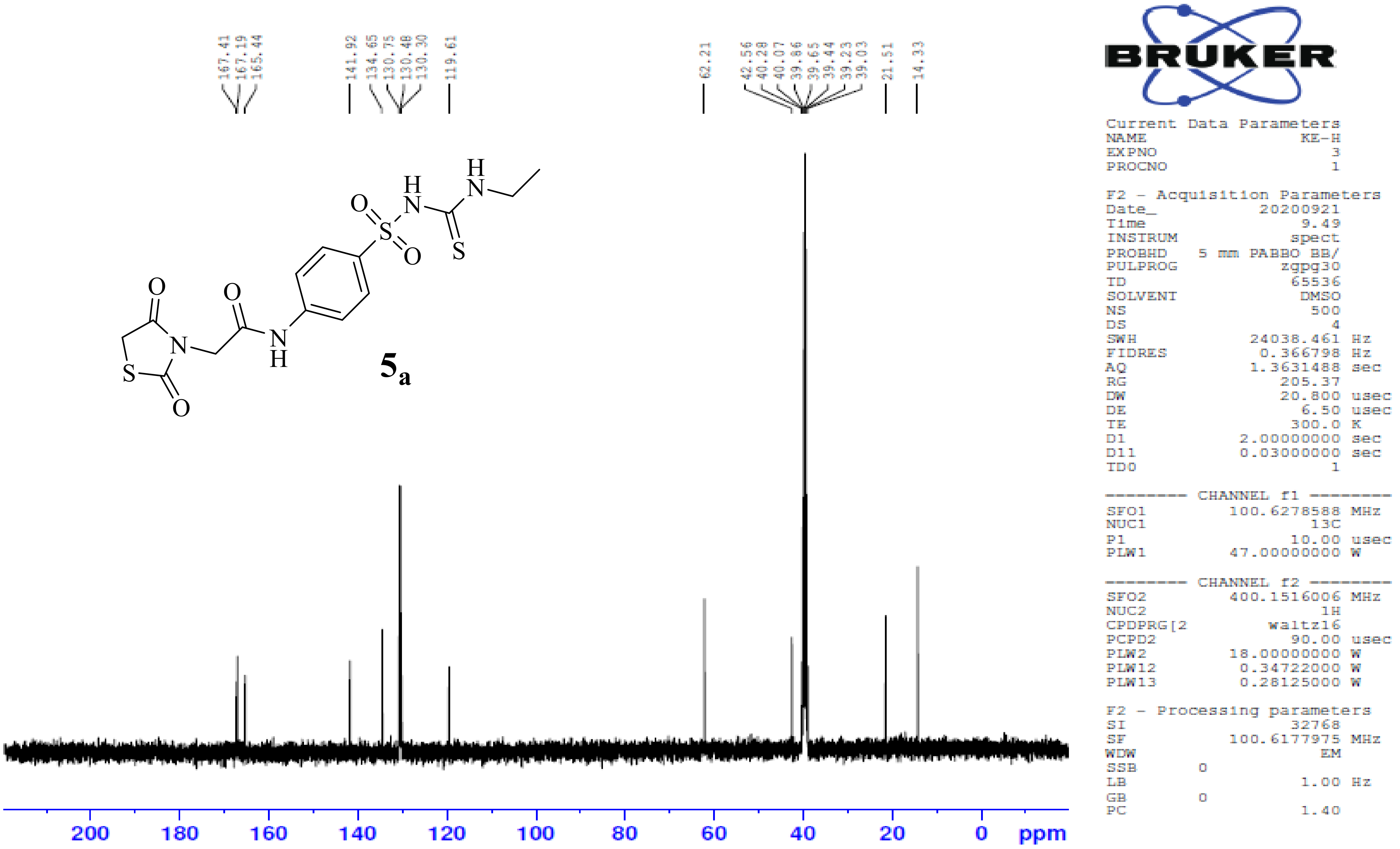
167.41
167.19
165.44
141.92
134.65
130.75
130.40
130.30
119.61
62.21
42.56
40.28
40.07
39.86
39.65
39.44
39.23
39.03
21.51
14.33
5a
200 180 160 140 120 100 80 60 40 20 0 ppm
BRUKER
Current Data Parameters
NAME KE-H
EXPNO 3
PROCNO 1
F2 - Acquisition Parameters
Date_ 20200921
Time 9.49
INSTRUM spect
PROBHD 5 mm PABBO BB/
PULPROG zgpg30
TD 65536
SOLVENT DMSO
NS 500
DS 4
SWH 24038.461 Hz
FIDRES 0.366798 Hz
AQ 1.3631488 sec
RG 205.37
DW 20.800 usec
DE 6.50 usec
TE 300.0 K
D1 2.00000000 sec
D11 0.03000000 sec
TD0 1
======== CHANNEL f1 ========
SFO1 100.6278588 MHz
NUC1 13C
P1 10.00 usec
PLW1 47.00000000 W
======== CHANNEL f2 ========
SFO2 400.1516006 MHz
NUC2 1H
CPDPRG[2 waltz16
PCPD2 90.00 usec
PLW2 18.00000000 W
PLW12 0.34722000 W
PLW13 0.28125000 W
F2 - Processing parameters
SI 32768
SF 100.6177975 MHz
WDW EM
SSB 0
LB 1.00 Hz
GB 0
PC 1.40

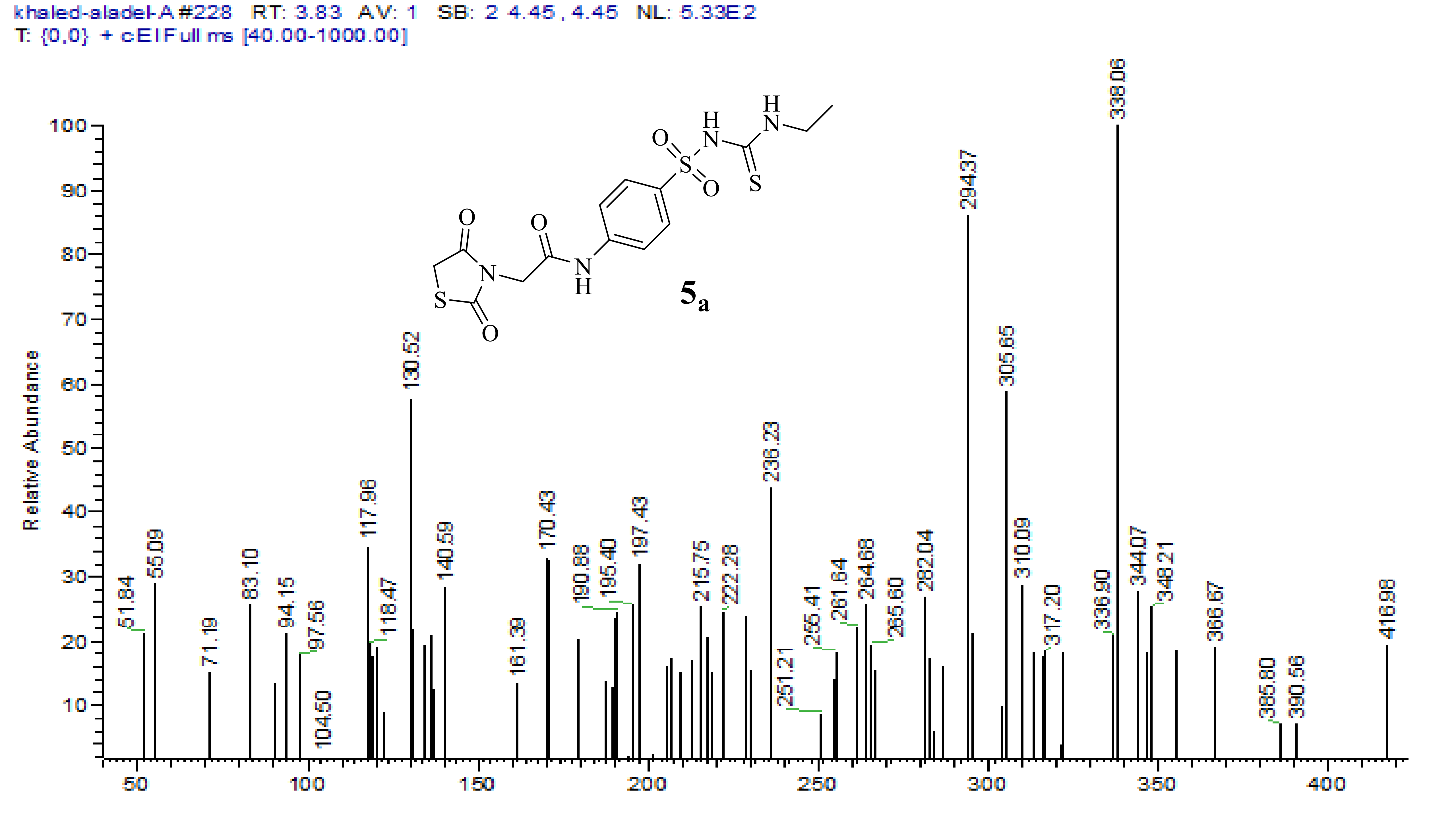
khaled-aladel-A#228 RT: 3.83 AV: 1 SB: 2 4.45 , 4.45 NL: 5.33E2
T: {0,0} + c EI Full ms [40.00-1000.00]
Relative Abundance
338.06
294.37
305.65
130.52
236.23
117.96
170.43
197.43
140.59
55.09
83.10
94.15
97.56
51.84
71.19
104.50
118.47
161.39
190.88
195.40
215.75
222.28
251.21
255.41
261.64
264.68
265.60
282.04
310.09
317.20
336.90
344.07
348.21
366.67
385.80
390.56
416.98
5a

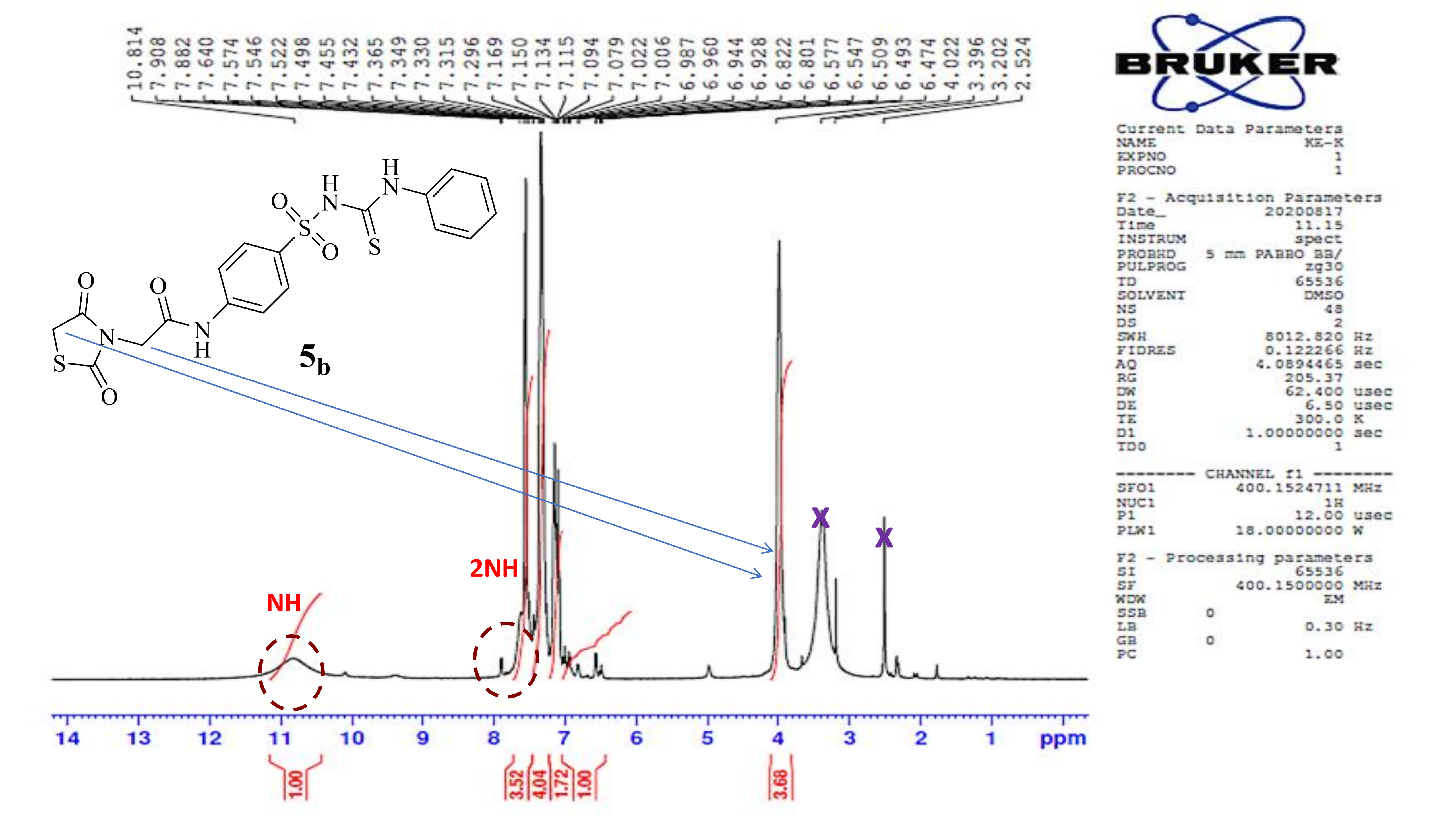
10.814
7.908
7.882
7.640
7.574
7.546
7.522
7.498
7.455
7.432
7.365
7.349
7.330
7.315
7.296
7.169
7.150
7.134
7.115
7.094
7.079
7.022
7.006
6.987
6.960
6.944
6.928
6.822
6.801
6.577
6.547
6.509
6.493
6.474
4.022
3.396
3.202
2.524
5b
NH
2NH
X
X
14
13
12
11
10
9
8
7
6
5
4
3
2
1
ppm
1.00
3.52
4.04
1.72
1.00
3.68
BRUKER
Current Data Parameters
NAME KE-K
EXPNO 1
PROCNO 1
F2 - Acquisition Parameters
Date_ 20200817
Time 11.15
INSTRUM spect
PROBHD 5 mm PABBO BB/
PULPROG zg30
TD 65536
SOLVENT DMSO
NS 48
DS 2
SWH 8012.820 Hz
FIDRES 0.122266 Hz
AQ 4.0894465 sec
RG 205.37
DW 62.400 usec
DE 6.50 usec
TE 300.0 K
D1 1.00000000 sec
TD0 1
======== CHANNEL f1 ========
SFO1 400.1524711 MHz
NUC1 1H
P1 12.00 usec
PLW1 18.00000000 W
F2 - Processing parameters
SI 65536
SF 400.1500000 MHz
WDW EM
SSB 0
LB 0.30 Hz
GB 0
PC 1.00

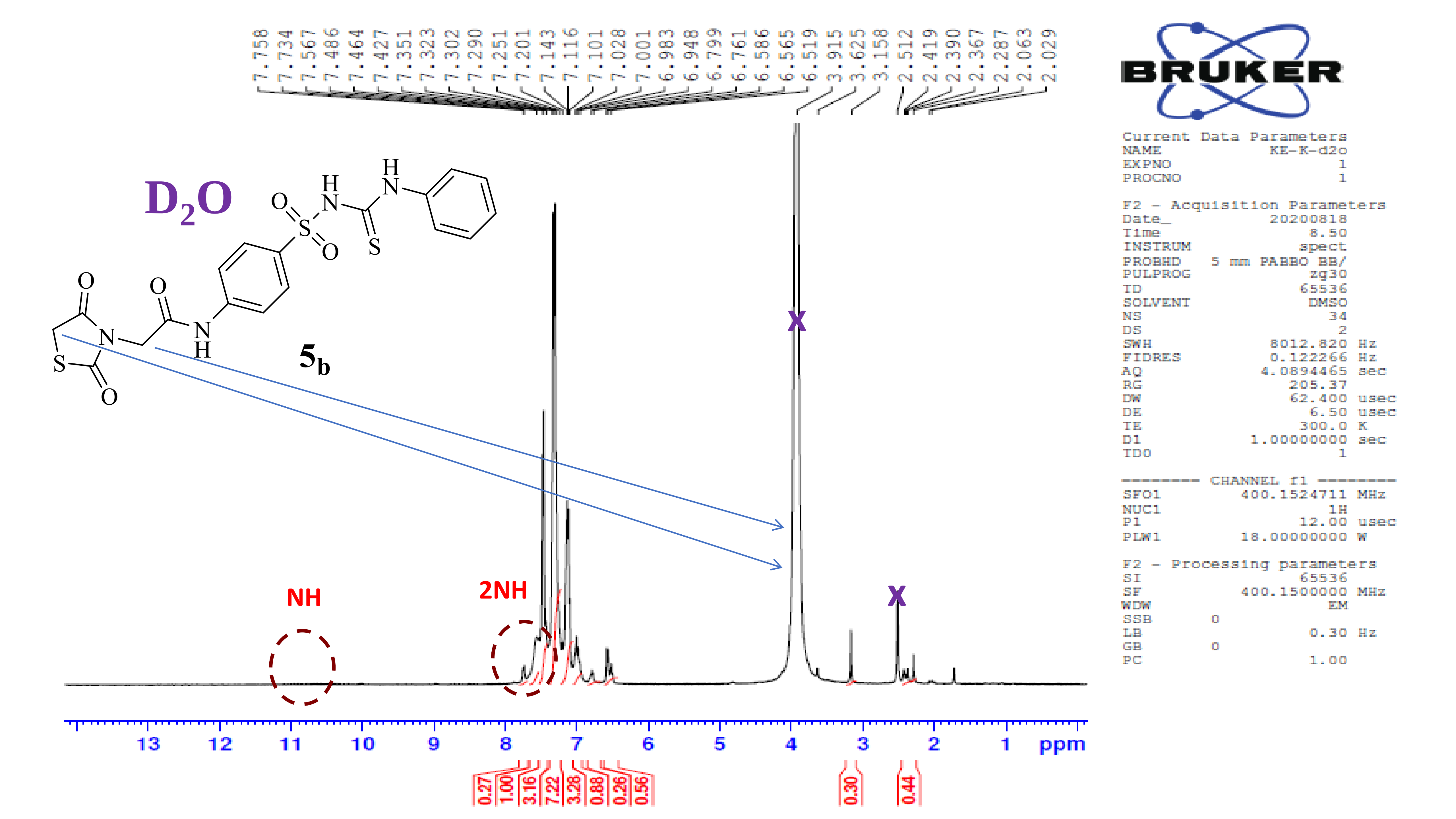

D2O
5b
NH
2NH
X
X
7.758 7.734 7.567 7.486 7.464 7.427 7.351 7.323 7.302 7.290 7.251 7.201 7.143 7.116 7.101 7.028 7.001 6.983 6.948 6.799 6.761 6.586 6.565 6.519 3.915 3.625 3.158 2.512 2.419 2.390 2.367 2.287 2.063 2.029
13 12 11 10 9 8 7 6 5 4 3 2 1 ppm
0.27 1.00 3.16 7.22 3.28 0.88 0.26 0.56 0.30 0.44
Current Data Parameters
NAME KE-K-d2o
EXPNO 1
PROCNO 1
F2 - Acquisition Parameters
Date_ 20200818
Time 8.50
INSTRUM spect
PROBHD 5 mm PABBO BB/
PULPROG zg30
TD 65536
SOLVENT DMSO
NS 34
DS 2
SWH 8012.820 Hz
FIDRES 0.122266 Hz
AQ 4.0894465 sec
RG 205.37
DW 62.400 usec
DE 6.50 usec
TE 300.0 K
D1 1.00000000 sec
TD0 1
CHANNEL f1
SFO1 400.1524711 MHz
NUC1 1H
P1 12.00 usec
PLW1 18.00000000 W
F2 - Processing parameters
SI 65536
SF 400.1500000 MHz
WDW EM
SSB 0
LB 0.30 Hz
GB 0
PC 1.00

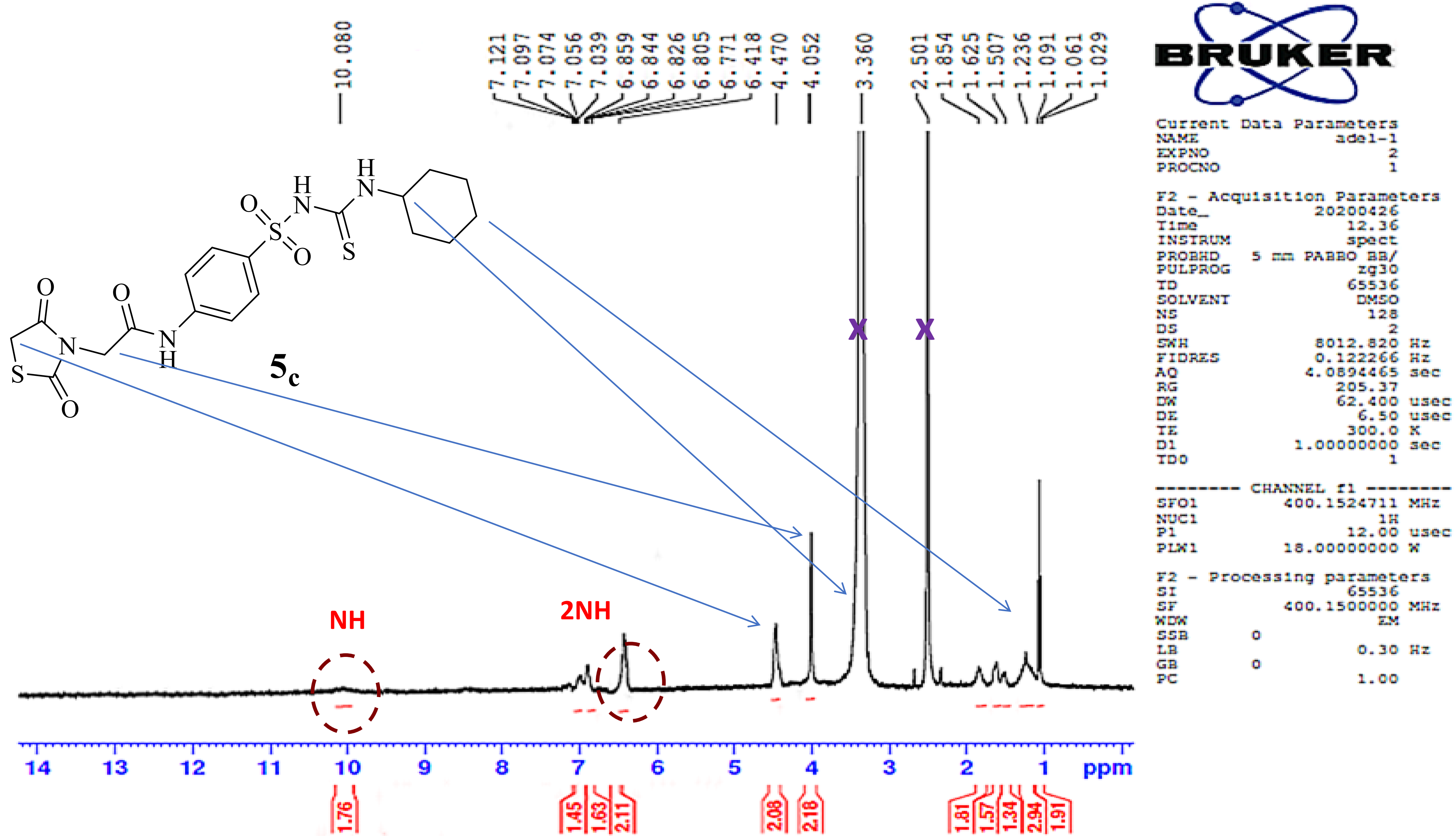

10.080
7.121 7.097 7.074 7.056 7.039 6.859 6.844 6.826 6.805 6.771 6.418
4.470
4.052
3.360
2.501 1.854 1.625 1.507 1.236 1.091 1.061 1.029
BRUKER
Current Data Parameters
NAME ade1-1
EXPNO 2
PROCNO 1
F2 - Acquisition Parameters
Date_ 20200426
Time 12.36
INSTRUM spect
PROBHD 5 mm PABBO BB/
PULPROG zg30
TD 65536
SOLVENT DMSO
NS 128
DS 2
SWH 8012.820 Hz
FIDRES 0.122266 Hz
AQ 4.0894465 sec
RG 205.37
DW 62.400 usec
DE 6.50 usec
TE 300.0 K
D1 1.00000000 sec
TD0 1
CHANNEL f1
SFO1 400.1524711 MHz
NUC1 1H
P1 12.00 usec
PLW1 18.00000000 W
F2 - Processing parameters
SI 65536
SF 400.1500000 MHz
WDW EM
SSB 0
LB 0.30 Hz
GB 0
PC 1.00
5c
NH
2NH
14 13 12 11 10 9 8 7 6 5 4 3 2 1 ppm
1.76
1.45 1.63 2.11
2.08 2.18
1.81 1.57 1.34 2.94 1.91

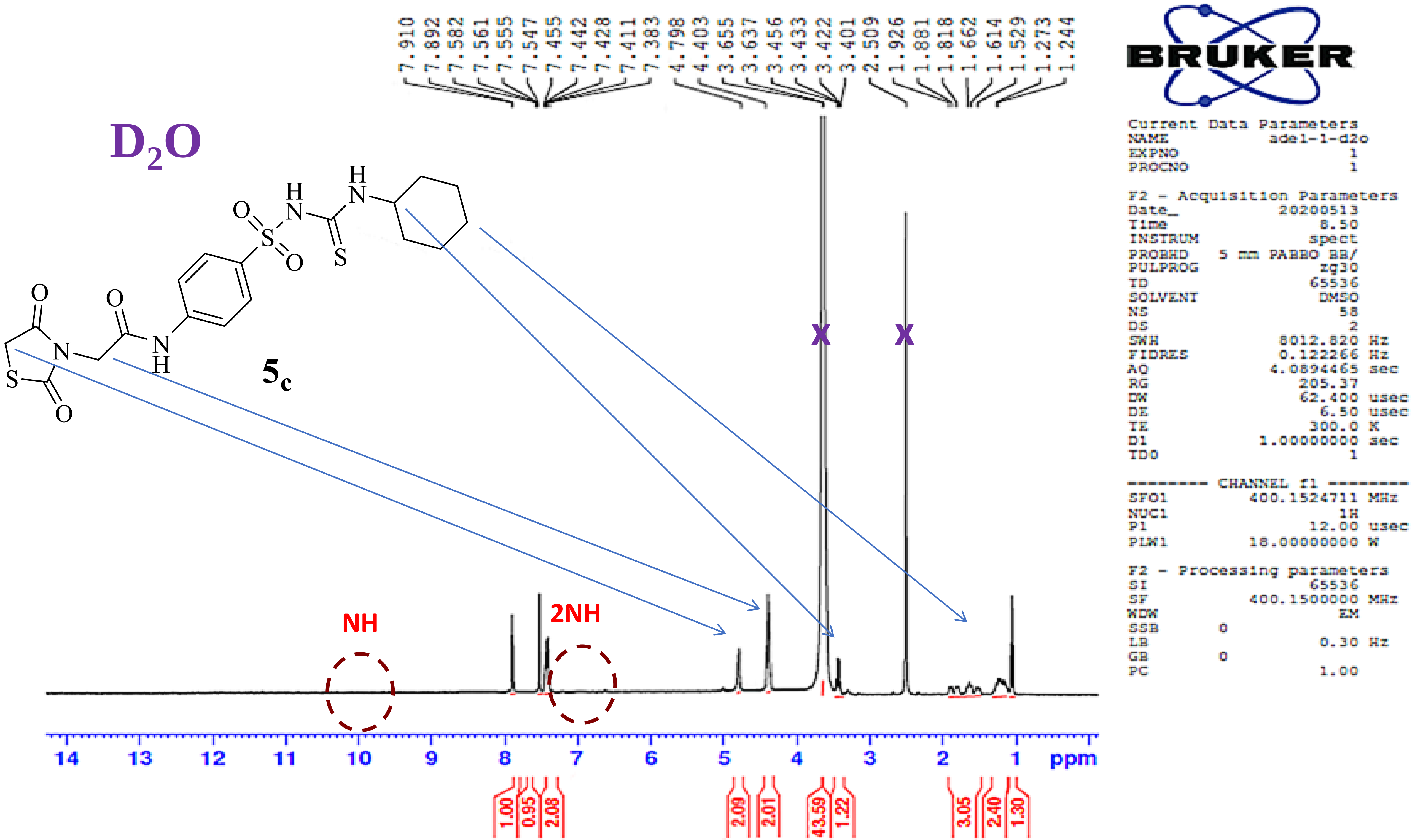
D2O
5c
NH
2NH
X
X
7.910 7.892 7.582 7.561 7.555 7.547 7.455 7.442 7.428 7.411 7.383 4.798 4.403 3.655 3.637 3.456 3.433 3.422 3.401 2.509 1.926 1.881 1.818 1.662 1.614 1.529 1.273 1.244
14 13 12 11 10 9 8 7 6 5 4 3 2 1 ppm
1.00 0.95 2.08 2.09 2.01 43.59 1.22 3.05 2.40 1.30
BRUKER
Current Data Parameters
NAME ade1-1-d2o
EXPNO 1
PROCNO 1
F2 - Acquisition Parameters
Date_ 20200513
Time 8.50
INSTRUM spect
PROBHD 5 mm PABBO BB/
PULPROG zg30
TD 65536
SOLVENT DMSO
NS 58
DS 2
SWH 8012.820 Hz
FIDRES 0.122266 Hz
AQ 4.0894465 sec
RG 205.37
DW 62.400 usec
DE 6.50 usec
TE 300.0 K
D1 1.00000000 sec
TD0 1
CHANNEL f1
SFO1 400.1524711 MHz
NUC1 1H
P1 12.00 usec
PLW1 18.00000000 W
F2 - Processing parameters
SI 65536
SF 400.1500000 MHz
WDW EM
SSB 0
LB 0.30 Hz
GB 0
PC 1.00

khaled-aladel-b #147 RT: 2.48 AV: 1 SB: 2 3.90 , 3.90 NL: 4.00E2
T: {0,0} + c EI Full ms [40.00-1000.00]

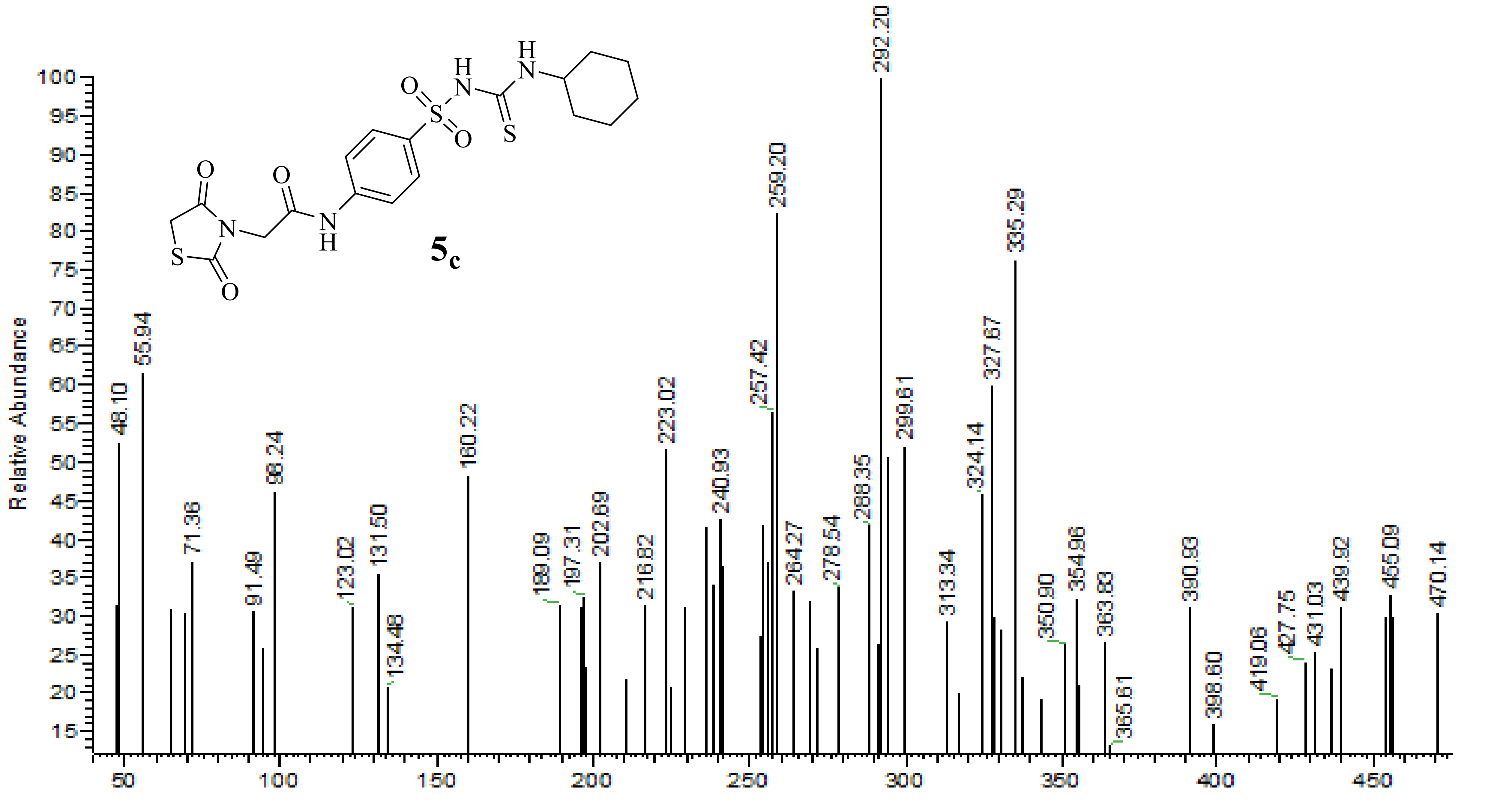

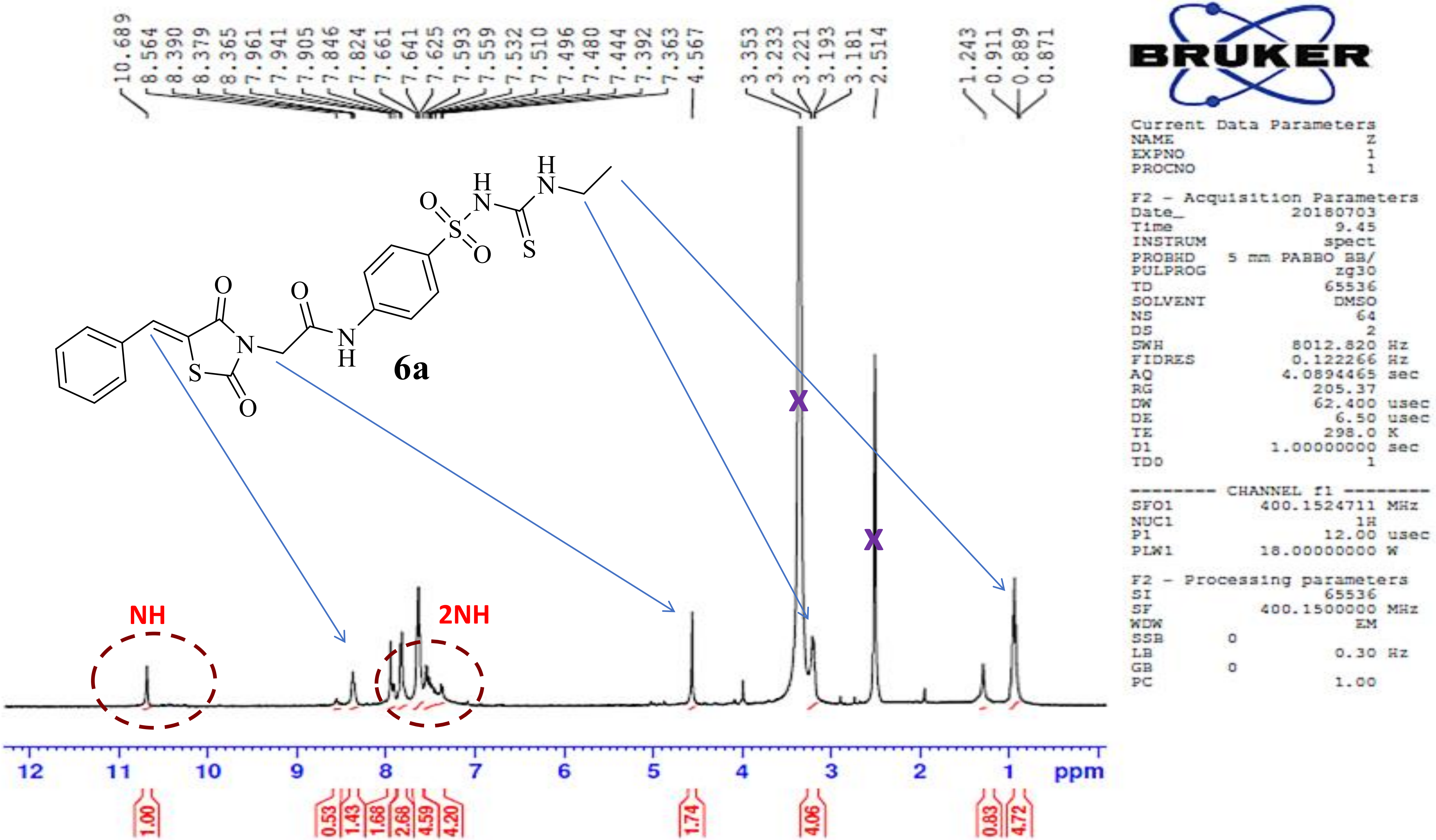
10.689
8.564
8.390
8.379
8.365
7.961
7.941
7.905
7.846
7.824
7.661
7.641
7.625
7.593
7.559
7.532
7.510
7.496
7.480
7.444
7.392
7.363
4.567
3.353
3.233
3.221
3.193
3.181
2.514
1.243
0.911
0.889
0.871
6a
NH
2NH
X
X
12
11
10
9
8
7
6
5
4
3
2
1
ppm
1.00
0.53
1.43
1.68
2.68
4.59
4.20
1.74
4.06
0.83
4.72
BRUKER
Current Data Parameters
NAME Z
EXPNO 1
PROCNO 1
F2 - Acquisition Parameters
Date_ 20180703
Time 9.45
INSTRUM spect
PROBHD 5 mm PABBO BB/
PULPROG zg30
TD 65536
SOLVENT DMSO
NS 64
DS 2
SWH 8012.820 Hz
FIDRES 0.122266 Hz
AQ 4.0894465 sec
RG 205.37
DW 62.400 usec
DE 6.50 usec
TE 298.0 K
D1 1.00000000 sec
TD0 1
======== CHANNEL f1 ========
SFO1 400.1524711 MHz
NUC1 1H
P1 12.00 usec
PLW1 18.00000000 W
F2 - Processing parameters
SI 65536
SF 400.1500000 MHz
WDW EM
SSB 0
LB 0.30 Hz
GB 0
PC 1.00

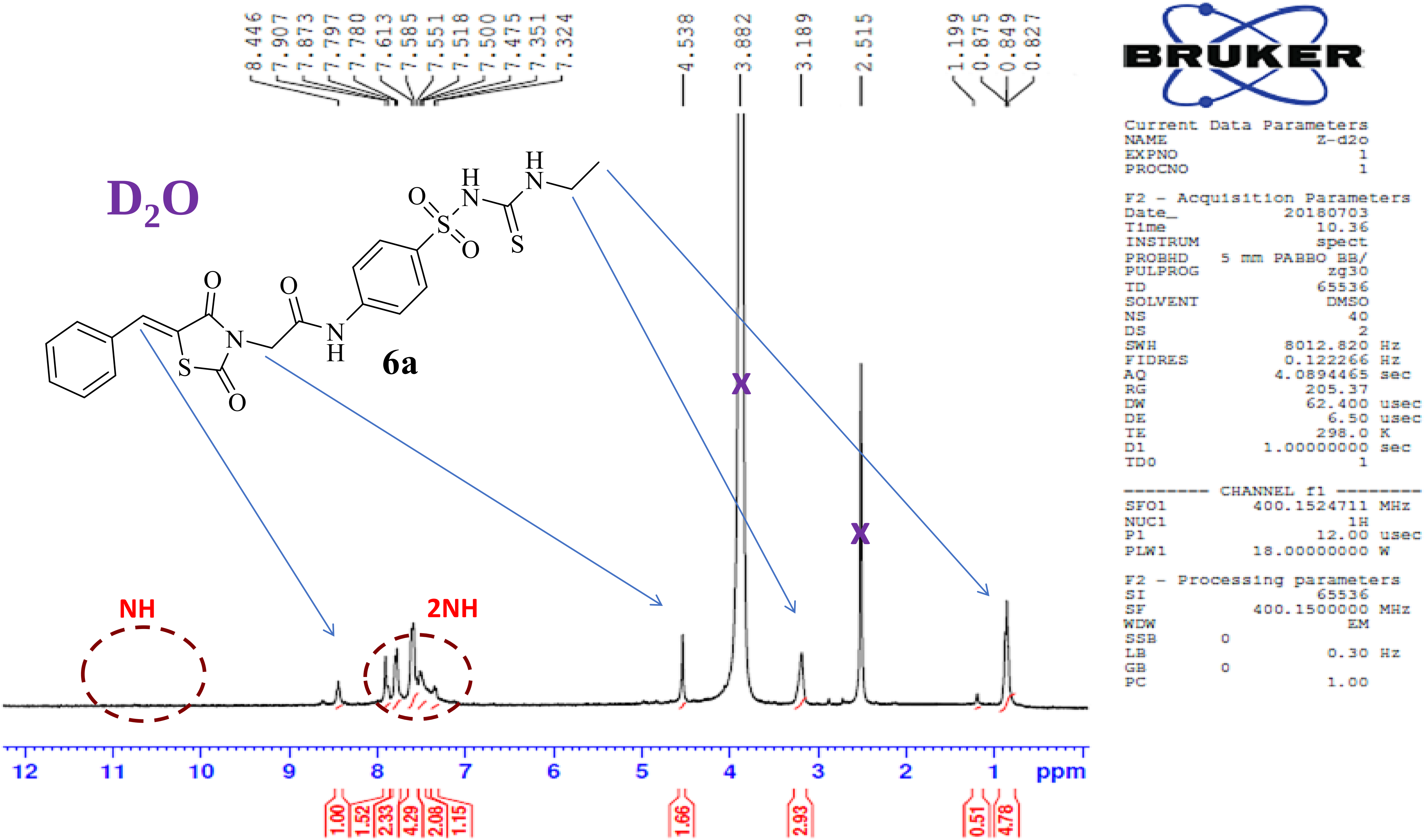
8.446
7.907
7.873
7.797
7.780
7.613
7.585
7.551
7.518
7.500
7.475
7.351
7.324
4.538
3.882
3.189
2.515
1.199
0.875
0.849
0.827
D2O
6a
NH
2NH
X
X
12 11 10 9 8 7 6 5 4 3 2 1 ppm
1.00 1.52 2.33 4.29 2.08 1.15 1.66 2.93 0.51 4.78
BRUKER
Current Data Parameters
NAME Z-d2o
EXPNO 1
PROCNO 1
F2 - Acquisition Parameters
Date_ 20180703
Time 10.36
INSTRUM spect
PROBHD 5 mm PABBO BB/
PULPROG zg30
TD 65536
SOLVENT DMSO
NS 40
DS 2
SWH 8012.820 Hz
FIDRES 0.122266 Hz
AQ 4.0894465 sec
RG 205.37
DW 62.400 usec
DE 6.50 usec
TE 298.0 K
D1 1.00000000 sec
TD0 1
======== CHANNEL f1 ========
SFO1 400.1524711 MHz
NUC1 1H
P1 12.00 usec
PLW1 18.00000000 W
F2 - Processing parameters
SI 65536
SF 400.1500000 MHz
WDW EM
SSB 0
LB 0.30 Hz
GB 0
PC 1.00

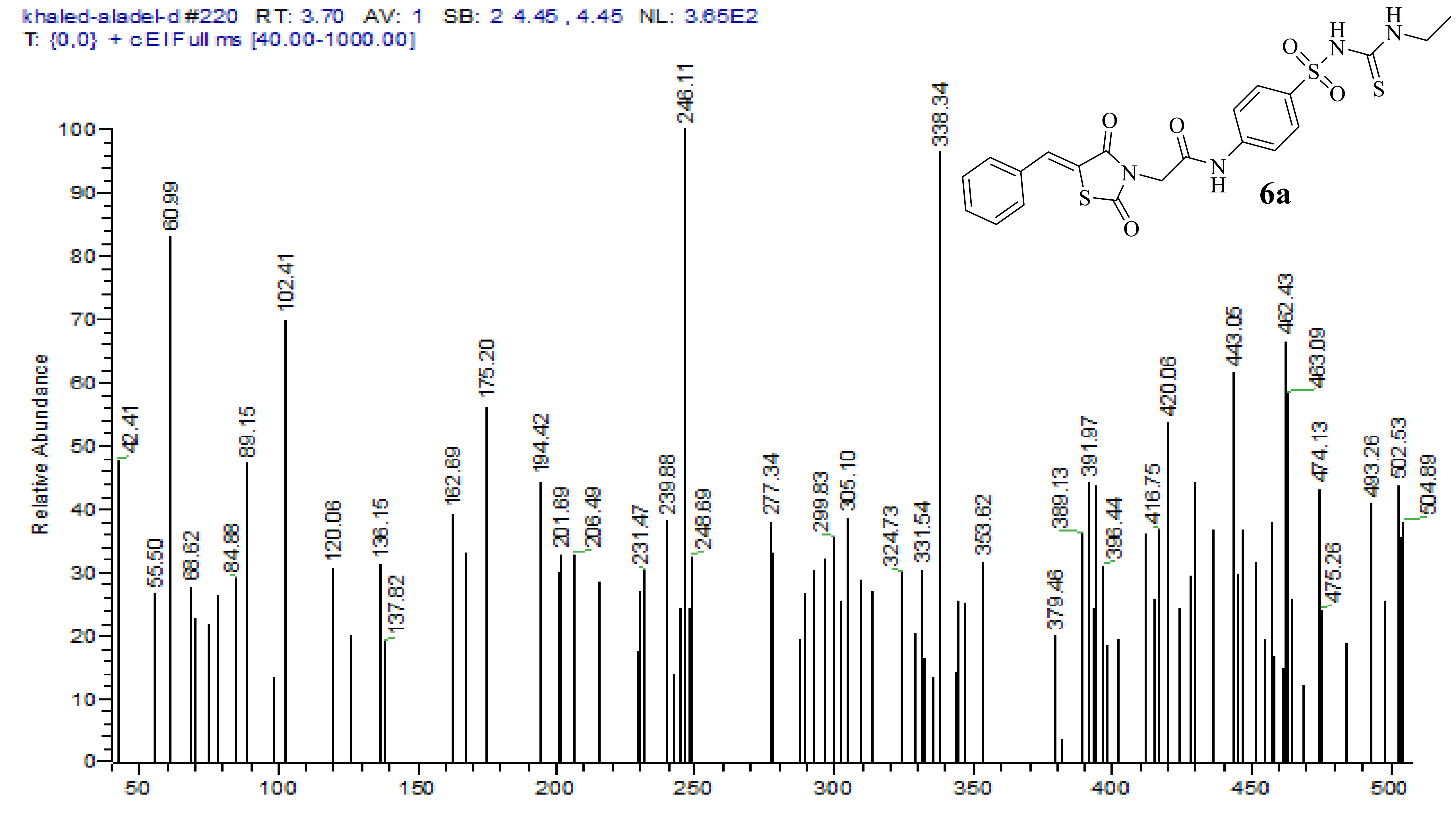
khaled-aladel-d #220 RT: 3.70 AV: 1 SB: 2 4.45 , 4.45 NL: 3.65E2
T: {0,0} + c EI Full ms [40.00-1000.00]
Relative Abundance
42.41
55.50
60.99
68.62
84.88
89.15
102.41
120.06
136.15
137.82
162.69
175.20
194.42
201.69
206.49
231.47
239.88
246.11
248.69
277.34
299.83
305.10
324.73
331.54
338.34
353.62
379.46
389.13
391.97
396.44
416.75
420.06
443.05
462.43
463.09
474.13
475.26
493.26
502.53
504.89
6a

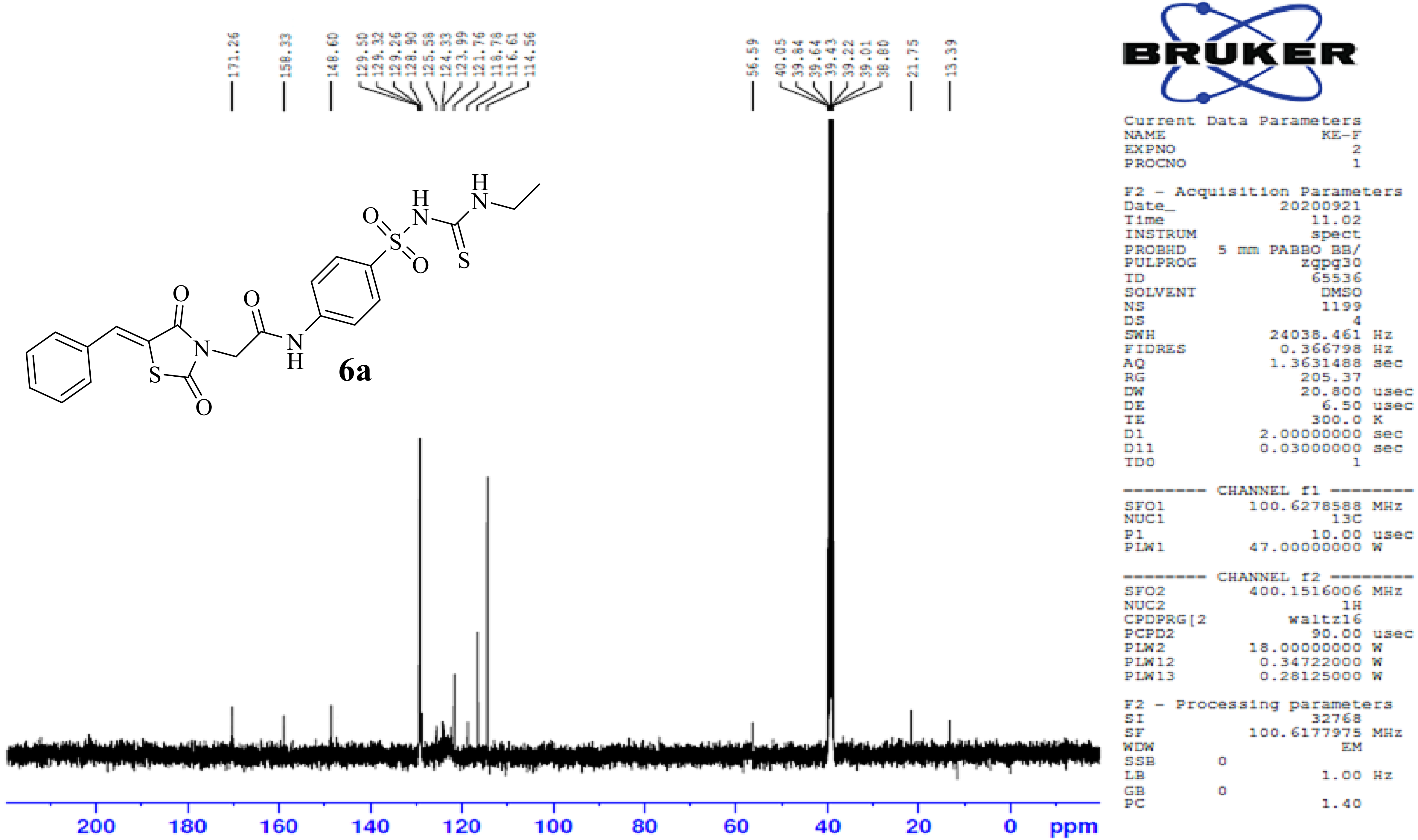
171.26
158.33
148.60
129.50
129.32
129.26
128.90
125.58
124.33
123.99
121.76
118.78
116.61
114.56
56.59
40.05
39.84
39.64
39.43
39.22
39.01
38.80
21.75
13.39
6a
200
180
160
140
120
100
80
60
40
20
0
ppm
BRUKER
Current Data Parameters
NAME KE-F
EXPNO 2
PROCNO 1
F2 - Acquisition Parameters
Date_ 20200921
Time 11.02
INSTRUM spect
PROBHD 5 mm PABBO BB/
PULPROG zgpg30
TD 65536
SOLVENT DMSO
NS 1199
DS 4
SWH 24038.461 Hz
FIDRES 0.366798 Hz
AQ 1.3631488 sec
RG 205.37
DW 20.800 usec
DE 6.50 usec
TE 300.0 K
D1 2.00000000 sec
D11 0.03000000 sec
TD0 1
======== CHANNEL f1 ========
SFO1 100.6278588 MHz
NUC1 13C
P1 10.00 usec
PLW1 47.00000000 W
======== CHANNEL f2 ========
SFO2 400.1516006 MHz
NUC2 1H
CPDPRG[2 waltz16
PCPD2 90.00 usec
PLW2 18.00000000 W
PLW12 0.34722000 W
PLW13 0.28125000 W
F2 - Processing parameters
SI 32768
SF 100.6177975 MHz
WDW EM
SSB 0
LB 1.00 Hz
GB 0
PC 1.40

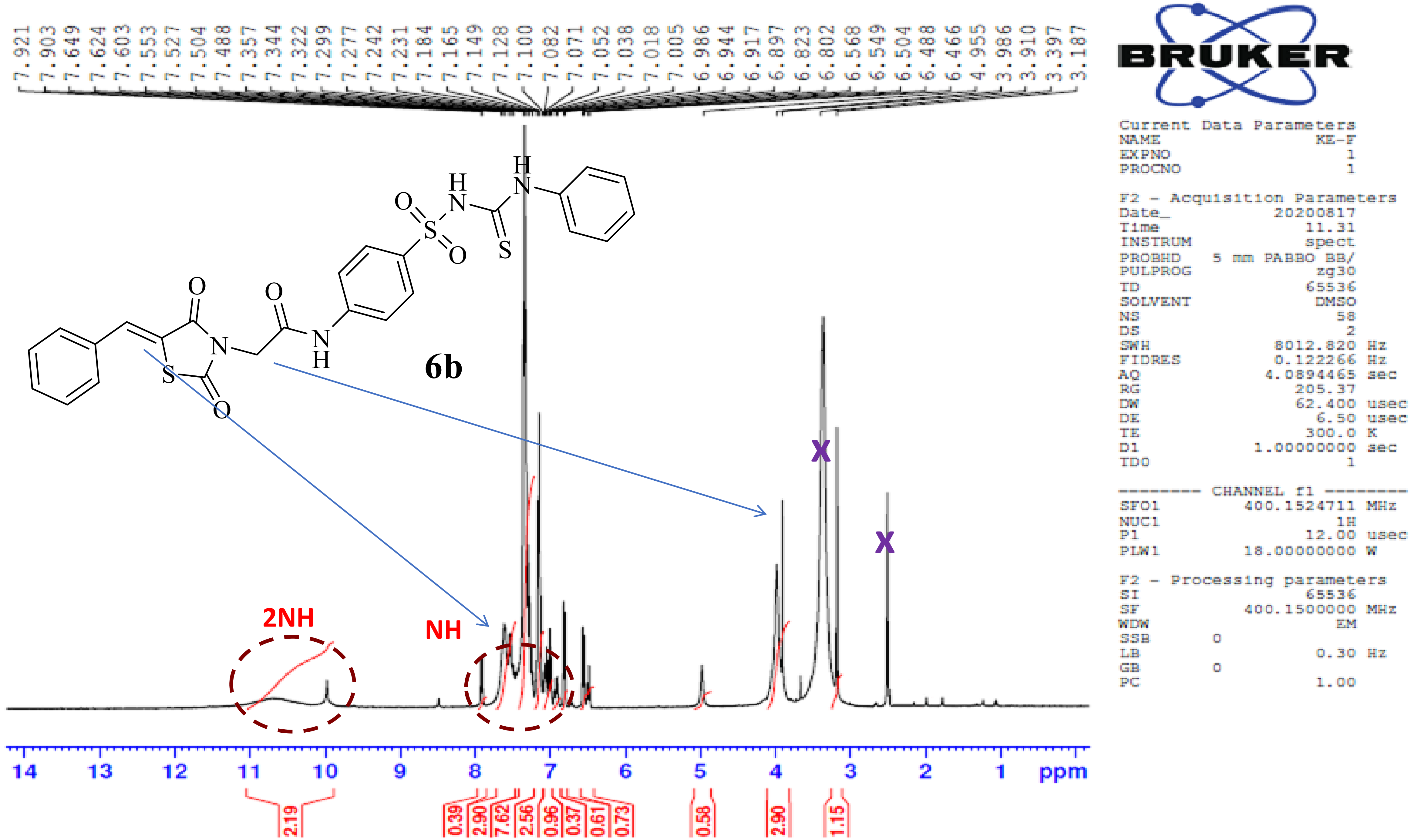

7.921
7.903
7.649
7.624
7.603
7.553
7.527
7.504
7.488
7.357
7.344
7.322
7.299
7.277
7.242
7.231
7.184
7.165
7.149
7.128
7.100
7.082
7.071
7.052
7.038
7.018
7.005
6.986
6.944
6.917
6.897
6.823
6.802
6.568
6.549
6.504
6.488
6.466
4.955
3.986
3.910
3.397
3.187
6b
2NH
NH
X
X
14 13 12 11 10 9 8 7 6 5 4 3 2 1 ppm
2.19
0.39
2.90
7.62
2.56
0.96
0.37
0.61
0.73
0.58
2.90
1.15
BRUKER
Current Data Parameters
NAME KE-F
EXPNO 1
PROCNO 1
F2 - Acquisition Parameters
Date_ 20200817
Time 11.31
INSTRUM spect
PROBHD 5 mm PABBO BB/
PULPROG zg30
TD 65536
SOLVENT DMSO
NS 58
DS 2
SWH 8012.820 Hz
FIDRES 0.122266 Hz
AQ 4.0894465 sec
RG 205.37
DW 62.400 usec
DE 6.50 usec
TE 300.0 K
D1 1.00000000 sec
TD0 1
======== CHANNEL f1 ========
SFO1 400.1524711 MHz
NUC1 1H
P1 12.00 usec
PLW1 18.00000000 W
F2 - Processing parameters
SI 65536
SF 400.1500000 MHz
WDW EM
SSB 0
LB 0.30 Hz
GB 0
PC 1.00

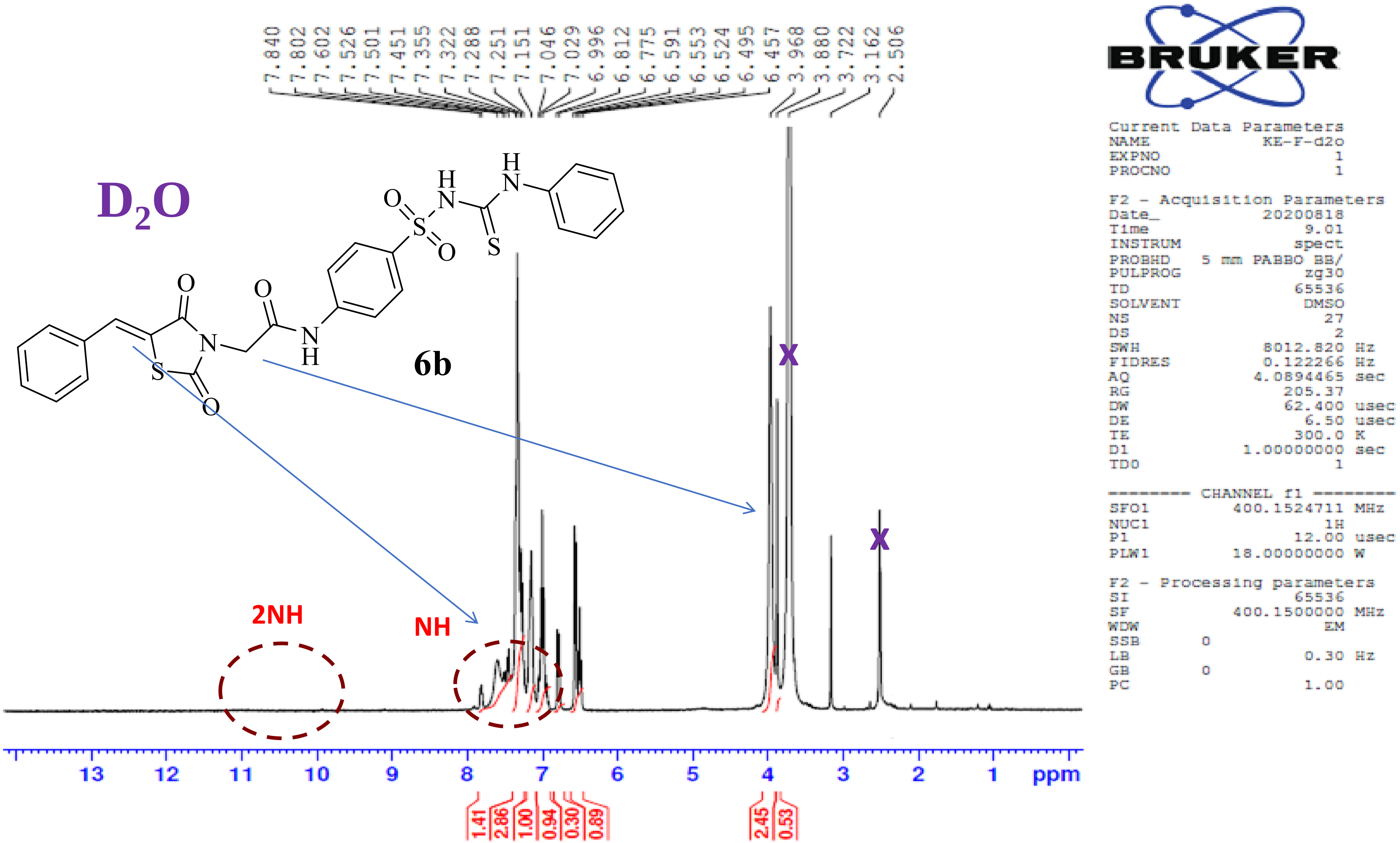

D$_2$O
6b
2NH
NH
X
X
7.840 7.802 7.602 7.526 7.501 7.451 7.355 7.322 7.288 7.251 7.151 7.046 7.029 6.996 6.812 6.775 6.591 6.553 6.524 6.495 6.457 3.968 3.880 3.722 3.162 2.506
13 12 11 10 9 8 7 6 5 4 3 2 1 ppm
1.41 2.86 1.00 0.94 0.30 0.89 2.45 0.53
BRUKER
Current Data Parameters
NAME KE-F-d2o
EXPNO 1
PROCNO 1
F2 - Acquisition Parameters
Date_ 20200818
Time 9.01
INSTRUM spect
PROBHD 5 mm PABBO BB/
PULPROG zg30
TD 65536
SOLVENT DMSO
NS 27
DS 2
SWH 8012.820 Hz
FIDRES 0.122266 Hz
AQ 4.0894465 sec
RG 205.37
DW 62.400 usec
DE 6.50 usec
TE 300.0 K
D1 1.00000000 sec
TD0 1
======== CHANNEL f1 ========
SFO1 400.1524711 MHz
NUC1 1H
P1 12.00 usec
PLW1 18.00000000 W
F2 - Processing parameters
SI 65536
SF 400.1500000 MHz
WDW EM
SSB 0
LB 0.30 Hz
GB 0
PC 1.00

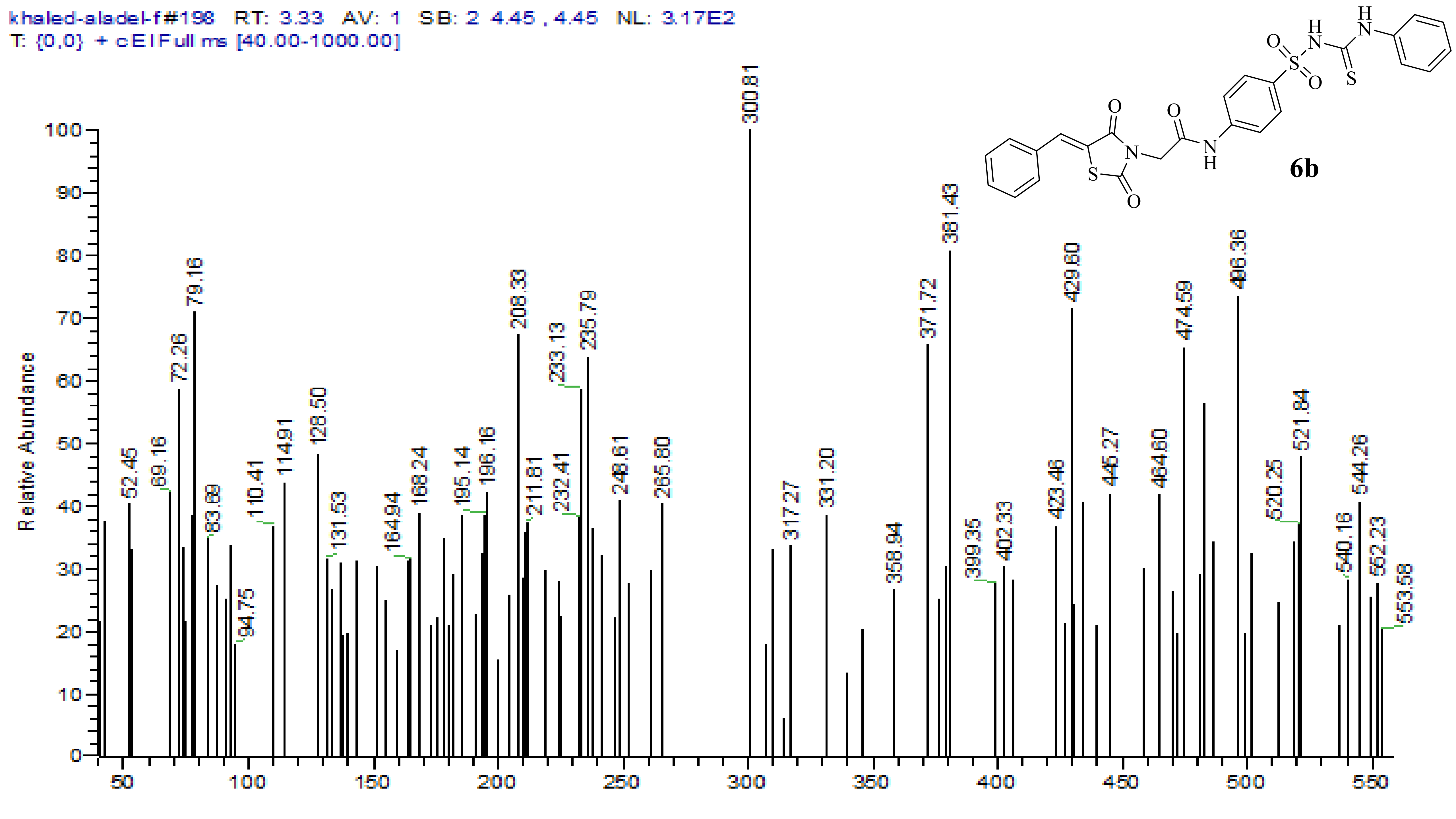
khaled-aladel-f #198 RT: 3.33 AV: 1 SB: 2 4.45 , 4.45 NL: 3.17E2
T: {0,0} + c EI Full ms [40.00-1000.00]
Relative Abundance
6b
300.81
381.43
496.36
429.60
79.16
371.72
474.59
208.33
235.79
233.13
72.26
521.84
128.50
114.91

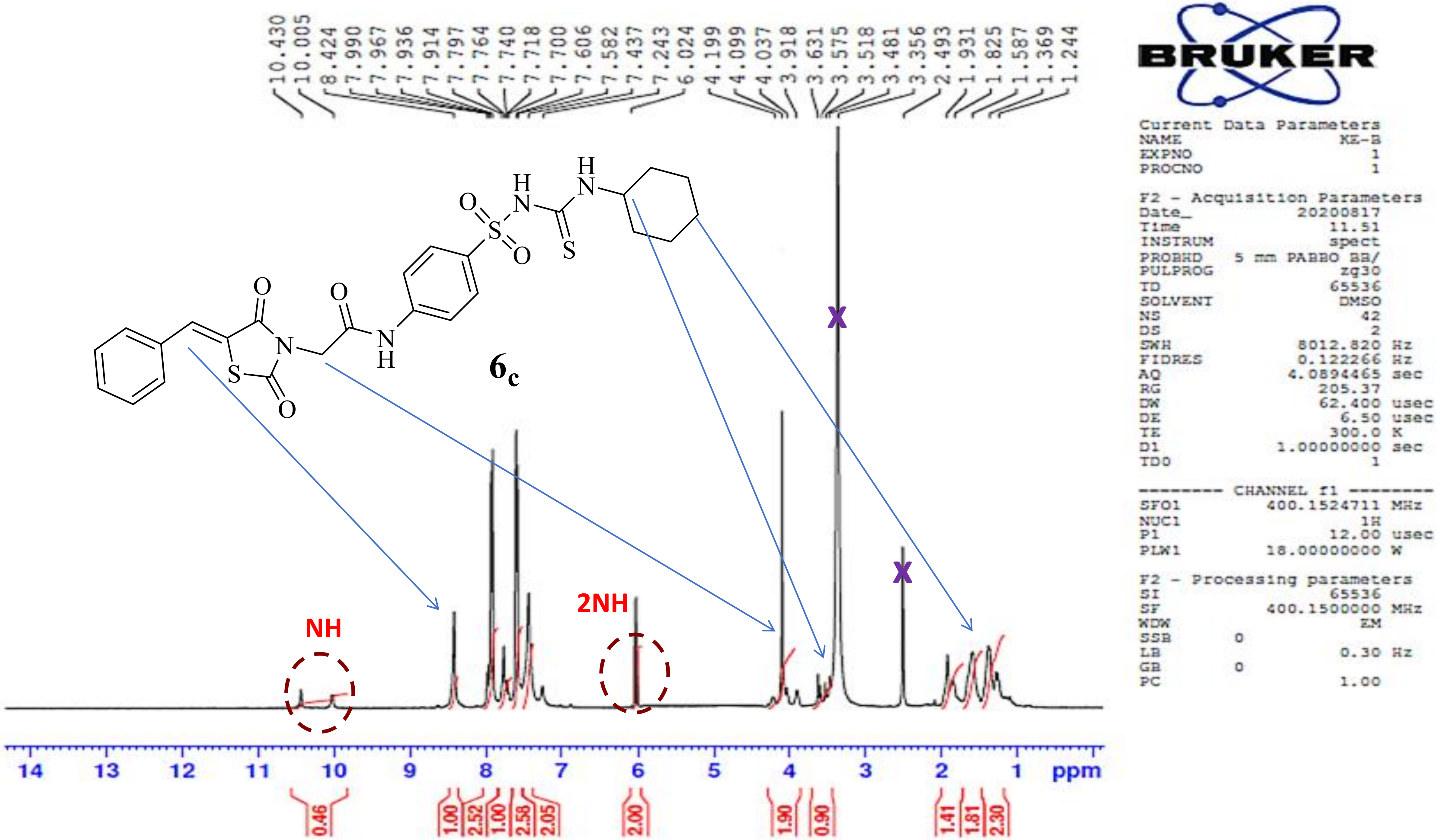
10.430
10.005
8.424
7.990
7.967
7.936
7.914
7.797
7.764
7.740
7.718
7.700
7.606
7.582
7.437
7.243
6.024
4.199
4.099
4.037
3.918
3.631
3.575
3.518
3.481
3.356
2.493
1.931
1.825
1.587
1.369
1.244
6c
NH
2NH
X
X
14 13 12 11 10 9 8 7 6 5 4 3 2 1 ppm
0.46
1.00
2.52
1.00
2.58
2.05
2.00
1.90
0.90
1.41
1.81
2.30
BRUKER
Current Data Parameters
NAME KE-B
EXPNO 1
PROCNO 1
F2 - Acquisition Parameters
Date_ 20200817
Time 11.51
INSTRUM spect
PROBHD 5 mm PABBO BB/
PULPROG zg30
TD 65536
SOLVENT DMSO
NS 42
DS 2
SWH 8012.820 Hz
FIDRES 0.122266 Hz
AQ 4.0894465 sec
RG 205.37
DW 62.400 usec
DE 6.50 usec
TE 300.0 K
D1 1.00000000 sec
TD0 1
======== CHANNEL f1 ========
SFO1 400.1524711 MHz
NUC1 1H
P1 12.00 usec
PLW1 18.00000000 W
F2 - Processing parameters
SI 65536
SF 400.1500000 MHz
WDW EM
SSB 0
LB 0.30 Hz
GB 0
PC 1.00

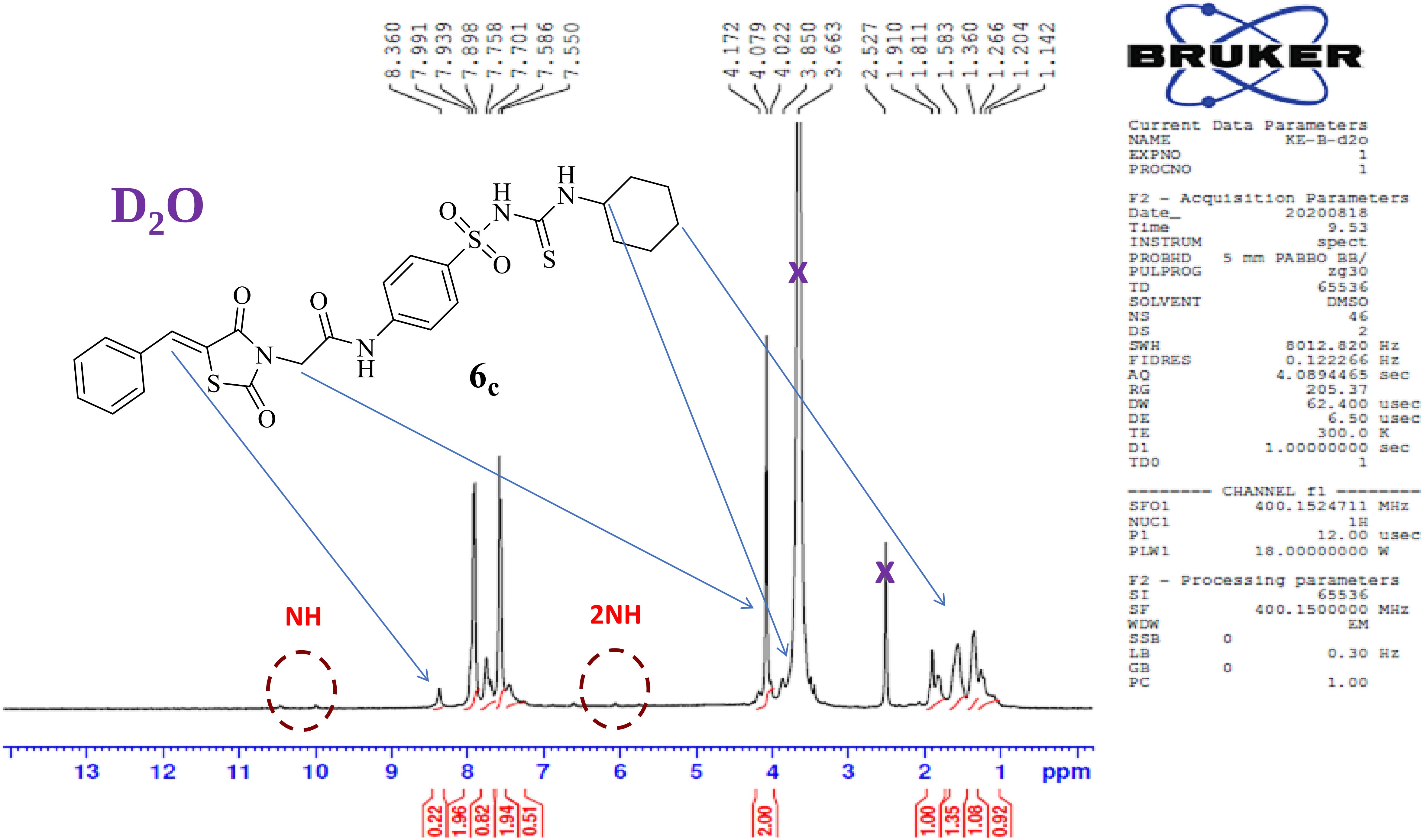
D2O
6c
8.360
7.991
7.939
7.898
7.758
7.701
7.586
7.550
4.172
4.079
4.022
3.850
3.663
2.527
1.910
1.811
1.583
1.360
1.266
1.204
1.142
BRUKER
Current Data Parameters
NAME KE-B-d2o
EXPNO 1
PROCNO 1
F2 - Acquisition Parameters
Date_ 20200818
Time 9.53
INSTRUM spect
PROBHD 5 mm PABBO BB/
PULPROG zg30
TD 65536
SOLVENT DMSO
NS 46
DS 2
SWH 8012.820 Hz
FIDRES 0.122266 Hz
AQ 4.0894465 sec
RG 205.37
DW 62.400 usec
DE 6.50 usec
TE 300.0 K
D1 1.00000000 sec
TD0 1
======== CHANNEL f1 ========
SFO1 400.1524711 MHz
NUC1 1H
P1 12.00 usec
PLW1 18.00000000 W
F2 - Processing parameters
SI 65536
SF 400.1500000 MHz
WDW EM
SSB 0
LB 0.30 Hz
GB 0
PC 1.00
NH
2NH
X
X
13
12
11
10
9
8
7
6
5
4
3
2
1
ppm
0.22
1.96
0.82
1.94
0.51
2.00
1.00
1.35
1.08
0.92

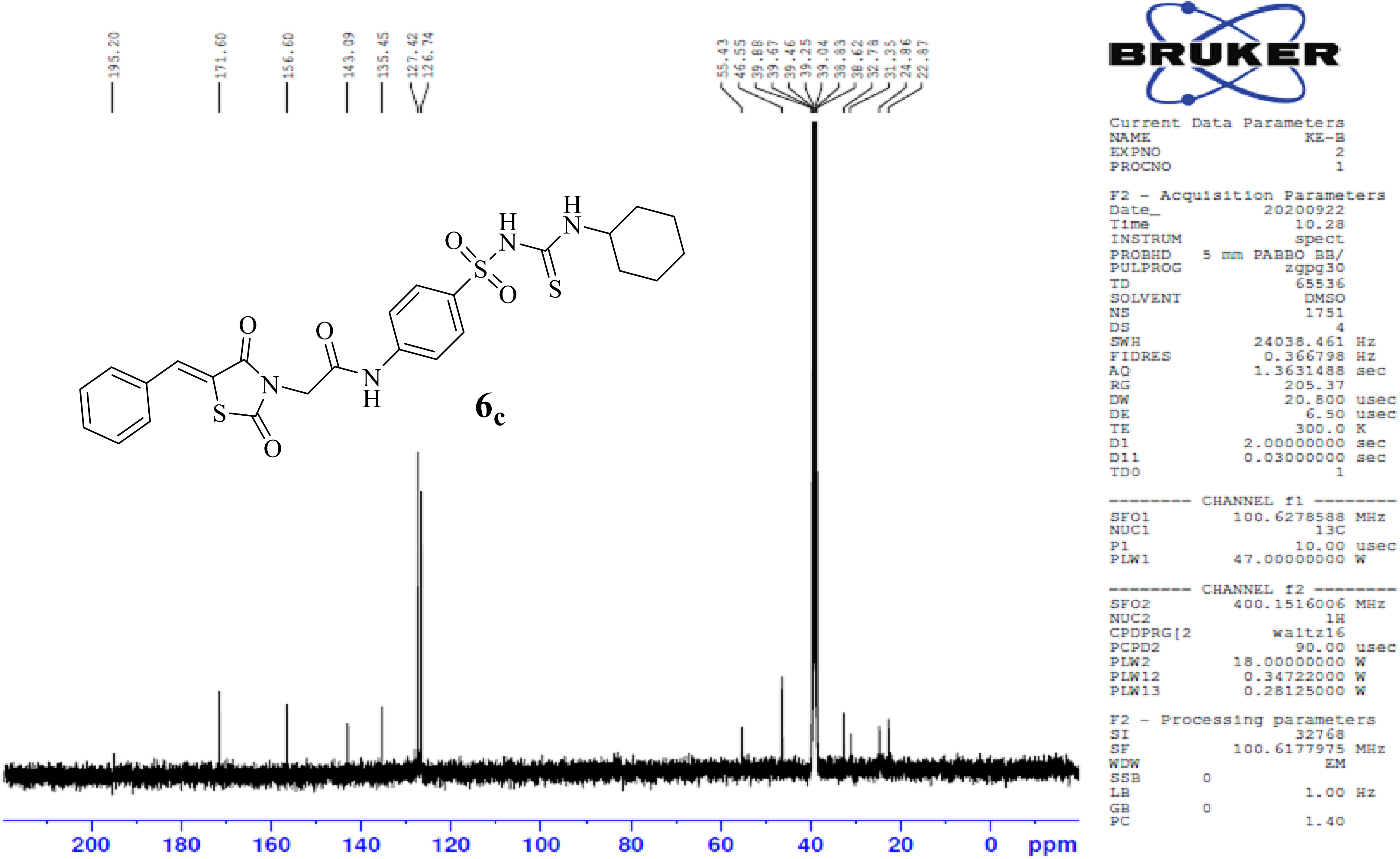
195.20
171.60
156.60
143.09
135.45
127.42
126.74
55.43
46.55
39.88
39.67
39.46
39.25
39.04
38.83
38.62
32.78
31.35
24.96
22.87
6c
200 180 160 140 120 100 80 60 40 20 0 ppm
BRUKER
Current Data Parameters
NAME KE-B
EXPNO 2
PROCNO 1
F2 - Acquisition Parameters
Date_ 20200922
Time 10.28
INSTRUM spect
PROBHD 5 mm PABBO BB/
PULPROG zgpg30
TD 65536
SOLVENT DMSO
NS 1751
DS 4
SWH 24038.461 Hz
FIDRES 0.366798 Hz
AQ 1.3631488 sec
RG 205.37
DW 20.800 usec
DE 6.50 usec
TE 300.0 K
D1 2.00000000 sec
D11 0.03000000 sec
TD0 1
======== CHANNEL f1 ========
SFO1 100.6278588 MHz
NUC1 13C
P1 10.00 usec
PLW1 47.00000000 W
======== CHANNEL f2 ========
SFO2 400.1516006 MHz
NUC2 1H
CPDPRG[2 waltz16
PCPD2 90.00 usec
PLW2 18.00000000 W
PLW12 0.34722000 W
PLW13 0.28125000 W
F2 - Processing parameters
SI 32768
SF 100.6177975 MHz
WDW EM
SSB 0
LB 1.00 Hz
GB 0
PC 1.40

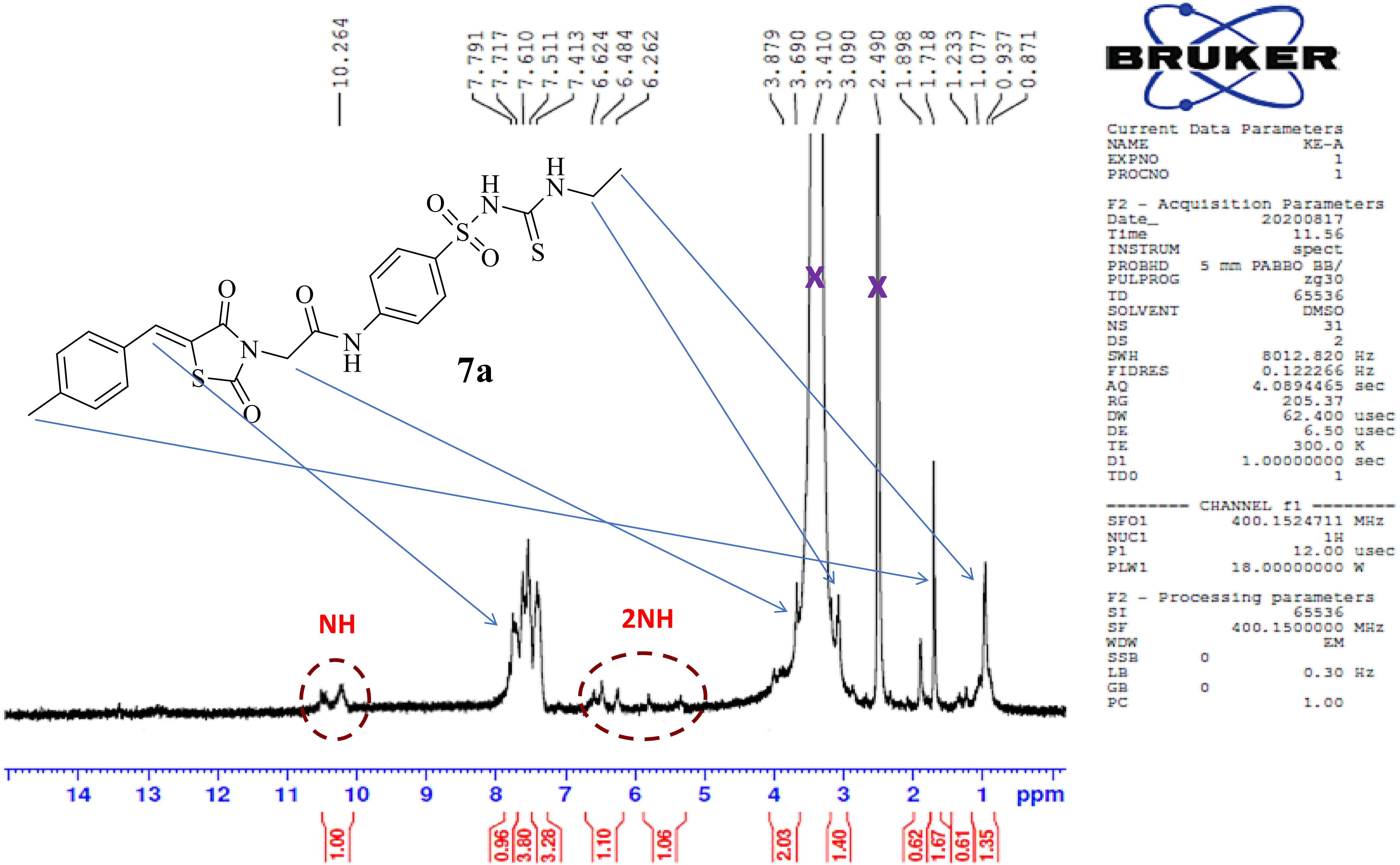

10.264
7.791
7.717
7.610
7.511
7.413
6.624
6.484
6.262
3.879
3.690
3.410
3.090
2.490
1.898
1.718
1.233
1.077
0.937
0.871
7a
NH
2NH
X
X
BRUKER
Current Data Parameters
NAME KE-A
EXPNO 1
PROCNO 1
F2 - Acquisition Parameters
Date_ 20200817
Time 11.56
INSTRUM spect
PROBHD 5 mm PABBO BB/
PULPROG zg30
TD 65536
SOLVENT DMSO
NS 31
DS 2
SWH 8012.820 Hz
FIDRES 0.122266 Hz
AQ 4.0894465 sec
RG 205.37
DW 62.400 usec
DE 6.50 usec
TE 300.0 K
D1 1.00000000 sec
TD0 1
======== CHANNEL f1 ========
SFO1 400.1524711 MHz
NUC1 1H
P1 12.00 usec
PLW1 18.00000000 W
F2 - Processing parameters
SI 65536
SF 400.1500000 MHz
WDW EM
SSB 0
LB 0.30 Hz
GB 0
PC 1.00
14 13 12 11 10 9 8 7 6 5 4 3 2 1 ppm
1.00
0.96
3.80
3.28
1.10
1.06
2.03
1.40
0.62
1.67
0.61
1.35

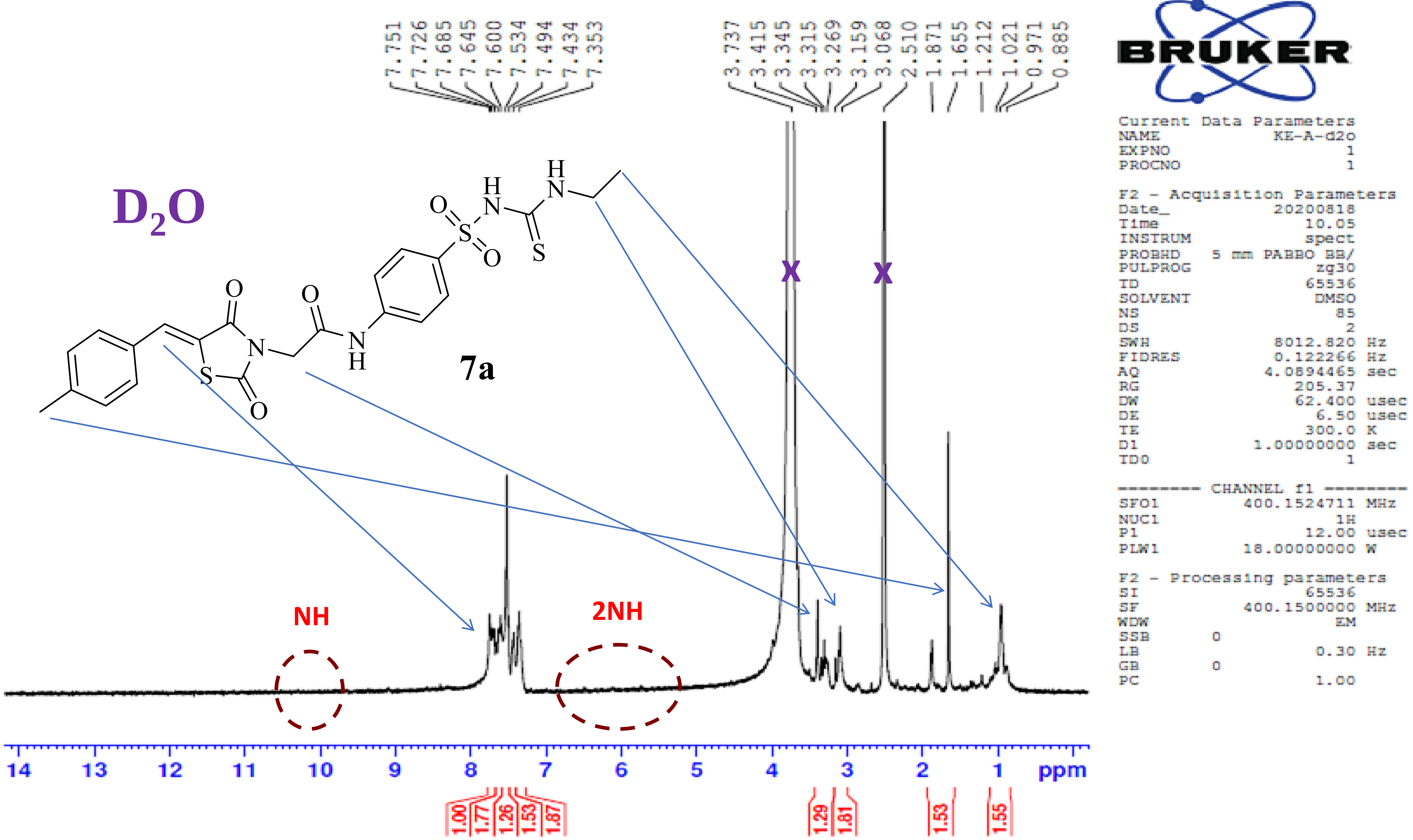

7.751
7.726
7.685
7.645
7.600
7.534
7.494
7.434
7.353
3.737
3.415
3.345
3.315
3.269
3.159
3.068
2.510
1.871
1.655
1.212
1.021
0.971
0.885
D2O
7a
X
X
NH
2NH
14 13 12 11 10 9 8 7 6 5 4 3 2 1 ppm
1.00 1.77 1.26 1.53 1.87
1.29 1.81
1.53
1.55
BRUKER
Current Data Parameters
NAME KE-A-d2o
EXPNO 1
PROCNO 1
F2 - Acquisition Parameters
Date_ 20200818
Time 10.05
INSTRUM spect
PROBHD 5 mm PABBO BB/
PULPROG zg30
TD 65536
SOLVENT DMSO
NS 85
DS 2
SWH 8012.820 Hz
FIDRES 0.122266 Hz
AQ 4.0894465 sec
RG 205.37
DW 62.400 usec
DE 6.50 usec
TE 300.0 K
D1 1.00000000 sec
TD0 1
======== CHANNEL f1 ========
SFO1 400.1524711 MHz
NUC1 1H
P1 12.00 usec
PLW1 18.00000000 W
F2 - Processing parameters
SI 65536
SF 400.1500000 MHz
WDW EM
SSB 0
LB 0.30 Hz
GB 0
PC 1.00

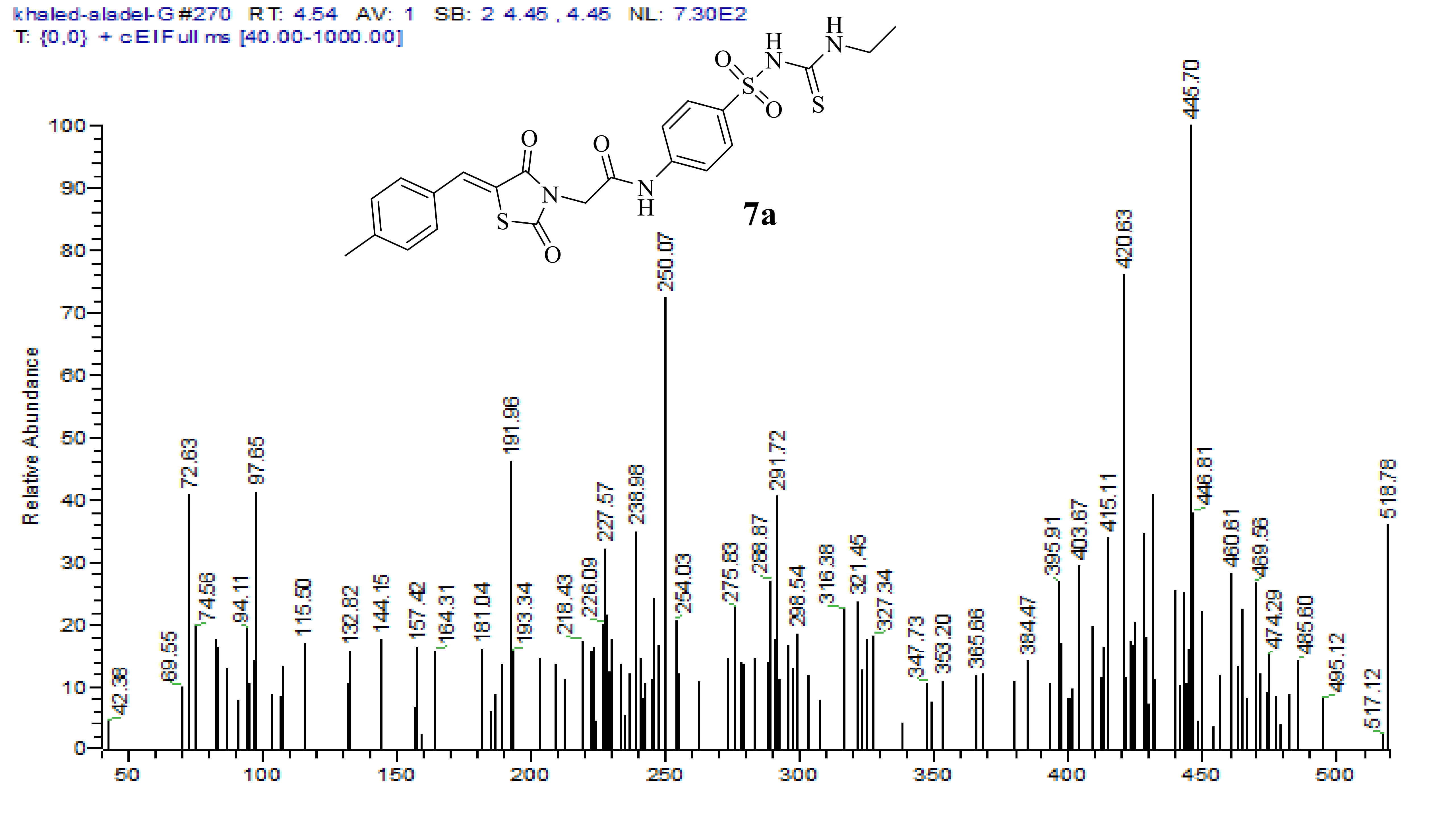
khaled-aladel-G#270 RT: 4.54 AV: 1 SB: 2 4.45 , 4.45 NL: 7.30E2
T: {0,0} + c EI Full ms [40.00-1000.00]
7a
Relative Abundance
42.38
69.55
72.63
74.56
94.11
97.65
115.50
132.82
144.15
157.42
164.31
181.04
191.96
193.34
218.43
226.09
227.57
238.98
250.07
254.03
275.83
288.87
291.72
298.54
316.38
321.45
327.34
347.73
353.20
365.66
384.47
395.91
403.67
415.11
420.63
445.70
446.81
460.61
469.56
474.29
485.60
495.12
517.12
518.78

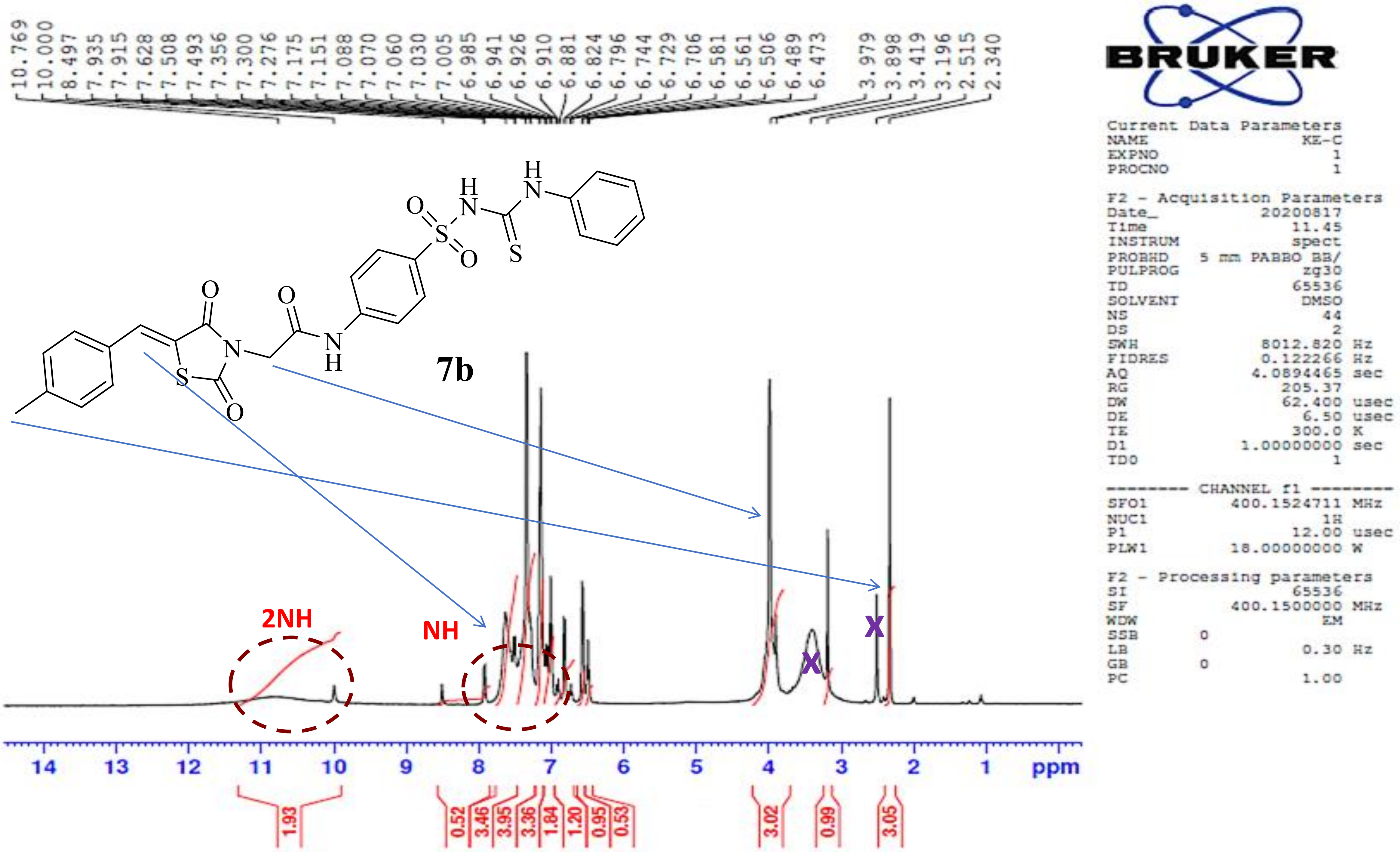
10.769
10.000
8.497
7.935
7.915
7.628
7.508
7.493
7.356
7.300
7.276
7.175
7.151
7.088
7.070
7.060
7.030
7.005
6.985
6.941
6.926
6.910
6.881
6.824
6.796
6.744
6.729
6.706
6.581
6.561
6.506
6.489
6.473
3.979
3.898
3.419
3.196
2.515
2.340
7b
2NH
NH
X
X
14 13 12 11 10 9 8 7 6 5 4 3 2 1 ppm
1.93
0.52
3.46
3.95
3.36
1.84
1.20
0.95
0.53
3.02
0.99
3.05
BRUKER
Current Data Parameters
NAME KE-C
EXPNO 1
PROCNO 1
F2 - Acquisition Parameters
Date_ 20200817
Time 11.45
INSTRUM spect
PROBHD 5 mm PABBO BB/
PULPROG zg30
TD 65536
SOLVENT DMSO
NS 44
DS 2
SWH 8012.820 Hz
FIDRES 0.122266 Hz
AQ 4.0894465 sec
RG 205.37
DW 62.400 usec
DE 6.50 usec
TE 300.0 K
D1 1.00000000 sec
TD0 1
======== CHANNEL f1 ========
SFO1 400.1524711 MHz
NUC1 1H
P1 12.00 usec
PLW1 18.00000000 W
F2 - Processing parameters
SI 65536
SF 400.1500000 MHz
WDW EM
SSB 0
LB 0.30 Hz
GB 0
PC 1.00

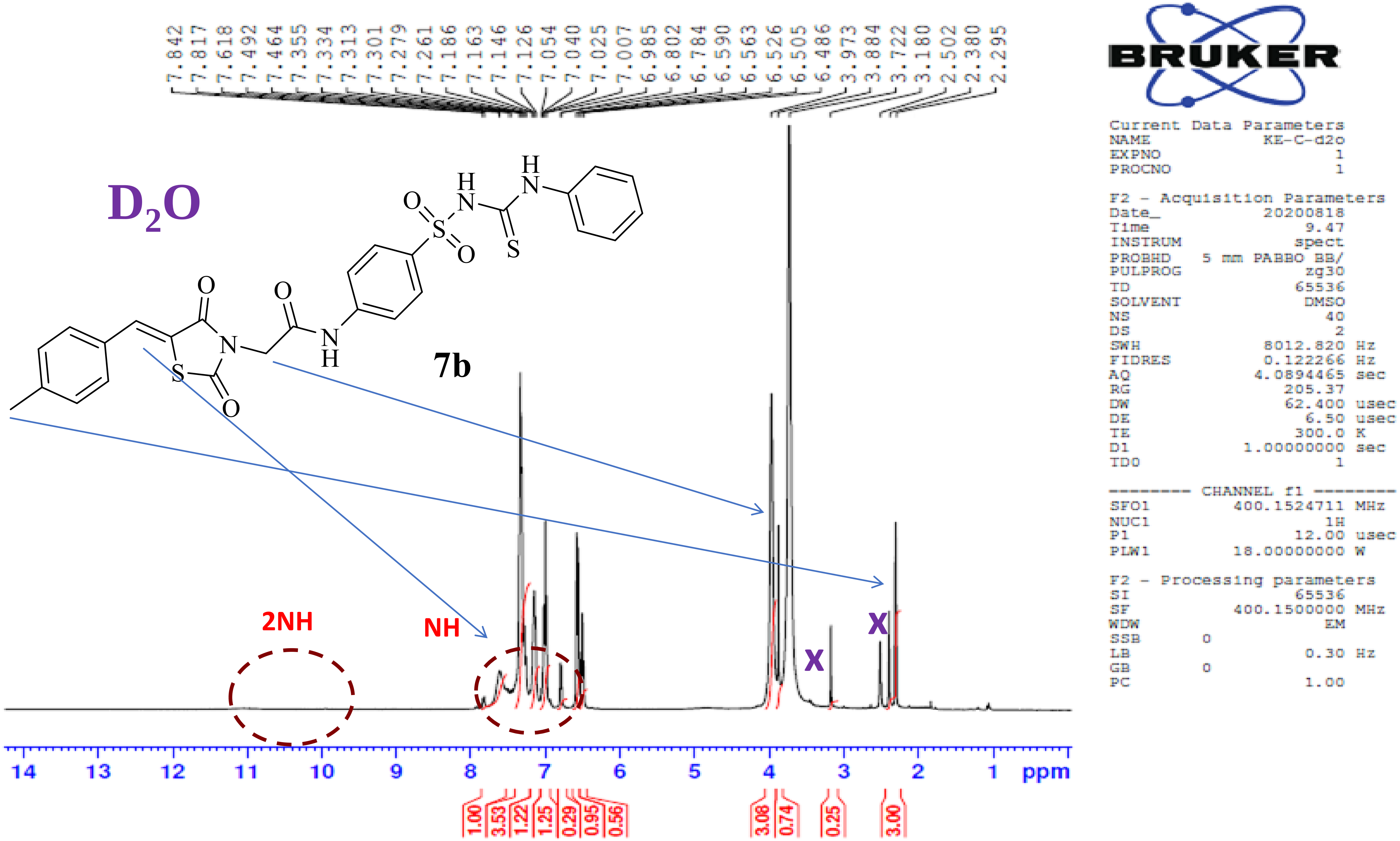
7.842 7.817 7.618 7.492 7.464 7.355 7.334 7.313 7.301 7.279 7.261 7.186 7.163 7.146 7.126 7.054 7.040 7.025 7.007 6.985 6.802 6.784 6.590 6.563 6.526 6.505 6.486 3.973 3.884 3.722 3.180 2.502 2.380 2.295
D2O
7b
2NH
NH
X
X
14 13 12 11 10 9 8 7 6 5 4 3 2 1 ppm
1.00 3.53 1.22 1.25 0.29 0.95 0.56 3.08 0.74 0.25 3.00
BRUKER
Current Data Parameters
NAME KE-C-d2o
EXPNO 1
PROCNO 1
F2 - Acquisition Parameters
Date_ 20200818
Time 9.47
INSTRUM spect
PROBHD 5 mm PABBO BB/
PULPROG zg30
TD 65536
SOLVENT DMSO
NS 40
DS 2
SWH 8012.820 Hz
FIDRES 0.122266 Hz
AQ 4.0894465 sec
RG 205.37
DW 62.400 usec
DE 6.50 usec
TE 300.0 K
D1 1.00000000 sec
TD0 1
CHANNEL f1
SFO1 400.1524711 MHz
NUC1 1H
P1 12.00 usec
PLW1 18.00000000 W
F2 - Processing parameters
SI 65536
SF 400.1500000 MHz
WDW EM
SSB 0
LB 0.30 Hz
GB 0
PC 1.00

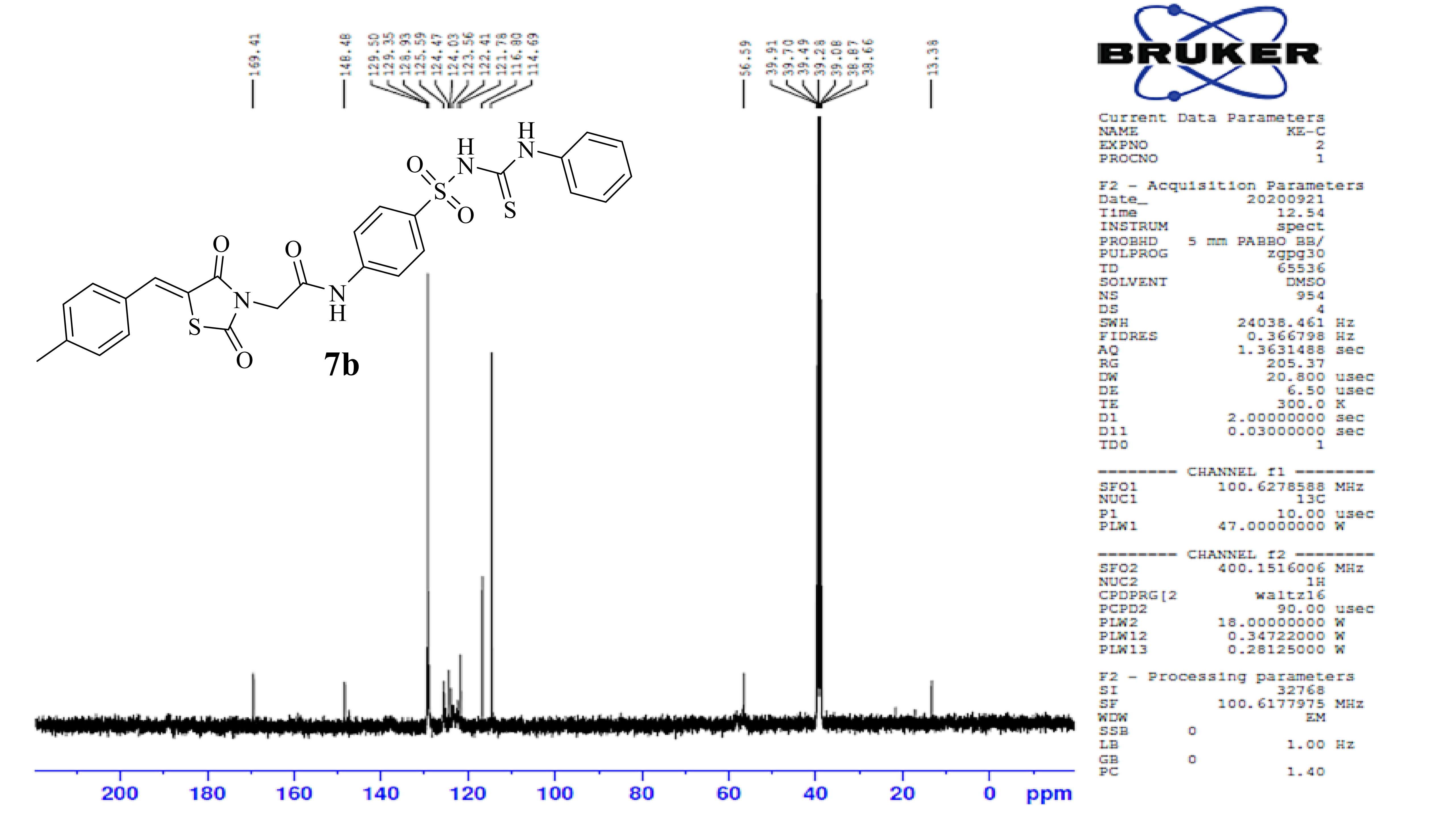
169.41
148.48
129.50
129.35
128.93
125.59
124.47
124.03
123.56
122.41
121.78
116.80
114.69
56.59
39.91
39.70
39.49
39.28
39.08
38.87
38.66
13.38
7b
200
180
160
140
120
100
80
60
40
20
0
ppm
BRUKER
Current Data Parameters
NAME KE-C
EXPNO 2
PROCNO 1
F2 - Acquisition Parameters
Date_ 20200921
Time 12.54
INSTRUM spect
PROBHD 5 mm PABBO BB/
PULPROG zgpg30
TD 65536
SOLVENT DMSO
NS 954
DS 4
SWH 24038.461 Hz
FIDRES 0.366798 Hz
AQ 1.3631488 sec
RG 205.37
DW 20.800 usec
DE 6.50 usec
TE 300.0 K
D1 2.00000000 sec
D11 0.03000000 sec
TD0 1
======== CHANNEL f1 ========
SFO1 100.6278588 MHz
NUC1 13C
P1 10.00 usec
PLW1 47.00000000 W
======== CHANNEL f2 ========
SFO2 400.1516006 MHz
NUC2 1H
CPDPRG[2 waltz16
PCPD2 90.00 usec
PLW2 18.00000000 W
PLW12 0.34722000 W
PLW13 0.28125000 W
F2 - Processing parameters
SI 32768
SF 100.6177975 MHz
WDW EM
SSB 0
LB 1.00 Hz
GB 0
PC 1.40

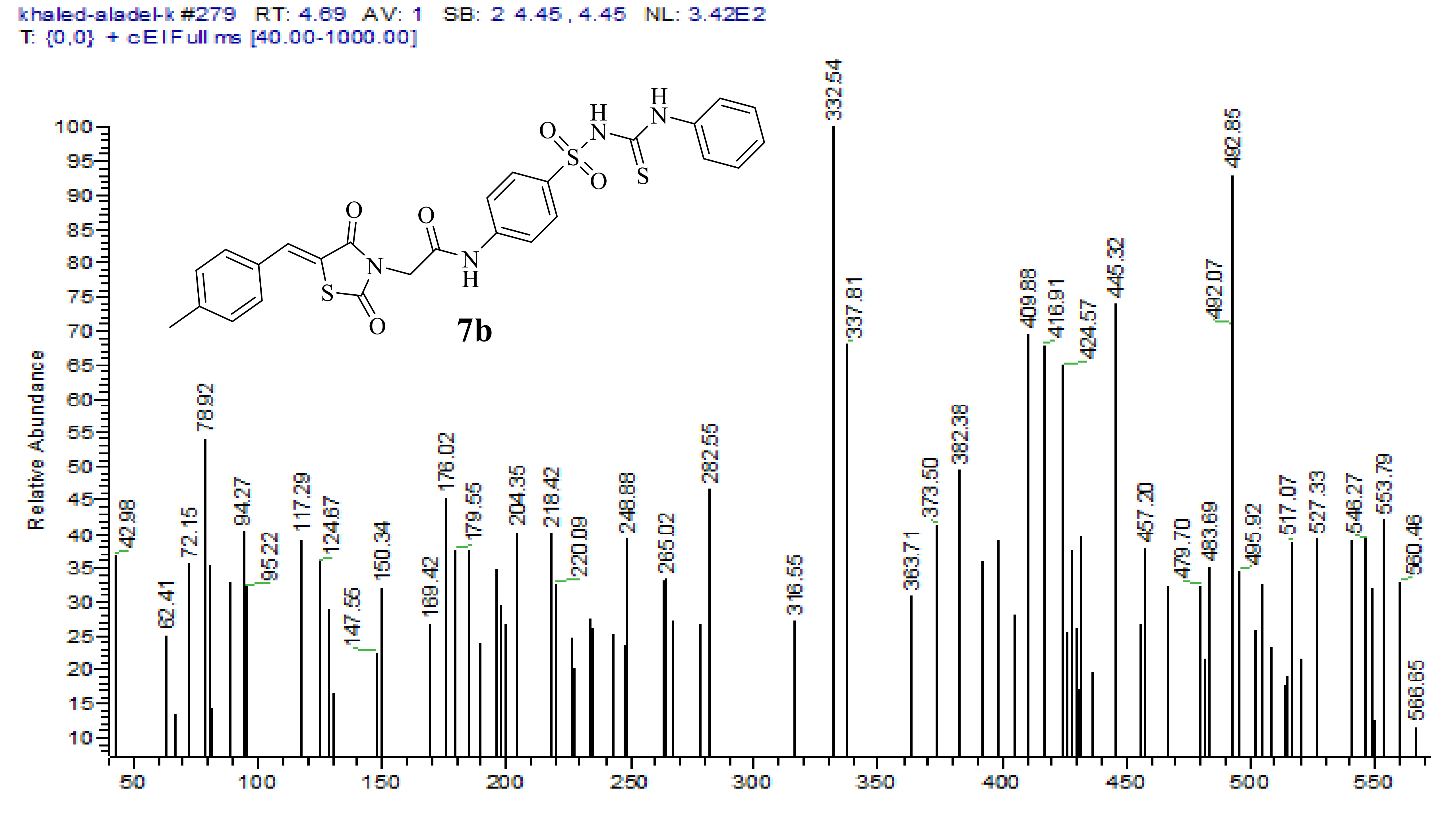
khaled-aladel-k #279 RT: 4.69 AV: 1 SB: 2 4.45 , 4.45 NL: 3.42E2
T: {0,0} + c EI Full ms [40.00-1000.00]
Relative Abundance
7b
332.54
492.85
445.32
492.07
409.88
416.91
424.57
337.81
78.92
282.55
176.02
382.38
373.50
553.79

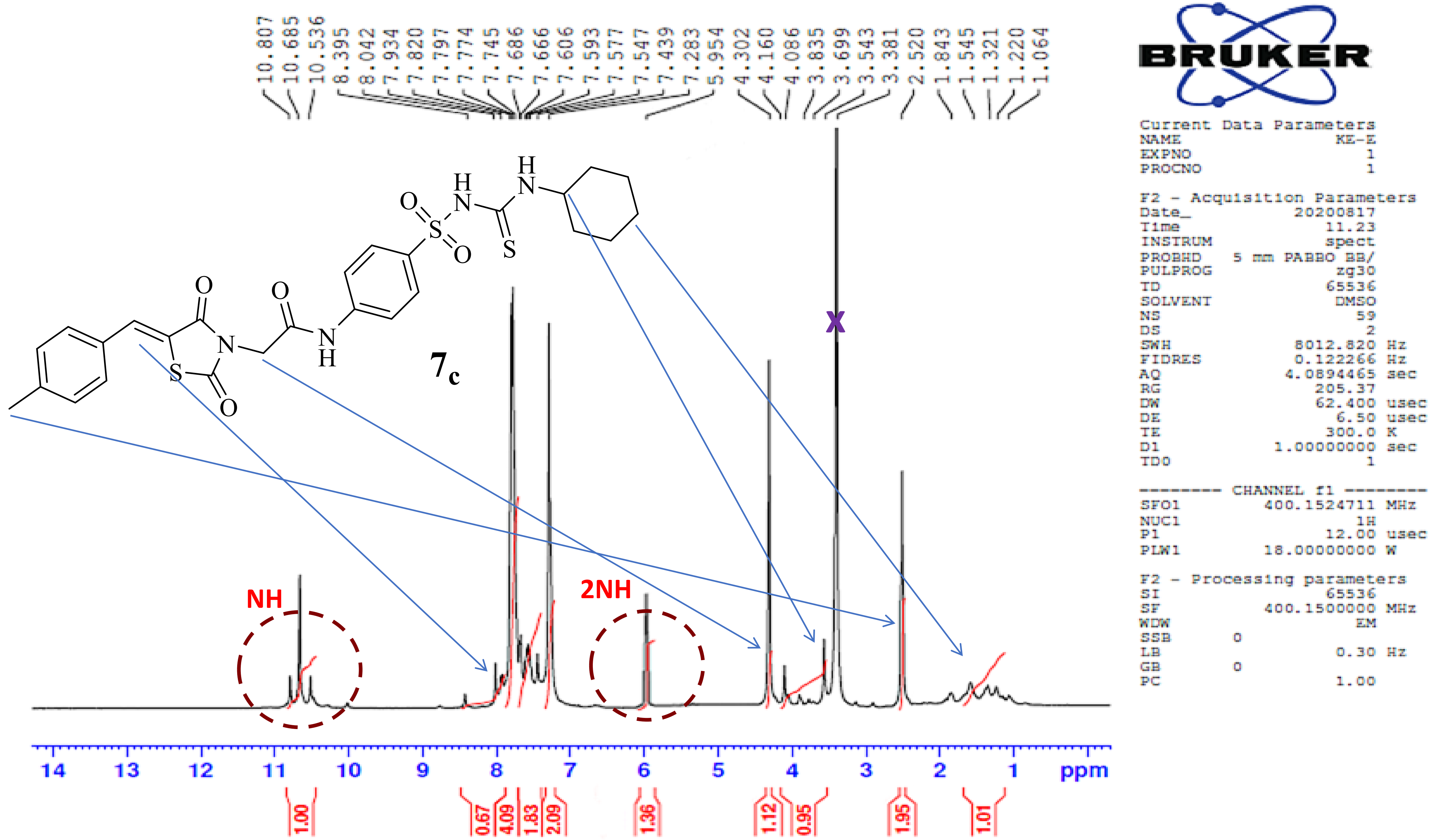
10.807 10.685 10.536 8.395 8.042 7.934 7.820 7.797 7.774 7.745 7.686 7.666 7.606 7.593 7.577 7.547 7.439 7.283 5.954 4.302 4.160 4.086 3.835 3.699 3.543 3.381 2.520 1.843 1.545 1.321 1.220 1.064
7c
NH
2NH
X
14 13 12 11 10 9 8 7 6 5 4 3 2 1 ppm
1.00 0.67 4.09 1.83 2.09 1.36 1.12 0.95 1.95 1.01
BRUKER
Current Data Parameters
NAME KE-E
EXPNO 1
PROCNO 1
F2 - Acquisition Parameters
Date_ 20200817
Time 11.23
INSTRUM spect
PROBHD 5 mm PABBO BB/
PULPROG zg30
TD 65536
SOLVENT DMSO
NS 59
DS 2
SWH 8012.820 Hz
FIDRES 0.122266 Hz
AQ 4.0894465 sec
RG 205.37
DW 62.400 usec
DE 6.50 usec
TE 300.0 K
D1 1.00000000 sec
TD0 1
======== CHANNEL f1 ========
SFO1 400.1524711 MHz
NUC1 1H
P1 12.00 usec
PLW1 18.00000000 W
F2 - Processing parameters
SI 65536
SF 400.1500000 MHz
WDW EM
SSB 0
LB 0.30 Hz
GB 0
PC 1.00

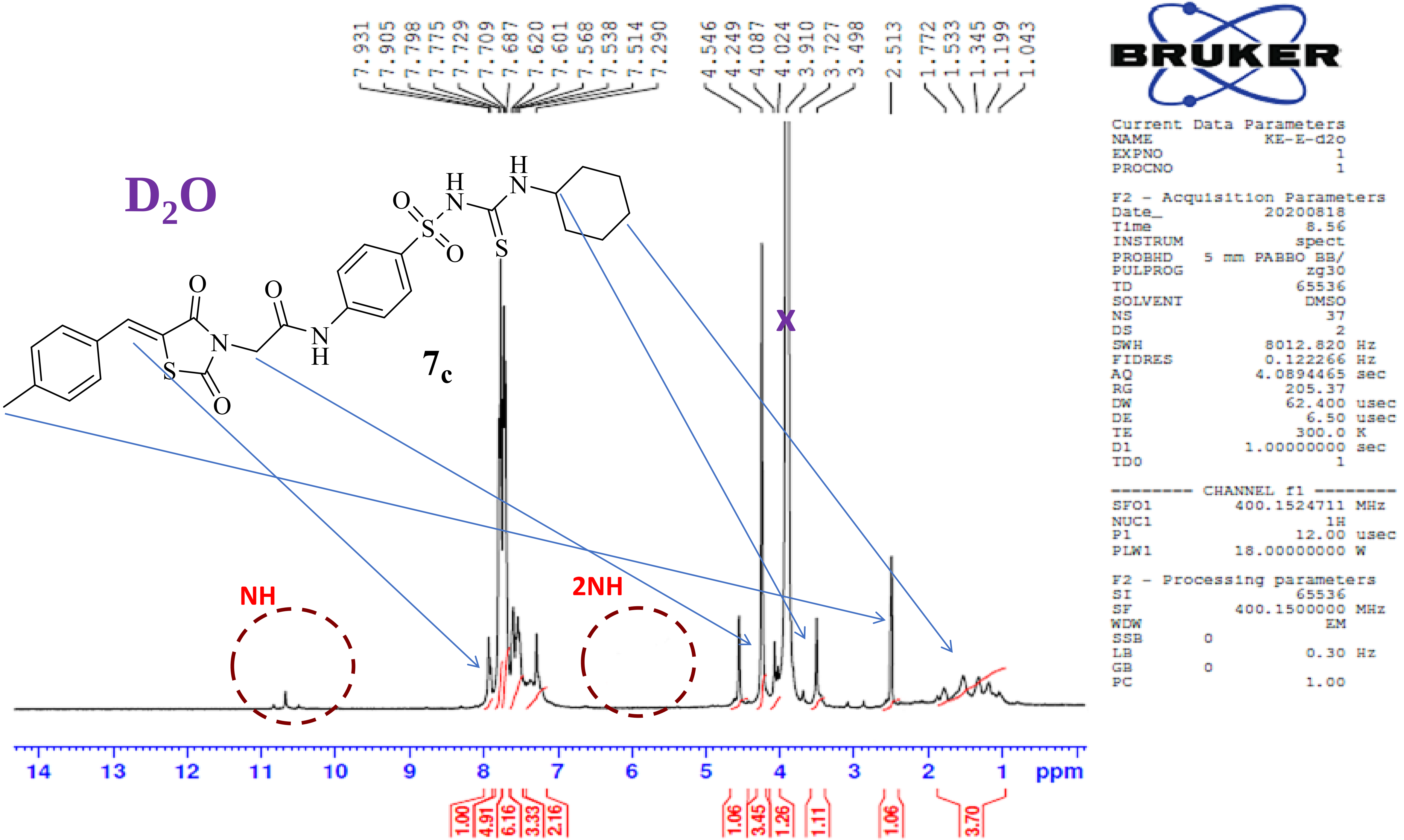

D2O
7c
NH
2NH
X
7.931 7.905 7.798 7.775 7.729 7.709 7.687 7.620 7.601 7.568 7.538 7.514 7.290
4.546 4.249 4.087 4.024 3.910 3.727 3.498
2.513
1.772 1.533 1.345 1.199 1.043
14 13 12 11 10 9 8 7 6 5 4 3 2 1 ppm
1.00 4.91 6.16 3.33 2.16 1.06 3.45 1.26 1.11 1.06 3.70
BRUKER
Current Data Parameters
NAME KE-E-d2o
EXPNO 1
PROCNO 1
F2 - Acquisition Parameters
Date_ 20200818
Time 8.56
INSTRUM spect
PROBHD 5 mm PABBO BB/
PULPROG zg30
TD 65536
SOLVENT DMSO
NS 37
DS 2
SWH 8012.820 Hz
FIDRES 0.122266 Hz
AQ 4.0894465 sec
RG 205.37
DW 62.400 usec
DE 6.50 usec
TE 300.0 K
D1 1.00000000 sec
TD0 1
======== CHANNEL f1 ========
SFO1 400.1524711 MHz
NUC1 1H
P1 12.00 usec
PLW1 18.00000000 W
F2 - Processing parameters
SI 65536
SF 400.1500000 MHz
WDW EM
SSB 0
LB 0.30 Hz
GB 0
PC 1.00

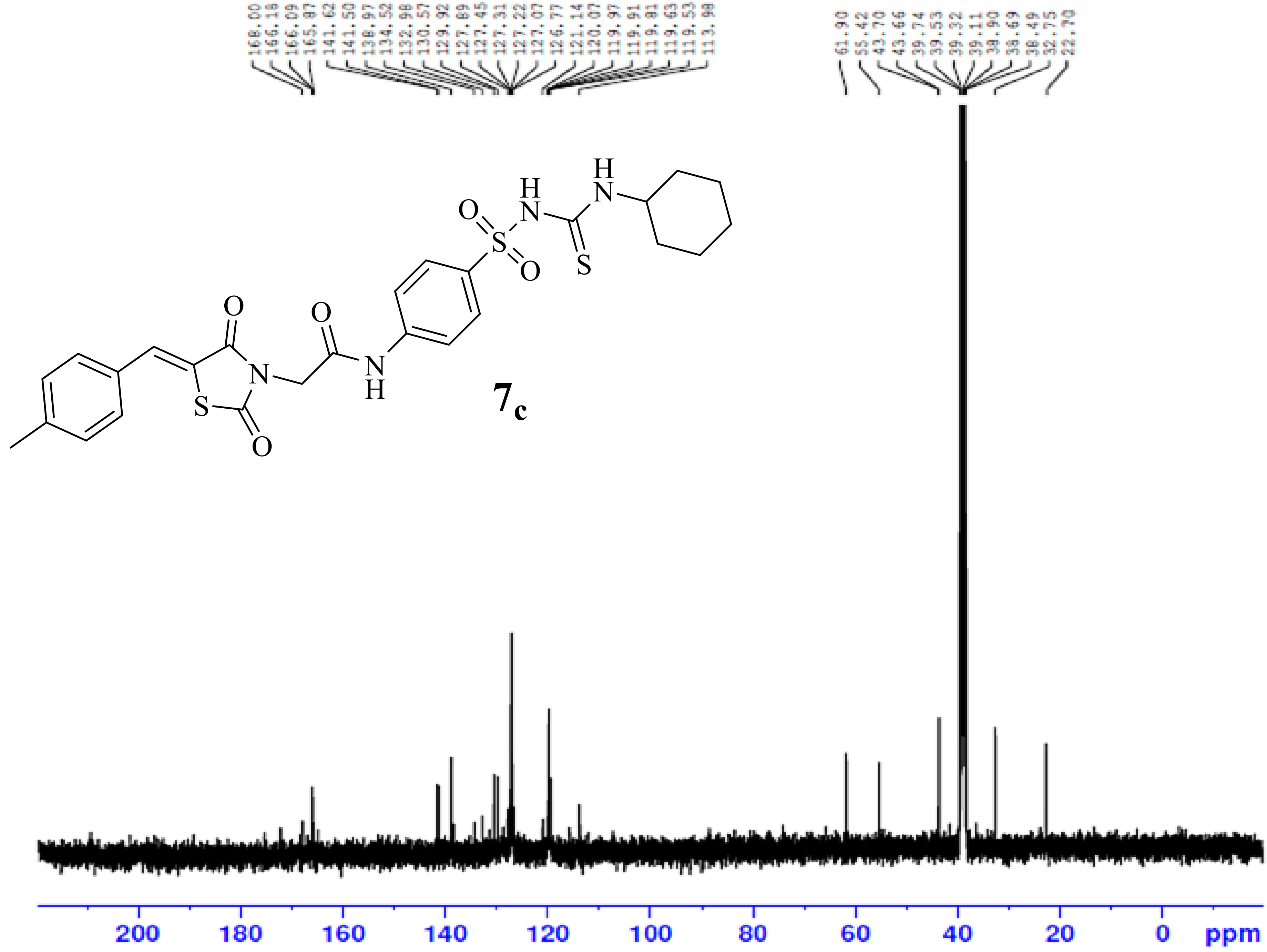

Current Data Parameters
NAME KE-E
EXPNO 2
PROCNO 1

F2 - Acquisition Parameters
Date_ 20200927
Time 10.27
INSTRUM spect
PROBHD 5 mm PABBO BB/
PULPROG zgpg30
TD 65536
SOLVENT DMSO
NS 1783
DS 4
SWH 24038.461 Hz
FIDRES 0.366798 Hz
AQ 1.3631488 sec
RG 205.37
DW 20.800 usec
DE 6.50 usec
TE 300.0 K
D1 2.00000000 sec
D11 0.03000000 sec
TD0 1

======== CHANNEL f1 ========
SFO1 100.6278588 MHz
NUC1 13C
P1 10.00 usec
PLW1 47.00000000 W

======== CHANNEL f2 ========
SFO2 400.1516006 MHz
NUC2 1H
CPDPRG[2 waltz16
PCPD2 90.00 usec
PLW2 18.00000000 W
PLW12 0.34722000 W
PLW13 0.28125000 W

F2 - Processing parameters
SI 32768
SF 100.6177975 MHz
WDW EM
SSB 0
LB 1.00 Hz
GB 0
PC 1.40

khaled-aladel-H #36 RT: 0.62 AV: 1 SB: 2 2.24 , 2.34 NL: 5.43E2
T: {0,0} + c EI Full ms [40.00-1000.00]

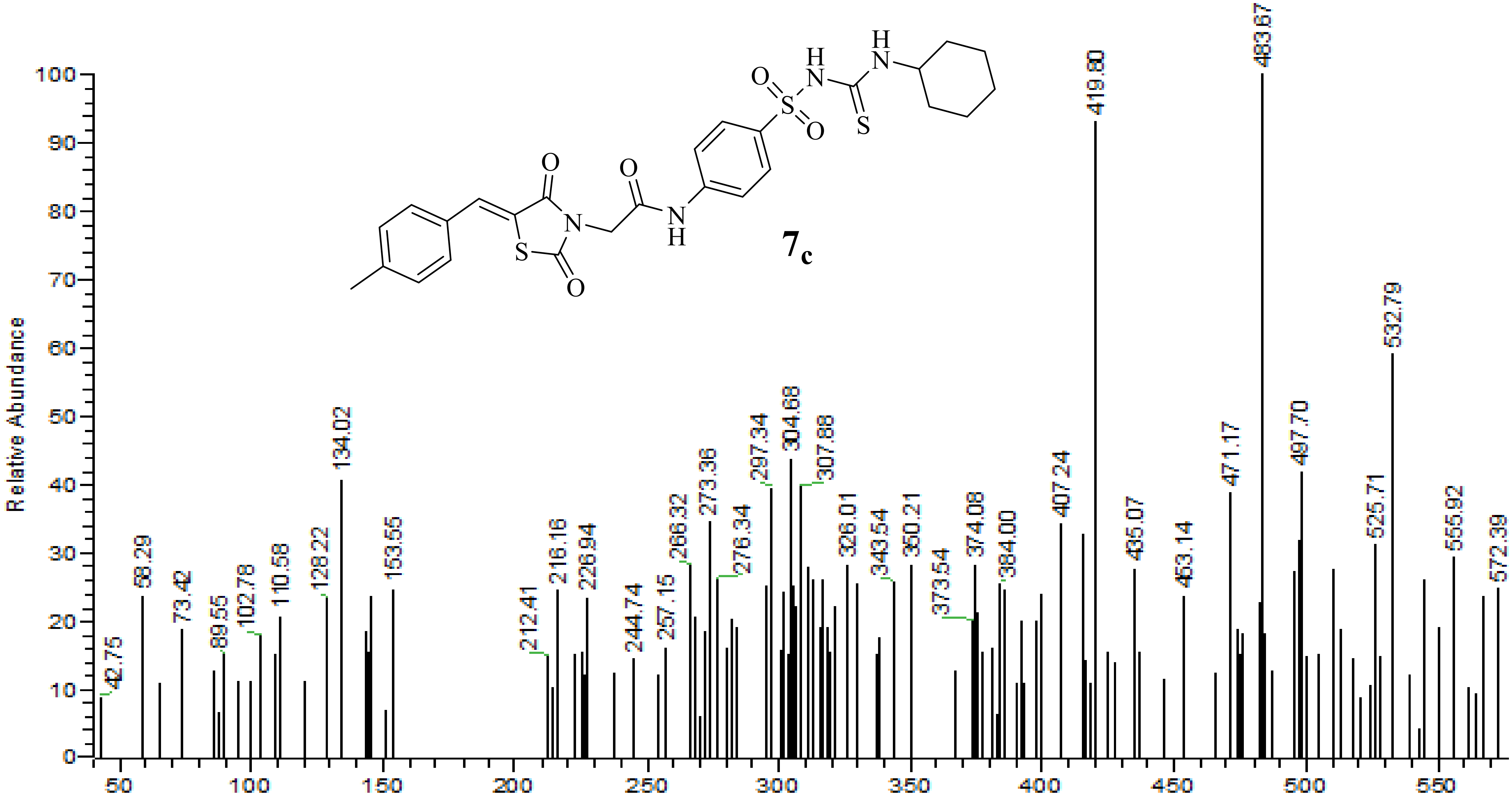

Supplement: Supplementary file 1 [file pharmaceuticals-15-00226-s001.zip › pharmaceuticals-1563407-supplementary.pdf]
